# Microbial Adhesion and Biofilm Formation on Bioactive Surfaces of Ti-35Nb-7Zr-5Ta Alloy Created by Anodization

**DOI:** 10.3390/microorganisms9102154

**Published:** 2021-10-15

**Authors:** Laiza Maria Grassi Fais, Luana de Sales Leite, Bárbara Araújo dos Reis, Ana Lúcia Roselino Ribeiro, Luis Geraldo Vaz, Marlise Inêz Klein

**Affiliations:** 1 Department of Dental Material and Prosthodontics, School of Dentistry, São Paulo State University (UNESP), Araraquara 14800900, Brazil; lusales94@gmail.com (L.d.S.L.); geraldo.vaz@unesp.br (L.G.V.); 2 Department of Diagnosis and Surgery, School of Dentistry, São Paulo State University (UNESP), Araraquara 14800900, Brazil; barbara.araujo@unesp.br; 3 Faculdade de Ciências do Tocantins, Centro Universitário Tocantinense Presidente Antônio Carlos (UNITPAC), Araguaína 77816540, Brazil; analuciaroselino@gmail.com

**Keywords:** titanium, topography, biofilms, surface properties, coatings

## Abstract

This study evaluated the microbial colonization (adhesion and biofilm) on modified surfaces of a titanium alloy, Ti-35Nb-7Zr-5Ta, anodized with Ca and P or F ions, with and without silver deposition. The chemical composition, surface topography, roughness (Ra), and surface free energy were evaluated before and after the surface modifications (anodizing). Adhesion and biofilm formation on saliva-coated discs by primary colonizing species (*Streptococcus sanguinis*, *Streptococcus gordonii*, *Actinomyces naeslundii*) and a periodontal pathogen (*Porphyromonas* *gingivalis*) were assessed. The surfaces of titanium alloys were modified after anodizing with volcano-shaped micropores with Ca and P or nanosized with F, both with further silver deposition. There was an increase in the Ra values after micropores formation; CaP surfaces became more hydrophilic than other surfaces, showing the highest polar component. For adhesion, no difference was detected for *S. gordonii* on all surfaces, and some differences were observed for the other three species. No differences were found for biofilm formation per species on all surfaces. However, *S. gordonii* biofilm counts on distinct surfaces were lower than *S. sanguinis*, *A. naeslundii*, and *P. gingivalis* on some surfaces. Therefore, anodized Ti-35Nb-7Zr-5Ta affected microbial adhesion and subsequent biofilm, but silver deposition did not hinder the colonization of these microorganisms.

## 1. Introduction

Despite high success and survival rates with reliable long-term results, implant rehabilitation shows early and late failures that should be minimized. Annually, approximately 5.5 million dental implants are placed; the dental implant and prosthetic market in the U.S. were projected to reach $6.4 billion by 2018 [1]. Thus, mainly due to the population aging, these devices should stay longer in function, often in poor quality bones, compromising the biomechanical request at the bone/titanium interface [2].

In this context, researches on biomaterials and the implant systems industry have focused interest in developing (i) innovative alloys, ideally free of aluminum or vanadium, which have better compatibility, both biological (absence of toxicity) and mechanical (lower elastic modulus) [3,4,5]; and (ii) surface modifications to improve the regenerative capacity of the adjacent bone, providing biological stimuli at the implant interface [6,7,8], but at the same time inhibiting biofilm formation if the implant is exposed

In the oral cavity. Since the 1990s, the challenge of surface modification has been proposed. Several methods were studied since the first-generation implant surface created by machining, including plasma spray coating, grit blasting, acid etching, sandblasted and acid etching (SLA), anodizing and biomimetic coating [9,10].

Anodizing is the dielectric breakdown of the TiO_2_ layer by applying an increased voltage to generate a micro-arc. This process is considered an important method because it is a well-established, simple, versatile, scalable, and low-cost that allows the formation of different structures ranging from micro to nanometric scale on the surface of titanium and its alloys [6].

Different anodizing conditions are reported in the literature. The most evaluated electrolytes are composed of calcium [11,12], sulfuric acid [13], acid [14], hydrogen fluoride [15], sodium hydroxide [16] and hydrofluoric acid [17,18,19,20]. The first anodized implant marketed with the most substantial number of cohort studies that aimed to evaluate implants success rates was TiUnite (Nobel Biocare, Zurich, Switzerland), whose surface has a moderately rough crystalline oxide layer with a high content of phosphorus [21].

These moderately rough surfaces with microporous topography increase the bioactivity of the implants [22] by facilitating the anchoring of osteocytes and, consequently, the formation of bone tissue [23,24]. However, it remains uncertain whether the exposure of anodized surfaces designed to improve osseointegration to the oral biofilm indeed can be associated with reports of higher incidence of retrograde peri-implantitis [25], indicating significant gaps in knowledge and the need for simplified and fundamental in vitro studies about the initial stages of adhesion and formation of biofilm on anodized titanium surfaces.

Several microbiological studies investigated the differences between healthy sites and sites diagnosed with peri-implantitis, associated or not with implant loss. The former is characterized by a microbiota dominated by Gram-positive cocci and bacilli (e.g., *Streptococcus sanguinis*, *Streptococcus oralis*, *Streptococcus gordonii*, *Actinomyces naeslundii*). In contrast, the presence of Gram-negative anaerobic bacteria (e.g., *Porphyromonas gingivalis*, *Prevotella intermedia*, *Prevotella nigrescens*, *Tannerella forsythia*, *Campylobacter rectus*, *Aggregatibacter actinomycetemcomitans*) is associated with the development of peri-implant diseases, similar to periodontitis [26], with fewer bacterial cells in peri-implant mucositis [27,28,29,30].

Concerning the anodizing method, a possible surface modification consists of incorporating silver (Ag) particles on the pores or nanotube of the oxide layer. Ag particles can: (I) react with water, releasing ions that combine with sulfhydryl groups of the respiratory enzyme or nucleic acids of the bacteria, blocking the microbial respiration [31] or (II) activate the oxygen of the water, making it able to decompose the bacteria by oxidative stress, inactivating the microbial metabolism [32]. Titanium surfaces modified with silver deposition could provide antibacterial properties, avoiding postoperative infections, without interfering in the adhesion and proliferation of bone tissue [33] and without causing cytotoxicity [34].

Hence, the effects of surface modification via anodization for bioactive surfaces with pores (additive method) or nanotubes (subtractive method) warrants further information on microbial colonization. Therefore, this study aimed to evaluate microbial adhesion and subsequent biofilm formation of initial colonizers (*S. sanguinis*, *S. gordonii*, and *A. naeslundii*) and a periodontal pathogen (*P. gingivalis*) on anodized Ti-6Al-4V and Ti-35Nb-7Zr-5Ta surfaces. The anodization comprised electrolytes with calcium (Ca) and phosphorous (P), hydrofluoric acid (HF), besides the deposition of silver. These surfaces have been evaluated for cytotoxicity, corrosion resistance, and biocompatibility [3,10].

## 2. Materials and Methods

A flowchart of this study is shown in Figure S1. Saliva collection for salivary pellicle preparation was approved by the Institutional Ethical Committee (CAAE: 50795615.4.0000.5416).

Three volunteers agreed to participate in this study with saliva donation. One woman and two men (an average of 32 years old), who did not use antibiotics in the last three months, provided their consent and signature. Saliva was collected in the morning after rinsed the mouth with 5 mL of distilled H_2_O. A piece of parafilm (Parafilm M, Laboratory Film, Atlanta, GA, USA) was chewing to stimulated saliva; the first 5 mL was discarded. A saliva pool from all volunteers was carried out and diluted (1:1 in vol) with adsorption buffer (AB buffer; 50 mmol/L KCl, 1 mmol/L KPO_4_, 1 mmol/L CaCl_2_, 1 mmol/L MgCl_2_, 0.1 mmol/L phenylmethylsulfonyl fluoride, in dd H_2_O, pH 6.5; all these reagents were from Sigma Aldrich, St. Louis, MO, USA). It was clarified by centrifugation (5000× *g*, 10 min), the supernatant was sterilized (0.22 μm low protein binding, polyethersulfone membrane filter; Nalgene™ Rapid-Flow™, Thermo Fisher Scientific, Waltham, MA, USA) and stored at −80 °C until used.

### 2.1. Samples Preparation

Two titanium alloys were used in this study, Ti-6Al-4V ELI (TAV) and Ti-35Nb-7Zr-5Ta (%wt) (TNZT). TAV discs (8 mm Ø × 2 mm thickness) were obtained by machining commercial bars (Realum Indústria e Comércio de Metais Puros e Ligas Ltd.a, São Paulo, Brazil). The starting materials to obtain TNZT alloy (Ti (Realum Indústria e Comércio de Metais Puros e Ligas Ltd.a, São Paulo, Brazil) Nb (CBMM Companhia Brasileira de Metalurgia e Mineração, Minas Gerais, Brazil) Zr (Sigma Aldrich) and Ta (Sigma Aldrich), with a degree of purity greater than or equal to 99.00%) were arc melted in an argon atmosphere, remelted (3 to 5 times) to ensure homogeneity and, vacuum heat-treated (1000 °C, 8 h, furnace cooled). They were hot-swaged into bars (≈11 mm Ø) and machined into discs with 8 mm diameter and 2 mm thick. These discs were vacuum heat-treated at 1000 °C for 1 h and air-cooled to relieve stress and tensions [35].

All discs were mechanically polished with silicon carbide abrasive papers (Hermes Schleifmittel GmbH and Co, Hamburg, Germany) with #320, #600, #800 #1200, #1500 and #2500 (for 40 s each). They were cleaned in an ultrasonic bath with isopropyl alcohol for 10 min and air-dried. The discs were etched for 8 s with Kroll’s reagent (distilled water, 75% nitric acid (Sigma Aldrich), and 45% hydrofluoric acid (Sigma Aldrich); 1:1:1 in vol) and were cleaned again to remove contaminants and the original passive oxide layer [35]. The discs were divided into two groups, TAV (Ti-6Al-4V) or TNZT (Ti-35Nb-7Zr), and assigned to subgroups as listed in Table 1.

### 2.2. Anodization

Anodization was carried out with magnetic stirring, using the potentiostatic method and 140 mL of each electrolyte at room temperature of 25 °C. The discs (anode) remained at 8 mm distance from the cathode (stainless steel plate). After each anodization, the discs were cleaned in an ultrasonic bath with isopropyl alcohol (Sigma Aldrich) for 10 min and air-dried.

### 2.3. Surface Characterization

Overall surface morphology was characterized by scanning electron microscopy (SEM, JEOL JSM-6610LV, Tokyo, Japan) with secondary electrons and high-resolution field emission microscopy (FEG-JSM-7500F, JEOL, Tokyo, Japan). In addition, the chemical composition of the surfaces was assessed by an Energy Dispersive X-ray Spectroscopy (EDS) coupled to the FEG. Discs were placed directly onto the stub and examined without any preparation or manipulation. SEM images were processed using the software ImageJ (National Institutes of Health; http://imagej.nih.gov/ij/download.html, accessed on June 2018) to measure the pores and nanotubes.

The surface roughness was measured with a roughness analyzer (Surftest SJ-401; Mitutoyo Corp, Wilmington, NC, USA) with an accuracy of 0.01 μm, a read length of 2.5 mm and a speed of 0.5 mm/s. Five measurements of Ra (mean roughness) were performed on each surface, with 10 discs for each subgroup, and the mean values were calculated.

The surface free energy (SFE) was analyzed using the sessile drop method with a goniometer (Ramé-Hart 10000; Ramé-hart instrument co., Succasunna, NJ, USA) with and without salivary coating. Discs were immersed in 1.5 mL of sterilized saliva for 2 h at 37 °C, washed in distilled water and dried at 30 °C for 15 min [36]. The contact angle was measured using fluids differing in hydrophobicity (distilled water, glycerol (Sigma Aldrich) and diiodomethane (Sigma Aldrich)) [37], at a controlled temperature (25 °C), and after the settling time (30 s) of the drop (15 μL). In this case, 10 discs of each subgroup (*n* = 100) were measured three times, and the average of each surface and fluid was analyzed as described by Owens and Wendt in the software SCA 20 (DataPhysics Instruments GmbH). The same procedures were carried out without immersion in sterilized, clarified whole saliva (i.e., with and without salivary pellicle).

### 2.4. Microbiological Tests

The discs were subjected to gamma radiation sterilization at 25 kGy. Adhesion and biofilm assays were performed in duplicate on three different experimental occasions. The strains *S. sanguinis* SK36, *S. gordonii* DL1, *A. naeslundii* ATCC 12104 and *P. gingivalis* ATCC 33277 were used for single-species biofilm formation. The initial colonizer species *A. naeslundii*, *S. sanguinis* and *S. gordonii* bind to complementary salivary receptors [30]. *A. naeslundii* is compatible with periodontal health that participates in the co-aggregation of secondary colonizers [38]. *P. gingivalis* is an anaerobic bacterium that belongs to the red complex, involved in the development of peri-implantitis [38,39].

*S. sanguinis*, *S. gordonii* and *A. naeslundii* were grown separately on blood agar plates (5% sheep’s blood; Laborclin, Pinhais, PR, Brazil) (48 h/37 °C/5% CO_2_; Steri-Cult™Thermo Scientific, Waltham, MA, USA). In this case, 5 to 10 colonies of each microorganism were inoculated into 10 mL of tryptone with yeast extract (TY: 2.5% tryptone, 1.5% yeast extract, pH 7.0; Difco) containing 1% glucose (Synth) and incubate (37 °C/5% CO_2_). After 16 h, 1:20 dilutions were carried out and incubated (37 °C/5% CO_2_) until end of the exponential growth phase (OD600 nm 0.962 ± 0.082 for *S. sanguinis*, OD600 nm for 0.998 ± 0.012 for *S. gordonii* and OD600 nm 1.48 ± 0.028 for *A. naeslundii;* spectrophotometer SP-220 BIOSPECTRO).

*P. gingivalis* was plated onto Brucella agar (Becton Dickinson, Heidelberg, Germany) containing 1% of hemin (Sigma Aldrich) and 0.05% of menadione (Calbiochem-Novabiochem, La Jolla, CA, USA) and kept 48 h at 37 °C inside an anaerobic incubator with an oxygen-free atmosphere (85% N_2_, 10% H_2_, 5% CO_2_) (Don Whisthey, Shipley, England). In this case, 5 to 10 colonies were inoculated into 10 mL of TYE supplemented with 1% hemin, 0.05% menadione and 1% glucose (Synth). After 18 h, 1:20 dilution was prepared and incubated in an anaerobic incubator until the end of the exponential growth phase (OD600 nm 1.151 ± 0.104). The purity of cultures was checked with Gram staining.

Upon reaching the desired growth phase, each inoculum with a defined microbial population (5 × 10^6^ colony-forming units-CFU/mL) of *S. sanguinis*, *S. gordonii*, *A. naeslundii* and *P. gingivalis* was prepared.

Before the bacteria inoculation, the discs were individually placed into 24-well plates (Kasvi, Kasvi, São José do Pinhais, Brazil) containing 1.5 mL human stimulated clarified whole saliva filtered-sterilized and incubated (37 °C, 75 rpm, 1 h) to generate the salivary pellicle [40]. Then, the saliva was removed, and discs were washed twice with 2 mL AB buffer.

Saliva-coated discs were covered with 1.5 mL inoculum. They remained 90 min at 37 °C and 5% CO_2_ (*S. sanguinis*, *S. gordonii* and *A. naeslundii*) or 48 h at 37 °C inside an anaerobic incubator with an oxygen-free atmosphere (*P. gingivalis*) for microorganism adhesion. Discs were washed twice with 2 mL of 0.89% NaCl (Synth) to remove unattached microorganisms. For the adhesion assays, half of the discs were processed to quantify the CFU/disc. For the biofilm assays, the others discs with adhered bacterial cells were transferred to 24-well plates with fresh culture medium (TYE + 1% glucose for *S. sanguinis*, *S. gordonii* and *A. naeslundii*; TYE + 1% hemin + 0.05% menadione for *P. gingivalis*) and incubated for 48 h at 37 °C, 5% CO_2_ (*S. sanguinis*, *S. gordonii* and *A. naeslundii*) or 120 h at 37 °C with an oxygen-free atmosphere (*P. gingivalis*). The growth medium was refreshed every 24 h. Then, discs were washed twice with 2 mL of 0.89% NaCl (Synth) and processed to quantify biofilm as CFU/disc.

At the end of the adhesion and biofilm assays, each disc was transferred to a glass tube to harvest the microorganisms. The walls of these tubes were washed with 2 mL of 0.89% NaCl, and the tubes were submitted to water bath sonication (Kondor-tech Digital Ultrasonic Cleaner, Kondoertech Indústria e Comércio Ltd.a., São Carlos, Brazil) for 15 min to remove adhered cells and biofilms from the surfaces. The suspension in each tube (2 mL) was transferred to a 15 mL Falcon tube (Kasvi). The glass tube walls were washed with 1 mL 0.89% NaCl and transferred to the Falcon tube, yielding a 3-mL total adhesion or biofilm suspension per disc. These suspensions were sonicated at 7 W during 30 s (Q125, Q Sonica, Newtown, CT, USA) and submitted to 10-fold serial dilution. *S. sanguinis*, *S. gordonii* and *A. naeslundii* suspensions were plated on blood agar (37 °C, 5% CO_2_, 48 h) while *P. gingivalis* was plated on Brucella agar containing 1% of hemin and 0.05% of menadione (37 °C, anaerobiosis, 120 h).

Biofilms were also characterized via SEM. Additional experiments for biofilm formation were performed as described above. At the end of the biofilm assay (48 h or 120 h), the discs were washed twice with 2 mL 0.89% NaCl, fixed with 2.5 % glutaraldehyde (pH 7.4) at room temperature for 1 h, and dehydrated with standard ethanol series: 70% (60 min), 90% (60 min), 100% (5 × 30 min) (Sigma Aldrich). Discs were kept under vacuum for seven days and were sputter-coated with gold to perform SEM (JEOL JSM-6610LV).

### 2.5. Statistical Analyses

Ra, SFE and CFU/disc data were analyzed using GraphPad Prism 7 software (2016; GraphPad Software, Inc., San Diego, CA, USA). The data normality distribution was tested by Shapiro-Wilk. Two-way ANOVA with Tukey post-test was used to clarify whether titanium alloys (TAV and TNZT) or surface modifications (CaP, CaPAg, HF, HFAg) had a similar behavior regarding Ra, SFE, initial adhesion and biofilm formation. The level of significance was set at 5%.

## 3. Results

### 3.1. Surface Characterization before Microbial Colonization

**Figure 1:**
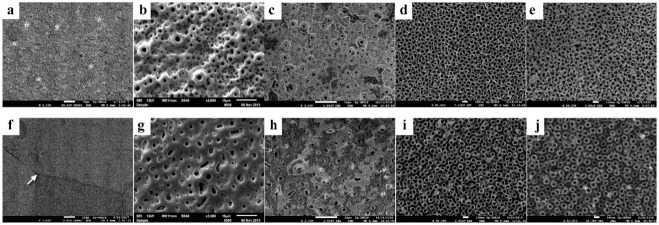
Scanning electron microscopy images of the different surfaces. (**a**) Kroll etched (control) TAV; (**b**) CaP TAV; (**c**) CaPAg TAV; (**d**) HF TAV; (**e**) HFAg TAV; (**f**) Kroll etched (control) TNZT; (**g**) CaP TNZT; (**h**) CaPAg TNZT; (**i**) HF TNZT; (**j**) HFAg TNZT. Asterisks show the β phase of TAV. Arrow indicates grain boundary in TNZT.

The micrographs of TAV and TNZT are shown in Figure 1. Different surface characteristics for the two types of titanium alloys were observed in the control surfaces (Figure 1a,f) TAV shows heterogeneous granular characteristics resulting from Kroll etching, with the α (dark) and β (light) phases that characterize this titanium alloy (Figure 1a). On the other hand, a more homogeneous surface with evident grain boundary and only β phase could be seen in the control TNZT surface (Figure 1f).

**Figure 2:**
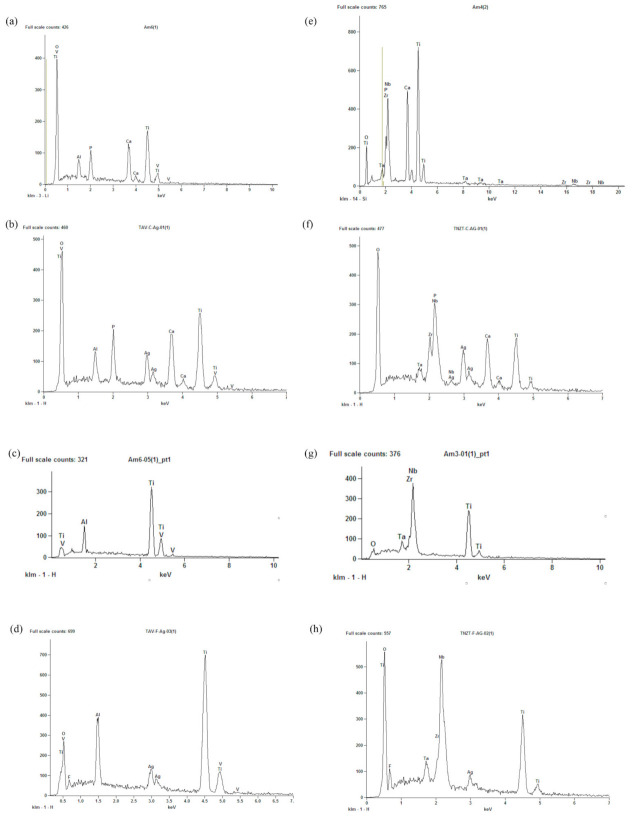
EDS spectra of the qualitative chemical composition. (**a**) CaP TAV; (**b**) CaPAg TAV; (**c**) HF TAV; (**d**) HFAg TAV; (**e**) CaP TNZT; (**f**) CaPAg TNZT; (**g**) HF TNZT; (**h**) HFAg TNZT.

The surfaces of both titanium alloys were modified after anodizing with sodium β-glycerophosphate + calcium acetate (Figure 1b,g), forming multilayers with volcano-shaped micropores. The chemical composition (qualitative) obtained by EDS indicated the incorporation of calcium and phosphorus ions on these surfaces (Figure 2a,e).

Anodizing with hydrofluoric acid resulted in nanotubular surfaces (Figure 1d,i), with nanosized pores averages of 14.33 ηm and 16.21 ηm in diameter, respectively, in TAV (Figure 1d) and TNZT (Figure 1i). The presence of fluoride ions was identified in HFAg surfaces (Figure 2d,h and Figures S2 and S3).

The two-step anodization for the incorporation of silver ions resulted in the deposition of silver nanoparticles on the micropores (Figure 1c,h) and the nanotubes (Figure 1e,j). Its presence was confirmed, quantified and mapped using X-ray dispersive energy (EDS), as shown in Figure 2b,d,f,h and Figures S2 and S3.

Ra means are shown in Table 2. According to the criteria of Wennerberg and Albrektsson (2010) [41], the surfaces of Control, HF and HFAg were classified as smooth, while those of CaP and CaPAg were minimally rough. CaP anodizing increased the roughness of TAV and TNZT that were similar. On the other hand, HF surfaces had the Ra values equal to the control both for TAV and TNZT. Ag statistically increased the Ra of TNZT HFAg. The Ra values of TNZT CaPAg, HF and HFAg were higher than the correspondent TAV groups. 

Table 3 and Table 4 show the SFE values, respectively, without and with a salivary pellicle. TAV HF and TAV HFAg surfaces had higher SFE than control; for TNZT, CaP was equal to control. Silver deposition did not change the SFE in TAV surfaces but increased the values in TNZT CaPAg surfaces. After salivary pellicle coating, only TAV CaP and TNZT CaP had values higher than controls (TAV control and TNZT control). Silver incorporation did not change the SFE values (CaP = CaPAg; HF = HFAg). There is an inversion between the values of the dispersive and polar phases when the surfaces with and without salivary coating are compared.

### 3.2. Microbial Adhesion to and Biofilm Formation on Surfaces

**Figure 3:**
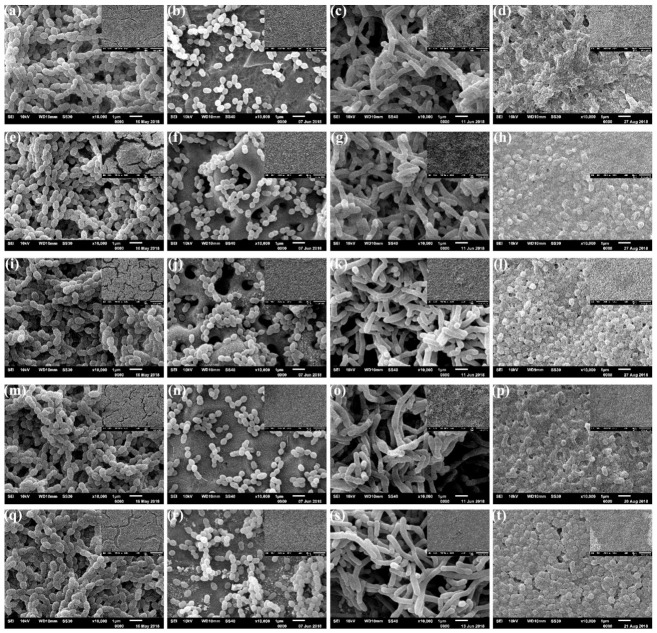
SEM images of the TAV surfaces after single-species biofilm formation of *S. gordonii* (first column), *S. sanguinis* (second column), *A. naeslundii* (third column) and *P. gingivalis* (fourth column). Rows correspond to the surface treatments: control (**a**–**d**), CaP (**e**–**h**), CaPAg (**i**–**l**), HF (**m**–**p**) and HFAg (**q**–**t**). The highest magnification (×10,000) corresponds to the central regions of the micrographs, with the smallest magnification (×500) located in the upper right corner.

**Figure 4:**
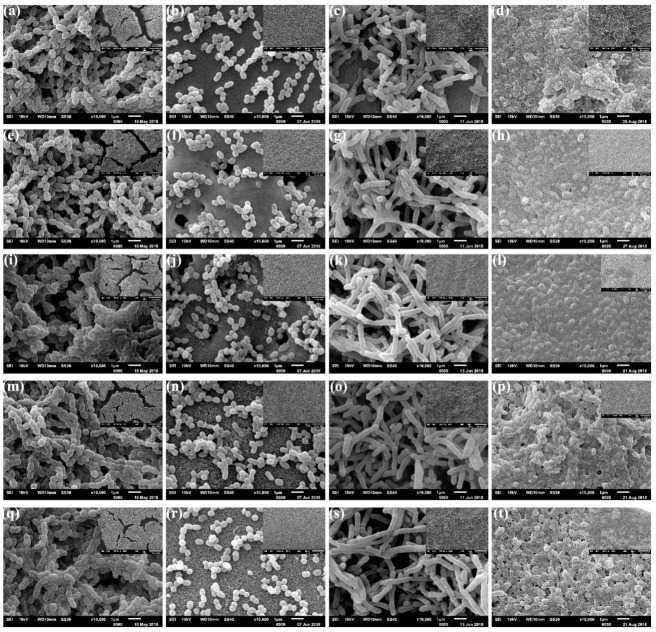
SEM images of the TNZT surfaces after single-species biofilm formation of *S. gordonii* (first column), *S. sanguinis* (second column), *A. naeslundii* (third column) and *P. gingivalis* (fourth column). Rows correspond to the surface treatments: (**a**–**d**), CaP (**e**–**h**), CaPAg (**i**–**l**), HF (**m**–**p**) and HFAg (**q**–**t**). The highest magnification (×10,000) corresponds to the central regions of the micrographs, with the smallest magnification (×500) located in the upper right corner.

SEM images of the surfaces after biofilm formation are shown in Figure 3 and Figure 4. All surfaces were colonized with coccus (*S. gordonii*, *S. sanguinis*), bacillus (*A. naeslundii*) and coccobacilli (*P. gingivalis*), with no differences between the titanium alloy types. However, different distribution patterns could be seen between *S. gordonii* and *S. sanguinis* species (Figure 3 and Figure 4, first and second columns).

A denser biofilm of *S. gordonii* (Figure 3 and Figure 4, first column) was formed after 48 h. These surfaces were entirely covered by a discontinuous biofilm that hinders the view of the metallic surfaces at both smaller (×500) and higher (×10,000) magnification. On the other hand, after the same period, surfaces inoculated with *S. sanguinis* (Figure 3 and Figure 4, second column) exhibit multicellular agglomerates, small chains or even isolated coccus, allowing the view of the metal surfaces.

The surfaces colonized with *A. naeslundii* (Figure 3 and Figure 4, third column) exhibit some differences after Ag deposition. Control (Figure 3c and Figure 4c), CaP (Figure 3g and Figure 4g) and HF (Figure 3o and Figure 4o) surfaces show the biofilm formation. In comparison, CaPAg (Figure 3k and Figure 4k) and HFAg (Figure 3s and Figure 4s) show distinct aggregates of cells evenly distributed over the entire surface. At higher magnification, all surfaces exhibit branching rods cells.

The surfaces colonized by *P. gingivalis* (Figure 3 and Figure 4, fourth column) also showed some differences concerning surface treatments. Those with silver deposition (Figure 3l,t and Figure 4 l,t) had more defined cells with evident fimbriae. In addition, the HF surfaces (Figure 3h,l and Figure 4 h,l) had a more homogeneous biofilm coating, CaP surfaces exhibit a more sparse distribution of biofilms (Figure 3p,t and Figure 4 p,t). Furthermore, it is possible to observe extracellular substances between *P. gingivalis* cells, which were not visible in the images from the other species.

**Figure 5:**
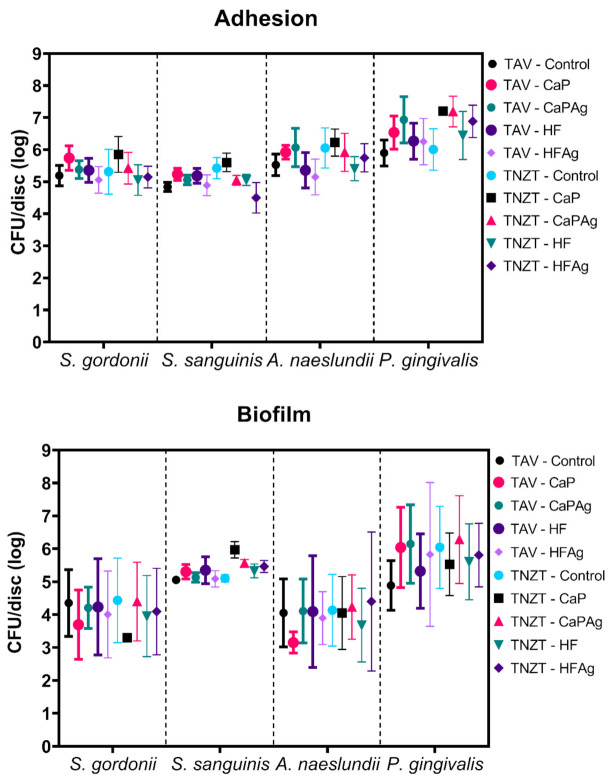
Microbial adhesion and biofilm formation (log CFU/disc) on the distinct surfaces. The data show are the means and the error bars are the 95% confidence interval. Adhesion presented significant interaction (*p* = 0.0011; bacterial species *p* < 0.0001; surfaces *p* < 0.0001), while biofilm did not (*p* = 0.7982; bacterial species *p* < 0.0001; surfaces *p* = 0.5811). Detailed statistical analyses outputs are presented in Table S1 (adhesion data) and Table S2 (biofilm data).

Figure 5 shows the quantification (CFU/disc) of *S. gordonii*, *S. sanguinis*, *A. naeslundii* and *P. gingivalis* for adhesion and biofilm assays. Regarding adhesion, Two-way ANOVA considering as factors “bacterial species” and “surfaces” yielded a significant interaction (*p* < 0.0001). No difference was detected for *S. gordonii* adhesion on all surfaces tested, and some differences were seen for the other three species. *S. sanguinis* adhered in lower numbers to TNZT-HFAg, but that quantity was statistically different only *versus* TNZT-CaP (*p* = 0.0089). *A. naeslundii* presented lower counts for TAV-HFAg, which was statistically different from TNZT-CaP (*p* = 0.0134). The adhesion of *P. gingivalis* was hindered by TAV-control compared to TAV-CaPAg (*p* = 0.0241), TNZT-CaP (*p* = 0.0030), TNZT-CaPAg (*p* = 0.0004) and TNZT-HFAg (*p* = 0.0469). In addition, TNZT-control yielded a lower amount of *P. gingivalis* than TNZT-CaP (*p* = 0.0252) and TNZT-CaPAg (*p* = 0.0060). Thus, the surface modifications increased the adhesion CFU/discs values of *P. gingivalis* in TAV CaPAg, TNZT CaP and TNZT CaPAg. Therefore, modifications of TAV and TNZT via anodization applied in this study affected adhesion depending on the bacterial species, where some modifications hindered it while others promoted it. Moreover, comparisons between the four species and all surfaces indicated some differences (as detailed in the Supplementary Table S1).

Regarding biofilm formation, no differences were observed per species on all surfaces tested. However, comparisons between the four species and all surfaces indicated some differences (as detailed in the Supplementary Table S2). *S. gordonii* biofilm CFU counts on distinct surfaces were lower than of *S. sanguinis*, *A. naeslundii* and *P. gingivalis* on some surfaces. Specifically, *S. gordonii* biofilm CFU on TAV-CaP were lower than *S. sanguinis* on TNZT-CaP (*p* = 0.0267) and *P. gingivalis* on TAV-CaP (*p* = 0.0168), TAV-CaPAg (*p* = 0.0080), TNZT-Control (0.0162), TNZT-CaPAg (*p* = 0.0030). In addition, *S. gordonii* biofilm CFU counts on TAV-HFAg were lower than *P. gingivalis* on TNZT-CaPAg (*p* = 0.0267). Similarly, *S. gordonii* counts on TNZT-CaP were lower than for *S. sanguinis* on TNZT-CaP (*p* = 0.0040) and *P. gingivalis* on TAV-CaP (*p* = 0.0024), TAV-CaPAg (*p* = 0.0011), TAV-HFAg (*p* = 0.207), TNZT-Control (*p* = 0.0023), TNZT-CaPAg (*p* = 0.0004), TNZT-HF (*p* = 0.0417) and TNZT-HFAg (*p* = 0.0235). In addition, *S. gordonii* counts on TNZT-HF were lower than for *P. gingivalis* on TAV-CaPAg (*p* = 0.0439) and TNZT-CaPAg (*p* = 0.0191). Finally, *S. gordonii* counts on TNZT-HFAg were lower than *P. gingivalis* on TNZT-CaPAg (*p* = 0.0457). On the other hand, *S. sanguinis* biofilm CFU counts on TNZT-CaPAg were higher than for *A. naeslundii* on TAV-CaP (*p* = 0.0050) and TNZT-HF (*p* = 0.0245).

Furthermore, *A. naeslundii* biofilm CFU counts on distinct surfaces were lower than of *P. gingivalis* on some surfaces: *A. naeslundii* on TAV-Control was less abundant than *P. gingivalis* on TNZT-CaPAg (*p* = 0.0354) while *A. naeslundii* on TAV-Control presented lower counts compared to *P. gingivalis* on TAV-CaP (*p* = 0.0031), TAV-CaPAg (*p* = 0.0015), TAV-HFAg (*p* = 0.0218), TNZT-Control (*p* = 0.0030), TNZT-CaPAg (*p* = 0.0006), TNZT-HF (*p* = 0.0436) and TNZT-HFAg (*p* = 0.0247). In addition, *A. naeslundii* on TAV-CaPAg was less abundant than *P. gingivalis* on TNZT-CaPAg (*p* = 0.0499). *A. naeslundii* on TAV-HF presented lower counts than *P. gingivalis* on TNZT-CaPAg (*p* = 0.0457), while the addition of silver to TAV-HF (TAV-HFAg) also yielded less *A. naeslundii* counts compared to *P. gingivalis* on TAV-CaPAg (*p* = 0.0306) and TNZT-CaPAg (*p* = 0.0129). *A. naeslundii* was less abundant on TNZT-CaP than *P. gingivalis* on TNZT-CaPAg (*p* = 0.0354). Lastly, the *A. naeslundii* counts on TNZT-HF were lower than *P. gingivalis* on TAV-CaP (*p* = 0.0153), TAV-CaPAg (*p* = 0.0072), TNZT-Control (*p* = 0.0148) and TNZT-CaPAg (*p* = 0.0028). Thus, the incorporation of silver decreased only *S. sanguinis* adhesion in TNZT (CaP vs. CaPAg and HF vs. HFAg) and *S. sanguinis* biofilm formation in CaPAg surfaces of TNZT.

## 4. Discussion

Mucositis and peri-implantitis are the major pathologic complications observed in dental implantology, remaining a challenge in the area [42]. Dental implants are promptly colonized by microorganisms after their placement, with some similarities in the microbiota composition to that occurring on natural teeth [43]. In the event of retraction of the bone level, biofilm attachment to the exposed surfaces will be influenced by physicochemical characteristics of the implant surfaces such as roughness, surface free energy, hydrophobicity and charge [42,44] and also by localized host factors as serum, salivary pellicle [45] and proteins [46]. Therefore, it is paramount to understand the microbial colonization (adhesion and consequent biofilm development) of modified surfaces produced by anodization here named CaP, CaPAg, HF and HFAg.

CaP and CaPAg surfaces exhibited micropores (Figure 1b,c,g,h) with diameters comparable to the volcano-shaped marked dental implants (1–2 μm) [47,48]. In this process, the anodizing parameters were an additive modifying method of the surfaces. When the redox reactions occur, the temperature in the metal/electrolyte interface increase and release oxygen and/or water vapor, creating the micropores layers [6]. Furthermore, because electrical discharge attracts the electrolyte ions to the surface, the redox reactions allow the incorporation of ions, Ca, P and Ag as indicated in EDS spectra (Figure 2) and maps (Figures S2 and S3).

In contrast, the anodization with hydrofluoric acid is a subtractive surface modifying method that allows the morphology of nanotubes with diameters ranging from 25 to 500 ηm depending on the type of titanium alloy, concentration of the hydrofluoric acid, intensity of current and anodizing time [17,18,19,20,49]. First, a compact titanium dioxide layer is created; then, soluble fluoride complexes are formed through the chemical dissolution of the oxide, increasing in current. The reaction continues until it reaches the equilibrium between oxidation and dissolution. At this time, the current flow remains constant, and the nanostructured surface is formed [7].

The increase in Ra values after anodizing (Table 2) with β-sodium glycerophosphate and calcium acetate agrees with studies that reported values up to 10 times higher after this surface modification [10,49]. These findings could be explained by the formation of the porous outer layer once the presence of pores in different layers creates uneven surfaces, increasing the roughness [50]. The nanometric scale of the changes caused by the tubes after the anodization with hydrofluoric acid did not cause roughness changes.

Regarding the SFE (Table 3 and Table 4), it has become apparent that CaP surfaces became more hydrophilic than other surfaces, showing the highest polar component. Higher SFE values after anodization were found previously due to the dissipation of the liquid inside the pores [51] and nanotubes [52]. However, careful comparisons must be made since SFE analyses after salivary pellicle formation in these surfaces are not found in the current literature. Furthermore, the increase in polar component values in salivary coated discs was also reported before [53] and indicates that the salivary pellicle film makes the titanium surfaces more hydrophilic. The changes on characteristics of dispersive and polar phases because of the salivary pellicle could have implications for microbial colonization. These findings highlight the importance of coating the dental titanium surfaces with pellicles before performing microbiological tests since it could equalize the modified surfaces [44,54,55], thereby interfering with potential activity to hinder microbial colonization.

Despite differences in topography, roughness, SFE, some statistical differences in viable counts (CFU/disc), in general, biofilm formation was quite similar for the same microorganism after surface treatments (Figure 3 and Figure 4). Regarding bacterial colonization, we focused on bacterial viability after adhesion and biofilm formation to assess whether the distinct surfaces would have antibiofilm and antibacterial effects, which was expected for the surfaces containing silver but was not confirmed here for all species tested. Next, the overall structure of the biofilms (that contains bacterial cells and the extracellular matrix) was evaluated via SEM. The images mainly showed bacterial cells, and only minimal extracellular material was observed for *P. gingivalis* on all surfaces in the culturing conditions used in the study (as depicted in Figure 3 and Figure 4). Nevertheless, the information of culturable cells and overall structure demonstrated the impact of the surfaces tested on adhesion and biofilm formation. Data on titanium surface roughness, wettability and bacterial adhesion/biofilm are ambiguous and difficult to compare due to differences such as the sterilization method [56,57] model conditions used in the microbiological tests [58], mainly short-term evaluations (24 or fewer hours) [39,42].

Bacterial adhesion on teeth and implants is considered quite similar. It can be summarized in three steps: (a) transport of the bacteria towards the substrate by saliva, (b) specific interactions between bacteria/substrate and between bacteria/ligand, and (c) bacterial aggregation [59]. There is a consensus that the physico-chemical properties of the surface influence this process. However, some authors have been suggested the surface roughness as the most important property [39,59,60,61], while others have reported the SFE as most important during biofilm formation [36,54,62]. Nevertheless, the importance of the used saliva-coated methods that turn the process more complex since salivary proteins (derived from the host and microorganisms) act as receptors, potentially masking the underlying surface characteristics of the material [53,54,55,61], is not always considered. It was suggested that the human saliva layer formed on the sample surface impeded the free interaction of bacteria with the sample surface leading to the absence of significant differences in bacterial adherence amount correlating with either roughness or SFE [44].

Therefore, some studies reported that more bacteria were found on moderately rough titanium surfaces than on smooth surfaces [42,60,63]. However, others demonstrated that rougher surfaces did not show an increase in adhesion and biofilm formation [58,64,65], similar to as in our findings, or even a reduction in CFU [66]. Based on the results shown here, the idea that the adhesion and formation of biofilm, either from initial colonizers or from periodontal pathogens, is facilitated on anodized surfaces with micropores and nanotubes due to increased roughness or changes in hydrophobicity could not be the only major factors. Thus, the highest rates of occurrence of progressive bone loss (PBL) around anodized surface implants [67] could be assigned to greater difficulty in removing the biofilm from surfaces with pores and/or interconnected tubes than with higher adhesion and higher amount of formed biofilm.

The destruction of the biofilm or the prevention of bacterial adhesion would be rational strategies to reduce the occurrence of PBL; however, to date, no treatment can guarantee these approaches [68]. Consequently, the functionalization of surfaces, such as the two-step anodization for the incorporation of silver ions, aimed to prevent bacterial adhesion and/or to kill those that reached the adhesion.

There is a lack of specific targeting for the production and evaluation of the performance of nanoparticles for dentistry [69], and the majority of good bactericidal effects were found in studies not aimed at oral biofilms and consequently with microorganisms that are not primary colonizers or periodontal pathogens [34,37] and/or without pre-coating the specimens with salivary pellicle [34,70]. EDS demonstrating the presence of silver oxide on CaPag and HFAg treated surfaces (Figure 2d,h; Supplementary Figures S2 and S3); however, for an effective strategy on medical and implant devices, the biomaterial surface needs to presents a null or highly reduced bacteria attachment to the surface [70], which was not achieved in this study. Furthermore, the amount and sizes of the nanoparticles here could have resulted in a deficient release of silver into the medium since silver nanoparticles must be able to release Ag+ to cause cell lysis due to the interaction with peptidoglycan cell wall and membranes, blocking of DNA replication and/or changes in protein synthesis [71]. Nevertheless, the salivary coating could have masked the potential antimicrobial effect of silver ions, as they could have complexed with the (glyco)proteins in the pellicle and had no adequate access to the bacterial surface. Thus, other ions with antimicrobial properties could be used in future studies using an anodization strategy to functionalize surfaces to improve metal implant device success in the oral cavity. The present findings are important because the oral cavity is bated by saliva and has hundreds of distinct species; hence, using some of the representative ones could be an indication to continue working with a material for downstream clinical applications or not, after weighting all possible advantages, including antiadhesion and antibiofilm traits.

## 5. Conclusions

The outcomes indicate that anodized and non-anodized Ti-35Nb-7Zr-5Ta exhibit affected microbial adhesion and subsequent biofilm formation of initial colonizers (*S. sanguinis*, *S. gordonii* and *A. naeslundii*) and a Gram-negative periodontal pathogen (*P. gingivalis*). Silver deposition did not hinder the colonization by these microorganisms, only decreasing *S. sanguinis* adhesion in both volcano-shaped and nanotubular surfaces, and biofilm formation on volcano-shaped surfaces of this alloy.

## Figures and Tables

**Table 1:** Experimental subgroups, electrolytes and anodizing parameters.

Subgroups	Electrolytes	Anodizing Parameters
Control	none	none
CaP	0.04 mol/L β-sodium glycerophosphate (Sigma Aldrich) and 0.35 mol/L calcium acetate (Sigma Aldrich)	1 min, 300 V, 2.5 amps
CaPAg	0.04 mol/l β-sodium glycerophosphate (Sigma Aldrich, St. Louis, MO, USA) and 0.35 mol/L calcium acetate (Sigma Aldrich)	1 min, 300 V, 2.5 amps
	0.01 M Silver nitrate (Sigma Aldrich)	2 min, 50 V, 2 amps
HF	0.3 M hydrofluoric acid (Sigma Aldrich)	60 min, 20 V, 2.5 amps
HFAg	0.3 M hydrofluoric acid (Sigma Aldrich)	60 min, 20 V, 2.5 amps
	0.01 M Silver nitrate (Sigma Aldrich)	2 min, 50 V, 2 amps

**Table 2:** Means and standard deviations of Ra, in µm.

	TAV	TNZT
Control	0.1914 (0.0137) ^Aa^	0.2762 (0.0592) ^Aa^
CaP	0.5114 (0.039) ^Ab^	0.5756 (0.1088) ^Abc^
CaPAg	0.525 (0.0333) ^Ab^	0.6488 (0.1811) ^Bc^
HF	0.1868 (0.0281) ^Aa^	0.3156 (0.0840) ^Ba^
HFAg	0.1885 (0.0237) ^Aa^	0.4848 (0.1864) ^Bb^

Different superscripted upper case letters indicate significant differences between the column titanium alloys (*p* < 0.05). Different superscripted lower letters indicate significant differences among rows—surface treatments (*p* < 0.05). Two-way ANOVA: alloys-F_1.45_ = 56.37; *p* < 0.0001; surface modifications-F_4.45_ = 53.43; *p* < 0.001; interactions-F_4.45_ = 4.862; *p* = 0.0024.

**Table 3:** Means (standard deviation), dispersive (γ^D^) and polar (γ^P^) components of SFE (in m/Nm) measured in discs without a salivary pellicle.

	SFE	TAV	TNZT
	γ^T^	47.51 (1.40) ^Aa^	47.23 (1.23) ^Aa^
Control	γ^D^	37.61	37.52
	γ^P^	9.90	9.71
	γ^T^	48.46 (3.29) ^Aab^	46.65 (0.69) ^Aa^
CaP	γ^D^	35.85	39.21
	γ^P^	12.61	7.35
	γ^T^	50.21 (1.13) ^Aab^	50.7 (2.07) ^Ab^
CaPAg	γ^D^	44.20	43.60
	γ^P^	6.01	7.16
	γ^T^	53.01 (4.41) ^Abc^	54.38 (1.79) ^Acd^
HF	γ^D^	38.37	41.72
	γ^P^	14.64	12.66
HFAg	γ^T^	50.69 (1.30) ^Ac^	54.59 (1.97) ^Ad^
	γ^D^	43.64	42.64
	γ^P^	7.05	11.84

Equal superscripted upper case letters indicate no significant differences between the columns-titanium alloys (*p* > 0.05). Different superscripted lower letters indicate significant differences among rows-surface treatments (*p* < 0.05). Two-way ANOVA: alloys-F_1.90_ = 2.645; *p* = 0.1074; surface modifications-F_4.90_ = 34.440; *p* < 0.0001; interactions-F_4.90_ = 4.76; *p* = 0.0016.

**Table 4:** Means (standard deviation), dispersive (γ^D^) and polar (γ^P^) components of SFE (in m/Nm) measured in discs with a salivary pellicle.

	SFE	TAV	TNZT
Control	γ^T^	45.44 (4.96) ^Aab^	46.65 (2.77) ^Aab^
	γ^D^	12.02	13.84
	γ^P^	33.43	32.81
CaP	γ^T^	56.98 (3.83) ^Ac^	60.51 (7.43) ^Ac^
	γ^D^	3.32	3.31
	γ^P^	52.76	57.12
CaPAg	γ^T^	50.17 (4.35) ^Abc^	51.97 (6.67) ^Abc^
	γ^D^	6.92	6.67
	γ^P^	43.25	45.30
HF	γ^T^	48.25 (6.06) ^Aab^	46.38 (3.70) ^Aab^
	γ^D^	7.08	12.57
	γ^P^	40.45	33.81
HFAg	γ^T^	45.91 (6.22) ^Aa^	40.80 (2.50) ^Aa^
	γ^D^	17.16	15.81
	γ^P^	27.75	24.99

Equal superscripted upper case letters indicate no significant differences between the columns-titanium alloys (*p* > 0.05). Different superscripted lower letters indicate significant differences among rows—surface treatments (*p* < 0.05). Two-way ANOVA: alloys-F_1.90_ = 0.4643; *p* = 0.4974; surface modifications-F_4.90_ = 30.12; *p* < 0.0001; interactions-F_4.90_ = 1.471; *p* = 0.2175.

## Data Availability

The data presented in this study are available as supplementary material. Additional data are available on request from the corresponding authors.

## Supplementary Materials

The following are available online at https://www.mdpi.com/article/10.3390/microorganisms9102154/s1. Figure S1: Schematic chart of the experimental design. Figure S2: Chemical mapping by EDS with elements concentration (wt%) for CaPAg TAV and HFAg TAV. Figure S3: Chemical mapping by EDS with elements concentration (wt%) for CaPAg TNZT and HFAg TNZT. Table S1: Statistical analysis of bacterial adhesion data. Table S2: Statistical analysis of bacterial biofilm data.
