# Supplementary material for: Microbial Adhesion and Biofilm Formation on Bioactive Surfaces of Ti-35Nb-7Zr-5Ta Alloy Created by Anodization
Source: Microorganisms. 2021 Oct 15;9(10):2154. doi: 10.3390/microorganisms9102154 (PMC8539148; doi:10.3390/microorganisms9102154)
Supplement: Supplementary file 1 [file microorganisms-09-02154-s001.zip › microorganisms-1392345 supplementary.pdf]

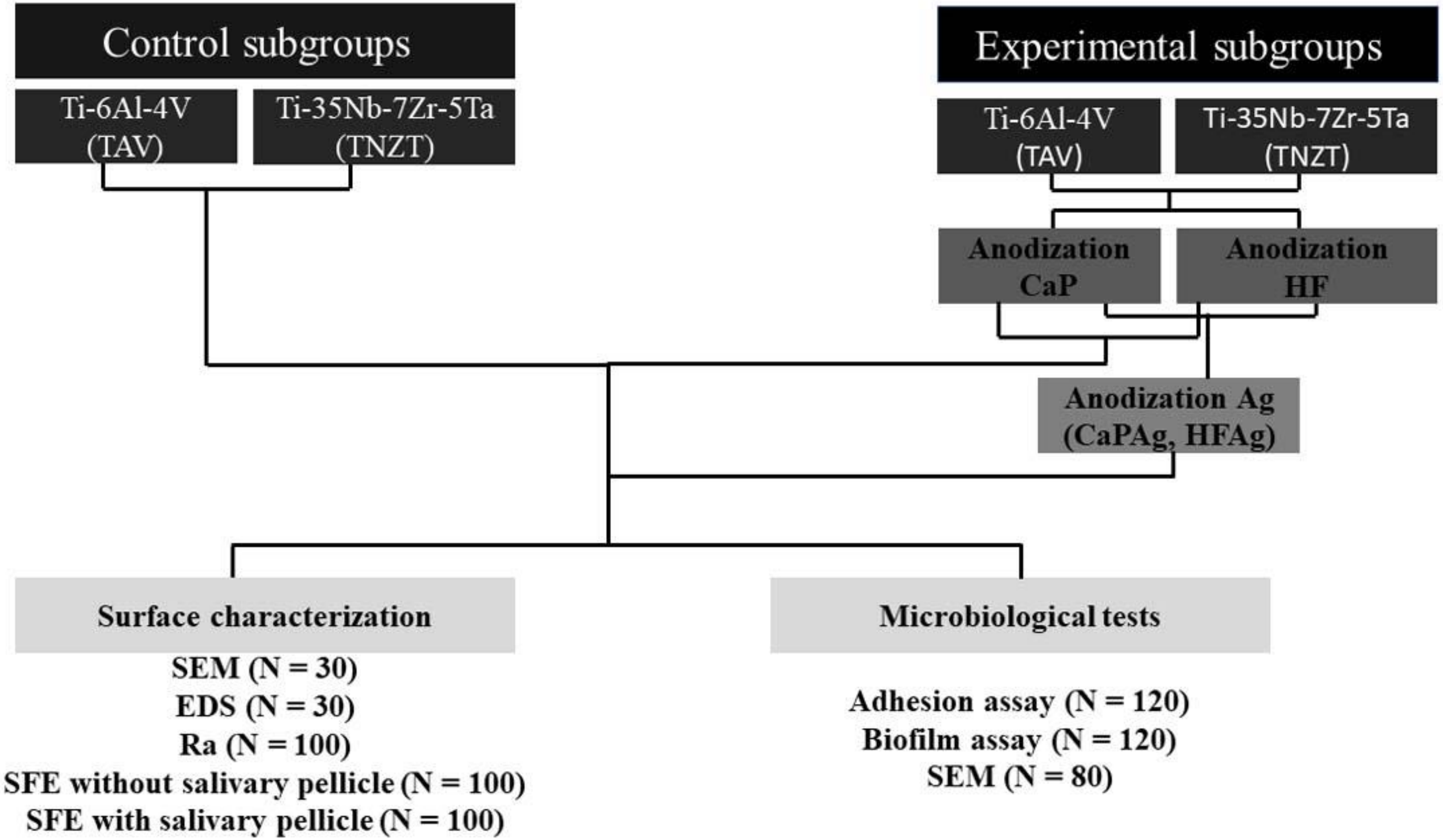

Figure S1 - Schematic chart of the experimental design.

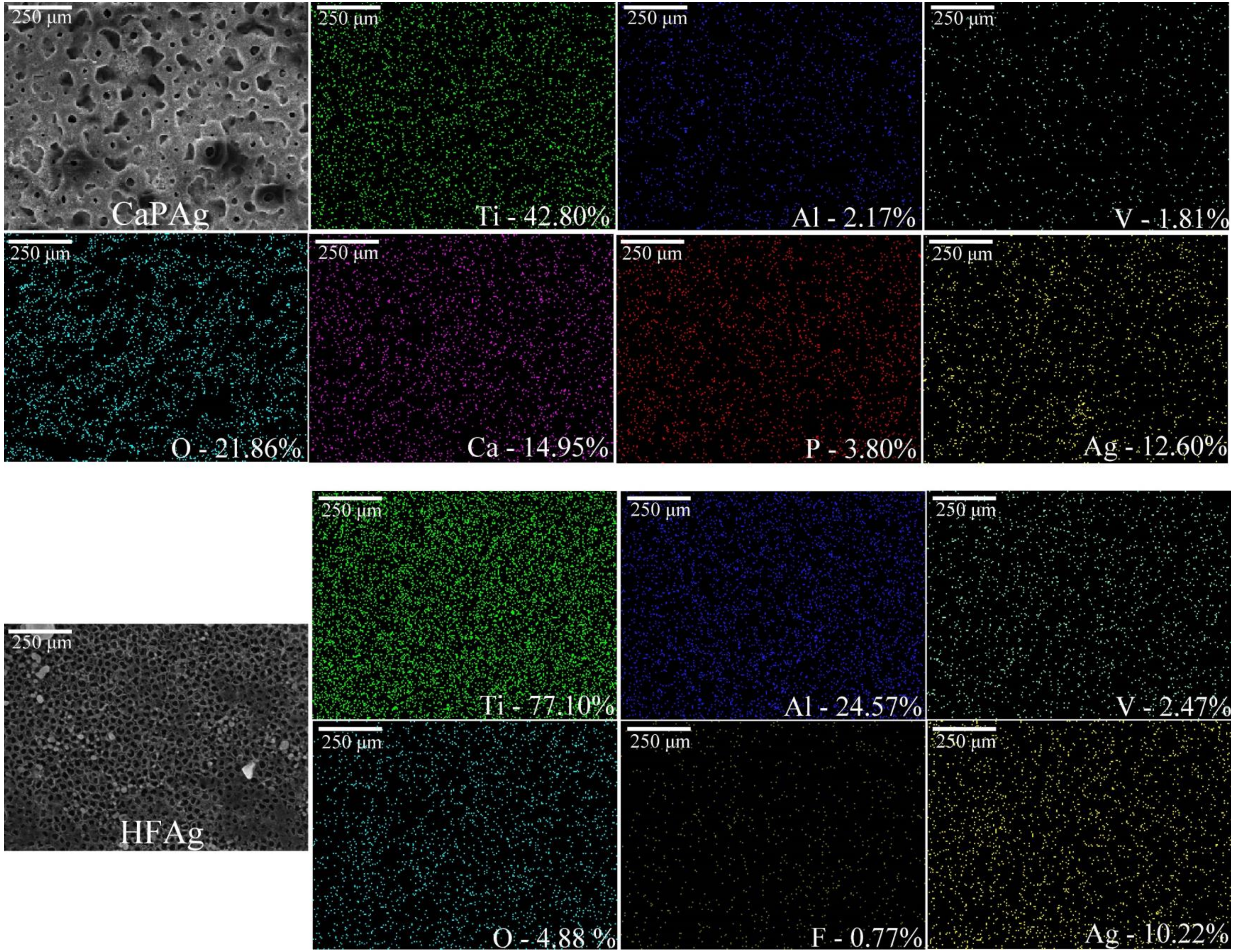

Figure S2 - Chemical mapping by EDS with elements concentration (wt%) for CaPAg TAV and HFAg TAV.

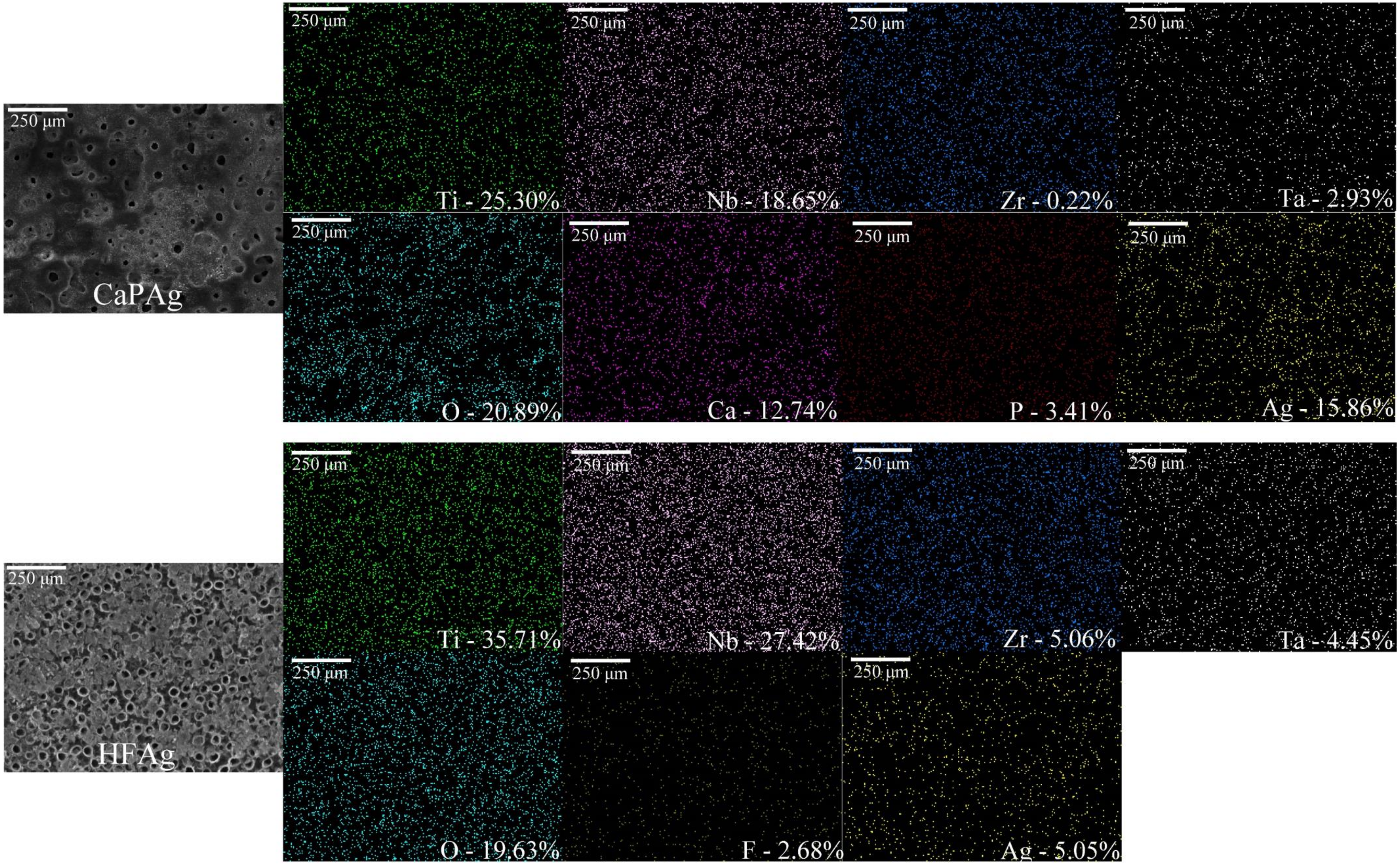

Figure S3 - Chemical mapping by EDS with elements concentration (wt%) for for CaPAg TNZT and HFAg TNZT.

Table S1. Statistical analysis of the bacterial adhesion data.

Table Analyzed	Grouped:				
Two-way ANOVA	Ordinary				
Alpha	0,05				
Source of Variation	% of total variation	P value	P value summary	Significant?	
Interaction	8,257	0,0011	**	Yes	
Species	53,51	<0,0001	****	Yes	
Surface	11,71	<0,0001	****	Yes	
ANOVA table	SS (Type III)	DF	MS	F (DFn, DFd)	P value
Interaction	11,03	27	0,4086	F (27, 194) = 2,206	P=0,0011
Species	71,51	3	23,84	F (3, 194) = 128,7	P<0,0001
Surface	15,64	9	1,738	F (9, 194) = 9,385	P<0,0001
Residual	35,93	194	0,1852		
Data summary					
Number of columns (Surface)	10				
Number of rows (Species)	4				
Number of values	234				

Multiple comparisons

Compare cell means regardless of rows and columns					
Number of families	1				
Number of comparisons per family	780				
Alpha	0,05				
Tukey's multiple comparisons test	Predicted (LS) mean diff,	95,00% CI of diff,	Below threshold?	Summary	Adjusted P Value
S. gordonii:TAV - Control vs. *S. gordonii*:TAV - CaP	-0,5483	-1,530 to 0,4334	No	ns	0,9734
S. gordonii:TAV - Control vs. *S. gordonii*:TAV - CaPAg	-0,1867	-1,168 to 0,7951	No	ns	>0,9999
S. gordonii:TAV - Control vs. *S. gordonii*:TAV - HF	-0,1660	-1,196 to 0,8637	No	ns	>0,9999
S. gordonii:TAV - Control vs. *S. gordonii*:TAV - HFAg	0,1333	-0,8484 to 1,115	No	ns	>0,9999
S. gordonii:TAV - Control vs. *S. gordonii*:TNZT - Control	-0,1217	-1,103 to 0,8601	No	ns	>0,9999
S. gordonii:TAV - Control vs. *S. gordonii*:TNZT - CaP	-0,6617	-1,643 to 0,3201	No	ns	0,7857
S. gordonii:TAV - Control vs. *S. gordonii*:TNZT - CaPAg	-0,2317	-1,213 to 0,7501	No	ns	>0,9999
S. gordonii:TAV - Control vs. *S. gordonii*:TNZT - HF	0,1383	-0,8434 to 1,120	No	ns	>0,9999
S. gordonii:TAV - Control vs. *S. gordonii*:TNZT - HFAg	0,04333	-0,9384 to 1,025	No	ns	>0,9999
S. gordonii:TAV - Control vs. *S. sanguinis*:TAV - Control	0,3517	-0,6301 to 1,333	No	ns	>0,9999
S. gordonii:TAV - Control vs. *S. sanguinis*:TAV - CaP	-0,04000	-1,022 to 0,9417	No	ns	>0,9999
S. gordonii:TAV - Control vs. *S. sanguinis*:TAV - CaPAg	0,1367	-0,8451 to 1,118	No	ns	>0,9999
S. gordonii:TAV - Control vs. *S. sanguinis*:TAV - HF	0,005000	-0,9767 to 0,9867	No	ns	>0,9999
S. gordonii:TAV - Control vs. *S. sanguinis*:TAV - HFAg	0,3000	-0,6817 to 1,282	No	ns	>0,9999
S. gordonii:TAV - Control vs. *S. sanguinis*:TNZT - Control	-0,2350	-1,217 to 0,7467	No	ns	>0,9999
S. gordonii:TAV - Control vs. *S. sanguinis*:TNZT - CaP	-0,4117	-1,393 to 0,5701	No	ns	0,9998
S. gordonii:TAV - Control vs. *S. sanguinis*:TNZT - CaPAg	0,1467	-0,8351 to 1,128	No	ns	>0,9999
S. gordonii:TAV - Control vs. *S. sanguinis*:TNZT - HF	0,1367	-0,8451 to 1,118	No	ns	>0,9999
S. gordonii:TAV - Control vs. *S. sanguinis*:TNZT - HFAg	0,6900	-0,2917 to 1,672	No	ns	0,7035
S. gordonii:TAV - Control vs. *A. naeslundii*:TAV - Control	-0,3350	-1,317 to 0,6467	No	ns	>0,9999
S. gordonii:TAV - Control vs. *A. naeslundii*:TAV - CaP	-0,7300	-1,712 to 0,2517	No	ns	0,5747
S. gordonii:TAV - Control vs. *A. naeslundii*:TAV - CaPAg	-0,8767	-1,858 to 0,1051	No	ns	0,1732
S. gordonii:TAV - Control vs. *A. naeslundii*:TAV - HF	-0,1660	-1,196 to 0,8637	No	ns	>0,9999
S. gordonii:TAV - Control vs. *A. naeslundii*:TAV - HFAg	0,04333	-0,9384 to 1,025	No	ns	>0,9999
S. gordonii:TAV - Control vs. *A. naeslundii*:TNZT - Control	-0,8617	-1,843 to 0,1201	No	ns	0,2019
***S. gordonii*:TAV - Control vs. *A. naeslundii*:TNZT - CaP**	**-1,032**	**-2,013 to -0,04992**	**Yes**	*****	**0,0253**
S. gordonii:TAV - Control vs. *A. naeslundii*:TNZT - CaPAg	-0,7267	-1,708 to 0,2551	No	ns	0,5857
S. gordonii:TAV - Control vs. *A. naeslundii*:TNZT - HF	-0,2167	-1,198 to 0,7651	No	ns	>0,9999
S. gordonii:TAV - Control vs. *A. naeslundii*:TNZT - HFAg	-0,5560	-1,586 to 0,4737	No	ns	0,9836
S. gordonii:TAV - Control vs. *P. gingivalis*:TAV - Control	-0,7067	-1,688 to 0,2751	No	ns	0,6510
***S. gordonii*:TAV - Control vs. *P. gingivalis*:TAV - CaP**	**-1,345**	**-2,327 to -0,3633**	**Yes**	*******	**0,0001**
***S. gordonii*:TAV - Control vs. *P. gingivalis*:TAV - CaPAg**	**-1,742**	**-2,723 to -0,7599**	**Yes**	********	**<0,0001**
***S. gordonii*:TAV - Control vs. *P. gingivalis*:TAV - HF**	**-1,077**	**-2,058 to -0,09492**	**Yes**	*****	**0,0131**
***S. gordonii*:TAV - Control vs. *P. gingivalis*:TAV - HFAg**	**-1,062**	**-2,043 to -0,07992**	**Yes**	*****	**0,0164**
S. gordonii:TAV - Control vs. *P. gingivalis*:TNZT - Control	-0,8160	-1,846 to 0,2137	No	ns	0,4191
***S. gordonii*:TAV - Control vs. *P. gingivalis*:TNZT - CaP**	**-2,015**	**-3,113 to -0,9174**	**Yes**	********	**<0,0001**
***S. gordonii*:TAV - Control vs. *P. gingivalis*:TNZT - CaPAg**	**-1,998**	**-2,980 to -1,017**	**Yes**	********	**<0,0001**
***S. gordonii*:TAV - Control vs. *P. gingivalis*:TNZT - HF**	**-1,253**	**-2,235 to -0,2716**	**Yes**	*******	**0,0007**
***S. gordonii*:TAV - Control vs. *P. gingivalis*:TNZT - HFAg**	**-1,693**	**-2,675 to -0,7116**	**Yes**	********	**<0,0001**
S. gordonii:TAV - CaP vs. *S. gordonii*:TAV - CaPAg	0,3617	-0,6201 to 1,343	No	ns	>0,9999
S. gordonii:TAV - CaP vs. *S. gordonii*:TAV - HF	0,3823	-0,6473 to 1,412	No	ns	>0,9999

S. gordonii:TAV - CaP vs. *S. gordonii*:TAV - HFAg		0,6817	-0,3001 to 1,663	No	ns	0,7288
S. gordonii:TAV - CaP vs. *S. gordonii*:TNZT - Control		0,4267	-0,5551 to 1,408	No	ns	0,9997
S. gordonii:TAV - CaP vs. *S. gordonii*:TNZT - CaP		-0,1133	-1,095 to 0,8684	No	ns	>0,9999
S. gordonii:TAV - CaP vs. *S. gordonii*:TNZT - CaPAg		0,3167	-0,6651 to 1,298	No	ns	>0,9999
S. gordonii:TAV - CaP vs. *S. gordonii*:TNZT - HF		0,6867	-0,2951 to 1,668	No	ns	0,7137
S. gordonii:TAV - CaP vs. *S. gordonii*:TNZT - HFAg		0,5917	-0,3901 to 1,573	No	ns	0,9297
S. gordonii:TAV - CaP vs. *S. sanguinis*:TAV - Control		0,9000	-0,08174 to 1,882	No	ns	0,1347
S. gordonii:TAV - CaP vs. *S. sanguinis*:TAV - CaP		0,5083	-0,4734 to 1,490	No	ns	0,9915
S. gordonii:TAV - CaP vs. *S. sanguinis*:TAV - CaPAg		0,6850	-0,2967 to 1,667	No	ns	0,7188
S. gordonii:TAV - CaP vs. *S. sanguinis*:TAV - HF		0,5533	-0,4284 to 1,535	No	ns	0,9698
S. gordonii:TAV - CaP vs. *S. sanguinis*:TAV - HFAg		0,8483	-0,1334 to 1,830	No	ns	0,2301
S. gordonii:TAV - CaP vs. *S. sanguinis*:TNZT - Control		0,3133	-0,6684 to 1,295	No	ns	>0,9999
S. gordonii:TAV - CaP vs. *S. sanguinis*:TNZT - CaP		0,1367	-0,8451 to 1,118	No	ns	>0,9999
S. gordonii:TAV - CaP vs. *S. sanguinis*:TNZT - CaPAg		0,6950	-0,2867 to 1,677	No	ns	0,6880
S. gordonii:TAV - CaP vs. *S. sanguinis*:TNZT - HF		0,6850	-0,2967 to 1,667	No	ns	0,7188
***S. gordonii*:TAV - CaP vs. *S. sanguinis*:TNZT - HFAg**		**1,238**	**0,2566 to 2,220**	**Yes**	*******	**0,0009**
S. gordonii:TAV - CaP vs. *A. naeslundii*:TAV - Control		0,2133	-0,7684 to 1,195	No	ns	>0,9999
S. gordonii:TAV - CaP vs. *A. naeslundii*:TAV - CaP		-0,1817	-1,163 to 0,8001	No	ns	>0,9999
S. gordonii:TAV - CaP vs. *A. naeslundii*:TAV - CaPAg		-0,3283	-1,310 to 0,6534	No	ns	>0,9999
S. gordonii:TAV - CaP vs. *A. naeslundii*:TAV - HF		0,3823	-0,6473 to 1,412	No	ns	>0,9999
S. gordonii:TAV - CaP vs. *A. naeslundii*:TAV - HFAg		0,5917	-0,3901 to 1,573	No	ns	0,9297
S. gordonii:TAV - CaP vs. *A. naeslundii*:TNZT - Control		-0,3133	-1,295 to 0,6684	No	ns	>0,9999
S. gordonii:TAV - CaP vs. *A. naeslundii*:TNZT - CaP		-0,4833	-1,465 to 0,4984	No	ns	0,9964
S. gordonii:TAV - CaP vs. *A. naeslundii*:TNZT - CaPAg		-0,1783	-1,160 to 0,8034	No	ns	>0,9999
S. gordonii:TAV - CaP vs. *A. naeslundii*:TNZT - HF		0,3317	-0,6501 to 1,313	No	ns	>0,9999
S. gordonii:TAV - CaP vs. *A. naeslundii*:TNZT - HFAg		-0,007667	-1,037 to 1,022	No	ns	>0,9999
S. gordonii:TAV - CaP vs. *P. gingivalis*:TAV - Control		-0,1583	-1,140 to 0,8234	No	ns	>0,9999
S. gordonii:TAV - CaP vs. *P. gingivalis*:TAV - CaP		-0,7967	-1,778 to 0,1851	No	ns	0,3631
***S. gordonii*:TAV - CaP vs. *P. gingivalis*:TAV - CaPAg**		**-1,193**	**-2,175 to -0,2116**	**Yes**	******	**0,0020**
S. gordonii:TAV - CaP vs. *P. gingivalis*:TAV - HF		-0,5283	-1,510 to 0,4534	No	ns	0,9844
S. gordonii:TAV - CaP vs. *P. gingivalis*:TAV - HFAg		-0,5133	-1,495 to 0,4684	No	ns	0,9900
S. gordonii:TAV - CaP vs. *P. gingivalis*:TNZT - Control		-0,2677	-1,297 to 0,7620	No	ns	>0,9999
***S. gordonii*:TAV - CaP vs. *P. gingivalis*:TNZT - CaP**		**-1,467**	**-2,564 to -0,3690**	**Yes**	*******	**0,0002**
***S. gordonii*:TAV - CaP vs. *P. gingivalis*:TNZT - CaPAg**		**-1,450**	**-2,432 to -0,4683**	**Yes**	********	**<0,0001**
S. gordonii:TAV - CaP vs. *P. gingivalis*:TNZT - HF		-0,7050	-1,687 to 0,2767	No	ns	0,6563
***S. gordonii*:TAV - CaP vs. *P. gingivalis*:TNZT - HFAg**		**-1,145**	**-2,127 to -0,1633**	**Yes**	******	**0,0045**
S. gordonii:TAV - CaPAg vs. *S. gordonii*:TAV - HF		0,02067	-1,009 to 1,050	No	ns	>0,9999
S. gordonii:TAV - CaPAg vs. *S. gordonii*:TAV - HFAg		0,3200	-0,6617 to 1,302	No	ns	>0,9999
S. gordonii:TAV - CaPAg vs. *S. gordonii*:TNZT - Control		0,06500	-0,9167 to 1,047	No	ns	>0,9999
S. gordonii:TAV - CaPAg vs. *S. gordonii*:TNZT - CaP		-0,4750	-1,457 to 0,5067	No	ns	0,9973
S. gordonii:TAV - CaPAg vs. *S. gordonii*:TNZT - CaPAg		-0,04500	-1,027 to 0,9367	No	ns	>0,9999
S. gordonii:TAV - CaPAg vs. *S. gordonii*:TNZT - HF		0,3250	-0,6567 to 1,307	No	ns	>0,9999
S. gordonii:TAV - CaPAg vs. *S. gordonii*:TNZT - HFAg		0,2300	-0,7517 to 1,212	No	ns	>0,9999
S. gordonii:TAV - CaPAg vs. *S. sanguinis*:TAV - Control		0,5383	-0,4434 to 1,520	No	ns	0,9795
S. gordonii:TAV - CaPAg vs. *S. sanguinis*:TAV - CaP		0,1467	-0,8351 to 1,128	No	ns	>0,9999
S. gordonii:TAV - CaPAg vs. *S. sanguinis*:TAV - CaPAg		0,3233	-0,6584 to 1,305	No	ns	>0,9999
S. gordonii:TAV - CaPAg vs. *S. sanguinis*:TAV - HF		0,1917	-0,7901 to 1,173	No	ns	>0,9999
S. gordonii:TAV - CaPAg vs. *S. sanguinis*:TAV - HFAg		0,4867	-0,4951 to 1,468	No	ns	0,9959
S. gordonii:TAV - CaPAg vs. *S. sanguinis*:TNZT - Control		-0,04833	-1,030 to 0,9334	No	ns	>0,9999
S. gordonii:TAV - CaPAg vs. *S. sanguinis*:TNZT - CaP		-0,2250	-1,207 to 0,7567	No	ns	>0,9999
S. gordonii:TAV - CaPAg vs. *S. sanguinis*:TNZT - CaPAg		0,3333	-0,6484 to 1,315	No	ns	>0,9999
S. gordonii:TAV - CaPAg vs. *S. sanguinis*:TNZT - HF		0,3233	-0,6584 to 1,305	No	ns	>0,9999
S. gordonii:TAV - CaPAg vs. *S. sanguinis*:TNZT - HFAg		0,8767	-0,1051 to 1,858	No	ns	0,1732
S. gordonii:TAV - CaPAg vs. *A. naeslundii*:TAV - Control		-0,1483	-1,130 to 0,8334	No	ns	>0,9999
S. gordonii:TAV - CaPAg vs. *A. naeslundii*:TAV - CaP		-0,5433	-1,525 to 0,4384	No	ns	0,9766
S. gordonii:TAV - CaPAg vs. *A. naeslundii*:TAV - CaPAg		-0,6900	-1,672 to 0,2917	No	ns	0,7035
S. gordonii:TAV - CaPAg vs. *A. naeslundii*:TAV - HF		0,02067	-1,009 to 1,050	No	ns	>0,9999
S. gordonii:TAV - CaPAg vs. *A. naeslundii*:TAV - HFAg		0,2300	-0,7517 to 1,212	No	ns	>0,9999
S. gordonii:TAV - CaPAg vs. *A. naeslundii*:TNZT - Control		-0,6750	-1,657 to 0,3067	No	ns	0,7484
S. gordonii:TAV - CaPAg vs. *A. naeslundii*:TNZT - CaP		-0,8450	-1,827 to 0,1367	No	ns	0,2376
S. gordonii:TAV - CaPAg vs. *A. naeslundii*:TNZT - CaPAg		-0,5400	-1,522 to 0,4417	No	ns	0,9786
S. gordonii:TAV - CaPAg vs. *A. naeslundii*:TNZT - HF		-0,03000	-1,012 to 0,9517	No	ns	>0,9999
S. gordonii:TAV - CaPAg vs. *A. naeslundii*:TNZT - HFAg		-0,3693	-1,399 to 0,6603	No	ns	>0,9999
S. gordonii:TAV - CaPAg vs. *P. gingivalis*:TAV - Control		-0,5200	-1,502 to 0,4617	No	ns	0,9878
***S. gordonii*:TAV - CaPAg vs. *P. gingivalis*:TAV - CaP**		**-1,158**	**-2,140 to -0,1766**	**Yes**	******	**0,0036**
***S. gordonii*:TAV - CaPAg vs. *P. gingivalis*:TAV - CaPAg**		**-1,555**	**-2,537 to -0,5733**	**Yes**	********	**<0,0001**
S. gordonii:TAV - CaPAg vs. *P. gingivalis*:TAV - HF		-0,8900	-1,872 to 0,09174	No	ns	0,1503
S. gordonii:TAV - CaPAg vs. *P. gingivalis*:TAV - HFAg		-0,8750	-1,857 to 0,1067	No	ns	0,1762
S. gordonii:TAV - CaPAg vs. *P. gingivalis*:TNZT - Control		-0,6293	-1,659 to 0,4003	No	ns	0,9174
***S. gordonii*:TAV - CaPAg vs. *P. gingivalis*:TNZT - CaP**		**-1,828**	**-2,926 to -0,7307**	**Yes**	********	**<0,0001**
***S. gordonii*:TAV - CaPAg vs. *P. gingivalis*:TNZT - CaPAg**		**-1,812**	**-2,793 to -0,8299**	**Yes**	********	**<0,0001**
***S. gordonii*:TAV - CaPAg vs. *P. gingivalis*:TNZT - HF**		**-1,067**	**-2,048 to -0,08492**	**Yes**	*****	**0,0152**

***S. gordonii*:TAV - CaPAg vs. *P. gingivalis*:TNZT - HFAg**	**-1,507**	**-2,488 to -0,5249**	**Yes**	****	**<0,0001**
S. gordonii:TAV - HF vs. *S. gordonii*:TAV - HFAg	0,2993	-0,7303 to 1,329	No	ns	>0,9999
S. gordonii:TAV - HF vs. *S. gordonii*:TNZT - Control	0,04433	-0,9853 to 1,074	No	ns	>0,9999
S. gordonii:TAV - HF vs. *S. gordonii*:TNZT - CaP	-0,4957	-1,525 to 0,5340	No	ns	0,9976
S. gordonii:TAV - HF vs. *S. gordonii*:TNZT - CaPAg	-0,06567	-1,095 to 0,9640	No	ns	>0,9999
S. gordonii:TAV - HF vs. *S. gordonii*:TNZT - HF	0,3043	-0,7253 to 1,334	No	ns	>0,9999
S. gordonii:TAV - HF vs. *S. gordonii*:TNZT - HFAg	0,2093	-0,8203 to 1,239	No	ns	>0,9999
S. gordonii:TAV - HF vs. *S. sanguinis*:TAV - Control	0,5177	-0,5120 to 1,547	No	ns	0,9948
S. gordonii:TAV - HF vs. *S. sanguinis*:TAV - CaP	0,1260	-0,9037 to 1,156	No	ns	>0,9999
S. gordonii:TAV - HF vs. *S. sanguinis*:TAV - CaPAg	0,3027	-0,7270 to 1,332	No	ns	>0,9999
S. gordonii:TAV - HF vs. *S. sanguinis*:TAV - HF	0,1710	-0,8587 to 1,201	No	ns	>0,9999
S. gordonii:TAV - HF vs. *S. sanguinis*:TAV - HFAg	0,4660	-0,5637 to 1,496	No	ns	0,9993
S. gordonii:TAV - HF vs. *S. sanguinis*:TNZT - Control	-0,06900	-1,099 to 0,9607	No	ns	>0,9999
S. gordonii:TAV - HF vs. *S. sanguinis*:TNZT - CaP	-0,2457	-1,275 to 0,7840	No	ns	>0,9999
S. gordonii:TAV - HF vs. *S. sanguinis*:TNZT - CaPAg	0,3127	-0,7170 to 1,342	No	ns	>0,9999
S. gordonii:TAV - HF vs. *S. sanguinis*:TNZT - HF	0,3027	-0,7270 to 1,332	No	ns	>0,9999
S. gordonii:TAV - HF vs. *S. sanguinis*:TNZT - HFAg	0,8560	-0,1737 to 1,886	No	ns	0,3087
S. gordonii:TAV - HF vs. *A. naeslundii*:TAV - Control	-0,1690	-1,199 to 0,8607	No	ns	>0,9999
S. gordonii:TAV - HF vs. *A. naeslundii*:TAV - CaP	-0,5640	-1,594 to 0,4657	No	ns	0,9798
S. gordonii:TAV - HF vs. *A. naeslundii*:TAV - CaPAg	-0,7107	-1,740 to 0,3190	No	ns	0,7408
S. gordonii:TAV - HF vs. *A. naeslundii*:TAV - HF	0,000	-1,075 to 1,075	No	ns	>0,9999
S. gordonii:TAV - HF vs. *A. naeslundii*:TAV - HFAg	0,2093	-0,8203 to 1,239	No	ns	>0,9999
S. gordonii:TAV - HF vs. *A. naeslundii*:TNZT - Control	-0,6957	-1,725 to 0,3340	No	ns	0,7813
S. gordonii:TAV - HF vs. *A. naeslundii*:TNZT - CaP	-0,8657	-1,895 to 0,1640	No	ns	0,2847
S. gordonii:TAV - HF vs. *A. naeslundii*:TNZT - CaPAg	-0,5607	-1,590 to 0,4690	No	ns	0,9815
S. gordonii:TAV - HF vs. *A. naeslundii*:TNZT - HF	-0,05067	-1,080 to 0,9790	No	ns	>0,9999
S. gordonii:TAV - HF vs. *A. naeslundii*:TNZT - HFAg	-0,3900	-1,465 to 0,6854	No	ns	>0,9999
S. gordonii:TAV - HF vs. *P. gingivalis*:TAV - Control	-0,5407	-1,570 to 0,4890	No	ns	0,9893
***S. gordonii*:TAV - HF vs. *P. gingivalis*:TAV - CaP**	**-1,179**	**-2,209 to -0,1493**	**Yes**	**	**0,0063**
***S. gordonii*:TAV - HF vs. *P. gingivalis*:TAV - CaPAg**	**-1,576**	**-2,605 to -0,5460**	**Yes**	****	**<0,0001**
S. gordonii:TAV - HF vs. *P. gingivalis*:TAV - HF	-0,9107	-1,940 to 0,1190	No	ns	0,1888
S. gordonii:TAV - HF vs. *P. gingivalis*:TAV - HFAg	-0,8957	-1,925 to 0,1340	No	ns	0,2179
S. gordonii:TAV - HF vs. *P. gingivalis*:TNZT - Control	-0,6500	-1,725 to 0,4254	No	ns	0,9273
***S. gordonii*:TAV - HF vs. *P. gingivalis*:TNZT - CaP**	**-1,849**	**-2,990 to -0,7083**	**Yes**	****	**<0,0001**
***S. gordonii*:TAV - HF vs. *P. gingivalis*:TNZT - CaPAg**	**-1,832**	**-2,862 to -0,8027**	**Yes**	****	**<0,0001**
***S. gordonii*:TAV - HF vs. *P. gingivalis*:TNZT - HF**	**-1,087**	**-2,117 to -0,05767**	**Yes**	*	**0,0235**
***S. gordonii*:TAV - HF vs. *P. gingivalis*:TNZT - HFAg**	**-1,527**	**-2,557 to -0,4977**	**Yes**	****	**<0,0001**
S. gordonii:TAV - HFAg vs. *S. gordonii*:TNZT - Control	-0,2550	-1,237 to 0,7267	No	ns	>0,9999
S. gordonii:TAV - HFAg vs. *S. gordonii*:TNZT - CaP	-0,7950	-1,777 to 0,1867	No	ns	0,3679
S. gordonii:TAV - HFAg vs. *S. gordonii*:TNZT - CaPAg	-0,3650	-1,347 to 0,6167	No	ns	>0,9999
S. gordonii:TAV - HFAg vs. *S. gordonii*:TNZT - HF	0,005000	-0,9767 to 0,9867	No	ns	>0,9999
S. gordonii:TAV - HFAg vs. *S. gordonii*:TNZT - HFAg	-0,09000	-1,072 to 0,8917	No	ns	>0,9999
S. gordonii:TAV - HFAg vs. *S. sanguinis*:TAV - Control	0,2183	-0,7634 to 1,200	No	ns	>0,9999
S. gordonii:TAV - HFAg vs. *S. sanguinis*:TAV - CaP	-0,1733	-1,155 to 0,8084	No	ns	>0,9999
S. gordonii:TAV - HFAg vs. *S. sanguinis*:TAV - CaPAg	0,003333	-0,9784 to 0,9851	No	ns	>0,9999
S. gordonii:TAV - HFAg vs. *S. sanguinis*:TAV - HF	-0,1283	-1,110 to 0,8534	No	ns	>0,9999
S. gordonii:TAV - HFAg vs. *S. sanguinis*:TAV - HFAg	0,1667	-0,8151 to 1,148	No	ns	>0,9999
S. gordonii:TAV - HFAg vs. *S. sanguinis*:TNZT - Control	-0,3683	-1,350 to 0,6134	No	ns	>0,9999
S. gordonii:TAV - HFAg vs. *S. sanguinis*:TNZT - CaP	-0,5450	-1,527 to 0,4367	No	ns	0,9756
S. gordonii:TAV - HFAg vs. *S. sanguinis*:TNZT - CaPAg	0,01333	-0,9684 to 0,9951	No	ns	>0,9999
S. gordonii:TAV - HFAg vs. *S. sanguinis*:TNZT - HF	0,003333	-0,9784 to 0,9851	No	ns	>0,9999
S. gordonii:TAV - HFAg vs. *S. sanguinis*:TNZT - HFAg	0,5567	-0,4251 to 1,538	No	ns	0,9673
S. gordonii:TAV - HFAg vs. *A. naeslundii*:TAV - Control	-0,4683	-1,450 to 0,5134	No	ns	0,9979
S. gordonii:TAV - HFAg vs. *A. naeslundii*:TAV - CaP	-0,8633	-1,845 to 0,1184	No	ns	0,1986
***S. gordonii*:TAV - HFAg vs. *A. naeslundii*:TAV - CaPAg**	**-1,010**	**-1,992 to -0,02826**	**Yes**	*	**0,0342**
S. gordonii:TAV - HFAg vs. *A. naeslundii*:TAV - HF	-0,2993	-1,329 to 0,7303	No	ns	>0,9999
S. gordonii:TAV - HFAg vs. *A. naeslundii*:TAV - HFAg	-0,09000	-1,072 to 0,8917	No	ns	>0,9999
***S. gordonii*:TAV - HFAg vs. *A. naeslundii*:TNZT - Control**	**-0,9950**	**-1,977 to -0,01326**	**Yes**	*	**0,0419**
***S. gordonii*:TAV - HFAg vs. *A. naeslundii*:TNZT - CaP**	**-1,165**	**-2,147 to -0,1833**	**Yes**	**	**0,0032**
S. gordonii:TAV - HFAg vs. *A. naeslundii*:TNZT - CaPAg	-0,8600	-1,842 to 0,1217	No	ns	0,2053
S. gordonii:TAV - HFAg vs. *A. naeslundii*:TNZT - HF	-0,3500	-1,332 to 0,6317	No	ns	>0,9999
S. gordonii:TAV - HFAg vs. *A. naeslundii*:TNZT - HFAg	-0,6893	-1,719 to 0,3403	No	ns	0,7975
S. gordonii:TAV - HFAg vs. *P. gingivalis*:TAV - Control	-0,8400	-1,822 to 0,1417	No	ns	0,2491
***S. gordonii*:TAV - HFAg vs. *P. gingivalis*:TAV - CaP**	**-1,478**	**-2,460 to -0,4966**	**Yes**	****	**<0,0001**
***S. gordonii*:TAV - HFAg vs. *P. gingivalis*:TAV - CaPAg**	**-1,875**	**-2,857 to -0,8933**	**Yes**	****	**<0,0001**
***S. gordonii*:TAV - HFAg vs. *P. gingivalis*:TAV - HF**	**-1,210**	**-2,192 to -0,2283**	**Yes**	**	**0,0015**
***S. gordonii*:TAV - HFAg vs. *P. gingivalis*:TAV - HFAg**	**-1,195**	**-2,177 to -0,2133**	**Yes**	**	**0,0020**
S. gordonii:TAV - HFAg vs. *P. gingivalis*:TNZT - Control	-0,9493	-1,979 to 0,08033	No	ns	0,1272
***S. gordonii*:TAV - HFAg vs. *P. gingivalis*:TNZT - CaP**	**-2,148**	**-3,246 to -1,051**	**Yes**	****	**<0,0001**
***S. gordonii*:TAV - HFAg vs. *P. gingivalis*:TNZT - CaPAg**	**-2,132**	**-3,113 to -1,150**	**Yes**	****	**<0,0001**
***S. gordonii*:TAV - HFAg vs. *P. gingivalis*:TNZT - HF**	**-1,387**	**-2,368 to -0,4049**	**Yes**	****	**<0,0001**
***S. gordonii*:TAV - HFAg vs. *P. gingivalis*:TNZT - HFAg**	**-1,827**	**-2,808 to -0,8449**	**Yes**	****	**<0,0001**

S. gordonii:TNZT - Control vs. *S. gordonii*:TNZT - CaP	-0,5400	-1,522 to 0,4417	No	ns	0,9786
S. gordonii:TNZT - Control vs. *S. gordonii*:TNZT - CaPAg	-0,1100	-1,092 to 0,8717	No	ns	>0,9999
S. gordonii:TNZT - Control vs. *S. gordonii*:TNZT - HF	0,2600	-0,7217 to 1,242	No	ns	>0,9999
S. gordonii:TNZT - Control vs. *S. gordonii*:TNZT - HFAg	0,1650	-0,8167 to 1,147	No	ns	>0,9999
S. gordonii:TNZT - Control vs. *S. sanguinis*:TAV - Control	0,4733	-0,5084 to 1,455	No	ns	0,9975
S. gordonii:TNZT - Control vs. *S. sanguinis*:TAV - CaP	0,08167	-0,9001 to 1,063	No	ns	>0,9999
S. gordonii:TNZT - Control vs. *S. sanguinis*:TAV - CaPAg	0,2583	-0,7234 to 1,240	No	ns	>0,9999
S. gordonii:TNZT - Control vs. *S. sanguinis*:TAV - HF	0,1267	-0,8551 to 1,108	No	ns	>0,9999
S. gordonii:TNZT - Control vs. *S. sanguinis*:TAV - HFAg	0,4217	-0,5601 to 1,403	No	ns	0,9997
S. gordonii:TNZT - Control vs. *S. sanguinis*:TNZT - Control	-0,1133	-1,095 to 0,8684	No	ns	>0,9999
S. gordonii:TNZT - Control vs. *S. sanguinis*:TNZT - CaP	-0,2900	-1,272 to 0,6917	No	ns	>0,9999
S. gordonii:TNZT - Control vs. *S. sanguinis*:TNZT - CaPAg	0,2683	-0,7134 to 1,250	No	ns	>0,9999
S. gordonii:TNZT - Control vs. *S. sanguinis*:TNZT - HF	0,2583	-0,7234 to 1,240	No	ns	>0,9999
S. gordonii:TNZT - Control vs. *S. sanguinis*:TNZT - HFAg	0,8117	-0,1701 to 1,793	No	ns	0,3208
S. gordonii:TNZT - Control vs. *A. naeslundii*:TAV - Control	-0,2133	-1,195 to 0,7684	No	ns	>0,9999
S. gordonii:TNZT - Control vs. *A. naeslundii*:TAV - CaP	-0,6083	-1,590 to 0,3734	No	ns	0,9040
S. gordonii:TNZT - Control vs. *A. naeslundii*:TAV - CaPAg	-0,7550	-1,737 to 0,2267	No	ns	0,4924
S. gordonii:TNZT - Control vs. *A. naeslundii*:TAV - HF	-0,04433	-1,074 to 0,9853	No	ns	>0,9999
S. gordonii:TNZT - Control vs. *A. naeslundii*:TAV - HFAg	0,1650	-0,8167 to 1,147	No	ns	>0,9999
S. gordonii:TNZT - Control vs. *A. naeslundii*:TNZT - Control	-0,7400	-1,722 to 0,2417	No	ns	0,5416
S. gordonii:TNZT - Control vs. *A. naeslundii*:TNZT - CaP	-0,9100	-1,892 to 0,07174	No	ns	0,1204
S. gordonii:TNZT - Control vs. *A. naeslundii*:TNZT - CaPAg	-0,6050	-1,587 to 0,3767	No	ns	0,9096
S. gordonii:TNZT - Control vs. *A. naeslundii*:TNZT - HF	-0,09500	-1,077 to 0,8867	No	ns	>0,9999
S. gordonii:TNZT - Control vs. *A. naeslundii*:TNZT - HFAg	-0,4343	-1,464 to 0,5953	No	ns	0,9998
S. gordonii:TNZT - Control vs. *P. gingivalis*:TAV - Control	-0,5850	-1,567 to 0,3967	No	ns	0,9385
**_S. gordonii_:TNZT - Control vs. _P. gingivalis_:TAV - CaP**	**-1,223**	**-2,205 to -0,2416**	**Yes**	**	**0,0012**
**_S. gordonii_:TNZT - Control vs. _P. gingivalis_:TAV - CaPAg**	**-1,620**	**-2,602 to -0,6383**	**Yes**	****	**<0,0001**
S. gordonii:TNZT - Control vs. *P. gingivalis*:TAV - HF	-0,9550	-1,937 to 0,02674	No	ns	0,0704
S. gordonii:TNZT - Control vs. *P. gingivalis*:TAV - HFAg	-0,9400	-1,922 to 0,04174	No	ns	0,0847
S. gordonii:TNZT - Control vs. *P. gingivalis*:TNZT - Control	-0,6943	-1,724 to 0,3353	No	ns	0,7847
**_S. gordonii_:TNZT - Control vs. _P. gingivalis_:TNZT - CaP**	**-1,893**	**-2,991 to -0,7957**	**Yes**	****	**<0,0001**
**_S. gordonii_:TNZT - Control vs. _P. gingivalis_:TNZT - CaPAg**	**-1,877**	**-2,858 to -0,8949**	**Yes**	****	**<0,0001**
**_S. gordonii_:TNZT - Control vs. _P. gingivalis_:TNZT - HF**	**-1,132**	**-2,113 to -0,1499**	**Yes**	**	**0,0056**
**_S. gordonii_:TNZT - Control vs. _P. gingivalis_:TNZT - HFAg**	**-1,572**	**-2,553 to -0,5899**	**Yes**	****	**<0,0001**
S. gordonii:TNZT - CaP vs. *S. gordonii*:TNZT - CaPAg	0,4300	-0,5517 to 1,412	No	ns	0,9996
S. gordonii:TNZT - CaP vs. *S. gordonii*:TNZT - HF	0,8000	-0,1817 to 1,782	No	ns	0,3535
S. gordonii:TNZT - CaP vs. *S. gordonii*:TNZT - HFAg	0,7050	-0,2767 to 1,687	No	ns	0,6563
**_S. gordonii_:TNZT - CaP vs. _S. sanguinis_:TAV - Control**	**1,013**	**0,03159 to 1,995**	**Yes**	*	**0,0327**
S. gordonii:TNZT - CaP vs. *S. sanguinis*:TAV - CaP	0,6217	-0,3601 to 1,603	No	ns	0,8795
S. gordonii:TNZT - CaP vs. *S. sanguinis*:TAV - CaPAg	0,7983	-0,1834 to 1,780	No	ns	0,3583
S. gordonii:TNZT - CaP vs. *S. sanguinis*:TAV - HF	0,6667	-0,3151 to 1,648	No	ns	0,7720
S. gordonii:TNZT - CaP vs. *S. sanguinis*:TAV - HFAg	0,9617	-0,02008 to 1,943	No	ns	0,0647
S. gordonii:TNZT - CaP vs. *S. sanguinis*:TNZT - Control	0,4267	-0,5551 to 1,408	No	ns	0,9997
S. gordonii:TNZT - CaP vs. *S. sanguinis*:TNZT - CaP	0,2500	-0,7317 to 1,232	No	ns	>0,9999
S. gordonii:TNZT - CaP vs. *S. sanguinis*:TNZT - CaPAg	0,8083	-0,1734 to 1,790	No	ns	0,3300
S. gordonii:TNZT - CaP vs. *S. sanguinis*:TNZT - HF	0,7983	-0,1834 to 1,780	No	ns	0,3583
**_S. gordonii_:TNZT - CaP vs. _S. sanguinis_:TNZT - HFAg**	**1,352**	**0,3699 to 2,333**	**Yes**	***	**0,0001**
S. gordonii:TNZT - CaP vs. *A. naeslundii*:TAV - Control	0,3267	-0,6551 to 1,308	No	ns	>0,9999
S. gordonii:TNZT - CaP vs. *A. naeslundii*:TAV - CaP	-0,06833	-1,050 to 0,9134	No	ns	>0,9999
S. gordonii:TNZT - CaP vs. *A. naeslundii*:TAV - CaPAg	-0,2150	-1,197 to 0,7667	No	ns	>0,9999
S. gordonii:TNZT - CaP vs. *A. naeslundii*:TAV - HF	0,4957	-0,5340 to 1,525	No	ns	0,9976
S. gordonii:TNZT - CaP vs. *A. naeslundii*:TAV - HFAg	0,7050	-0,2767 to 1,687	No	ns	0,6563
S. gordonii:TNZT - CaP vs. *A. naeslundii*:TNZT - Control	-0,2000	-1,182 to 0,7817	No	ns	>0,9999
S. gordonii:TNZT - CaP vs. *A. naeslundii*:TNZT - CaP	-0,3700	-1,352 to 0,6117	No	ns	>0,9999
S. gordonii:TNZT - CaP vs. *A. naeslundii*:TNZT - CaPAg	-0,06500	-1,047 to 0,9167	No	ns	>0,9999
S. gordonii:TNZT - CaP vs. *A. naeslundii*:TNZT - HF	0,4450	-0,5367 to 1,427	No	ns	0,9992
S. gordonii:TNZT - CaP vs. *A. naeslundii*:TNZT - HFAg	0,1057	-0,9240 to 1,135	No	ns	>0,9999
S. gordonii:TNZT - CaP vs. *P. gingivalis*:TAV - Control	-0,04500	-1,027 to 0,9367	No	ns	>0,9999
S. gordonii:TNZT - CaP vs. *P. gingivalis*:TAV - CaP	-0,6833	-1,665 to 0,2984	No	ns	0,7238
**_S. gordonii_:TNZT - CaP vs. _P. gingivalis_:TAV - CaPAg**	**-1,080**	**-2,062 to -0,09826**	**Yes**	*	**0,0125**
S. gordonii:TNZT - CaP vs. *P. gingivalis*:TAV - HF	-0,4150	-1,397 to 0,5667	No	ns	0,9998
S. gordonii:TNZT - CaP vs. *P. gingivalis*:TAV - HFAg	-0,4000	-1,382 to 0,5817	No	ns	>0,9999
S. gordonii:TNZT - CaP vs. *P. gingivalis*:TNZT - Control	-0,1543	-1,184 to 0,8753	No	ns	>0,9999
**_S. gordonii_:TNZT - CaP vs. _P. gingivalis_:TNZT - CaP**	**-1,353**	**-2,451 to -0,2557**	**Yes**	**	**0,0015**
**_S. gordonii_:TNZT - CaP vs. _P. gingivalis_:TNZT - CaPAg**	**-1,337**	**-2,318 to -0,3549**	**Yes**	***	**0,0002**
S. gordonii:TNZT - CaP vs. *P. gingivalis*:TNZT - HF	-0,5917	-1,573 to 0,3901	No	ns	0,9297
**_S. gordonii_:TNZT - CaP vs. _P. gingivalis_:TNZT - HFAg**	**-1,032**	**-2,013 to -0,04992**	**Yes**	*	**0,0253**
S. gordonii:TNZT - CaPAg vs. *S. gordonii*:TNZT - HF	0,3700	-0,6117 to 1,352	No	ns	>0,9999
S. gordonii:TNZT - CaPAg vs. *S. gordonii*:TNZT - HFAg	0,2750	-0,7067 to 1,257	No	ns	>0,9999
S. gordonii:TNZT - CaPAg vs. *S. sanguinis*:TAV - Control	0,5833	-0,3984 to 1,565	No	ns	0,9405
S. gordonii:TNZT - CaPAg vs. *S. sanguinis*:TAV - CaP	0,1917	-0,7901 to 1,173	No	ns	>0,9999
S. gordonii:TNZT - CaPAg vs. *S. sanguinis*:TAV - CaPAg	0,3683	-0,6134 to 1,350	No	ns	>0,9999

S. gordonii:TNZT - CaPAg vs. *S. sanguinis*:TAV - HF	0,2367	-0,7451 to 1,218	No	ns	>0,9999
S. gordonii:TNZT - CaPAg vs. *S. sanguinis*:TAV - HFAg	0,5317	-0,4501 to 1,513	No	ns	0,9829
S. gordonii:TNZT - CaPAg vs. *S. sanguinis*:TNZT - Control	-0,003333	-0,9851 to 0,9784	No	ns	>0,9999
S. gordonii:TNZT - CaPAg vs. *S. sanguinis*:TNZT - CaP	-0,1800	-1,162 to 0,8017	No	ns	>0,9999
S. gordonii:TNZT - CaPAg vs. *S. sanguinis*:TNZT - CaPAg	0,3783	-0,6034 to 1,360	No	ns	>0,9999
S. gordonii:TNZT - CaPAg vs. *S. sanguinis*:TNZT - HF	0,3683	-0,6134 to 1,350	No	ns	>0,9999
S. gordonii:TNZT - CaPAg vs. *S. sanguinis*:TNZT - HFAg	0,9217	-0,06008 to 1,903	No	ns	0,1053
S. gordonii:TNZT - CaPAg vs. *A. naeslundii*:TAV - Control	-0,1033	-1,085 to 0,8784	No	ns	>0,9999
S. gordonii:TNZT - CaPAg vs. *A. naeslundii*:TAV - CaP	-0,4983	-1,480 to 0,4834	No	ns	0,9939
S. gordonii:TNZT - CaPAg vs. *A. naeslundii*:TAV - CaPAg	-0,6450	-1,627 to 0,3367	No	ns	0,8283
S. gordonii:TNZT - CaPAg vs. *A. naeslundii*:TAV - HF	0,06567	-0,9640 to 1,095	No	ns	>0,9999
S. gordonii:TNZT - CaPAg vs. *A. naeslundii*:TAV - HFAg	0,2750	-0,7067 to 1,257	No	ns	>0,9999
S. gordonii:TNZT - CaPAg vs. *A. naeslundii*:TNZT - Control	-0,6300	-1,612 to 0,3517	No	ns	0,8624
S. gordonii:TNZT - CaPAg vs. *A. naeslundii*:TNZT - CaP	-0,8000	-1,782 to 0,1817	No	ns	0,3535
S. gordonii:TNZT - CaPAg vs. *A. naeslundii*:TNZT - CaPAg	-0,4950	-1,477 to 0,4867	No	ns	0,9945
S. gordonii:TNZT - CaPAg vs. *A. naeslundii*:TNZT - HF	0,01500	-0,9667 to 0,9967	No	ns	>0,9999
S. gordonii:TNZT - CaPAg vs. *A. naeslundii*:TNZT - HFAg	-0,3243	-1,354 to 0,7053	No	ns	>0,9999
S. gordonii:TNZT - CaPAg vs. *P. gingivalis*:TAV - Control	-0,4750	-1,457 to 0,5067	No	ns	0,9973
***S. gordonii*:TNZT - CaPAg vs. *P. gingivalis*:TAV - CaP**	**-1,113**	**-2,095 to -0,1316**	**Yes**	******	**0,0075**
***S. gordonii*:TNZT - CaPAg vs. *P. gingivalis*:TAV - CaPAg**	**-1,510**	**-2,492 to -0,5283**	**Yes**	********	**<0,0001**
S. gordonii:TNZT - CaPAg vs. *P. gingivalis*:TAV - HF	-0,8450	-1,827 to 0,1367	No	ns	0,2376
S. gordonii:TNZT - CaPAg vs. *P. gingivalis*:TAV - HFAg	-0,8300	-1,812 to 0,1517	No	ns	0,2731
S. gordonii:TNZT - CaPAg vs. *P. gingivalis*:TNZT - Control	-0,5843	-1,614 to 0,4453	No	ns	0,9669
***S. gordonii*:TNZT - CaPAg vs. *P. gingivalis*:TNZT - CaP**	**-1,783**	**-2,881 to -0,6857**	**Yes**	********	**<0,0001**
***S. gordonii*:TNZT - CaPAg vs. *P. gingivalis*:TNZT - CaPAg**	**-1,767**	**-2,748 to -0,7849**	**Yes**	********	**<0,0001**
***S. gordonii*:TNZT - CaPAg vs. *P. gingivalis*:TNZT - HF**	**-1,022**	**-2,003 to -0,03992**	**Yes**	*****	**0,0291**
***S. gordonii*:TNZT - CaPAg vs. *P. gingivalis*:TNZT - HFAg**	**-1,462**	**-2,443 to -0,4799**	**Yes**	********	**<0,0001**
S. gordonii:TNZT - HF vs. *S. gordonii*:TNZT - HFAg	-0,09500	-1,077 to 0,8867	No	ns	>0,9999
S. gordonii:TNZT - HF vs. *S. sanguinis*:TAV - Control	0,2133	-0,7684 to 1,195	No	ns	>0,9999
S. gordonii:TNZT - HF vs. *S. sanguinis*:TAV - CaP	-0,1783	-1,160 to 0,8034	No	ns	>0,9999
S. gordonii:TNZT - HF vs. *S. sanguinis*:TAV - CaPAg	-0,001667	-0,9834 to 0,9801	No	ns	>0,9999
S. gordonii:TNZT - HF vs. *S. sanguinis*:TAV - HF	-0,1333	-1,115 to 0,8484	No	ns	>0,9999
S. gordonii:TNZT - HF vs. *S. sanguinis*:TAV - HFAg	0,1617	-0,8201 to 1,143	No	ns	>0,9999
S. gordonii:TNZT - HF vs. *S. sanguinis*:TNZT - Control	-0,3733	-1,355 to 0,6084	No	ns	>0,9999
S. gordonii:TNZT - HF vs. *S. sanguinis*:TNZT - CaP	-0,5500	-1,532 to 0,4317	No	ns	0,9722
S. gordonii:TNZT - HF vs. *S. sanguinis*:TNZT - CaPAg	0,008333	-0,9734 to 0,9901	No	ns	>0,9999
S. gordonii:TNZT - HF vs. *S. sanguinis*:TNZT - HF	-0,001667	-0,9834 to 0,9801	No	ns	>0,9999
S. gordonii:TNZT - HF vs. *S. sanguinis*:TNZT - HFAg	0,5517	-0,4301 to 1,533	No	ns	0,9710
S. gordonii:TNZT - HF vs. *A. naeslundii*:TAV - Control	-0,4733	-1,455 to 0,5084	No	ns	0,9975
S. gordonii:TNZT - HF vs. *A. naeslundii*:TAV - CaP	-0,8683	-1,850 to 0,1134	No	ns	0,1887
***S. gordonii*:TNZT - HF vs. *A. naeslundii*:TAV - CaPAg**	**-1,015**	**-1,997 to -0,03326**	**Yes**	*****	**0,0319**
S. gordonii:TNZT - HF vs. *A. naeslundii*:TAV - HF	-0,3043	-1,334 to 0,7253	No	ns	>0,9999
S. gordonii:TNZT - HF vs. *A. naeslundii*:TAV - HFAg	-0,09500	-1,077 to 0,8867	No	ns	>0,9999
***S. gordonii*:TNZT - HF vs. *A. naeslundii*:TNZT - Control**	**-1,000**	**-1,982 to -0,01826**	**Yes**	*****	**0,0392**
***S. gordonii*:TNZT - HF vs. *A. naeslundii*:TNZT - CaP**	**-1,170**	**-2,152 to -0,1883**	**Yes**	******	**0,0030**
S. gordonii:TNZT - HF vs. *A. naeslundii*:TNZT - CaPAg	-0,8650	-1,847 to 0,1167	No	ns	0,1952
S. gordonii:TNZT - HF vs. *A. naeslundii*:TNZT - HF	-0,3550	-1,337 to 0,6267	No	ns	>0,9999
S. gordonii:TNZT - HF vs. *A. naeslundii*:TNZT - HFAg	-0,6943	-1,724 to 0,3353	No	ns	0,7847
S. gordonii:TNZT - HF vs. *P. gingivalis*:TAV - Control	-0,8450	-1,827 to 0,1367	No	ns	0,2376
***S. gordonii*:TNZT - HF vs. *P. gingivalis*:TAV - CaP**	**-1,483**	**-2,465 to -0,5016**	**Yes**	********	**<0,0001**
***S. gordonii*:TNZT - HF vs. *P. gingivalis*:TAV - CaPAg**	**-1,880**	**-2,862 to -0,8983**	**Yes**	********	**<0,0001**
***S. gordonii*:TNZT - HF vs. *P. gingivalis*:TAV - HF**	**-1,215**	**-2,197 to -0,2333**	**Yes**	******	**0,0014**
***S. gordonii*:TNZT - HF vs. *P. gingivalis*:TAV - HFAg**	**-1,200**	**-2,182 to -0,2183**	**Yes**	******	**0,0018**
S. gordonii:TNZT - HF vs. *P. gingivalis*:TNZT - Control	-0,9543	-1,984 to 0,07533	No	ns	0,1205
***S. gordonii*:TNZT - HF vs. *P. gingivalis*:TNZT - CaP**	**-2,153**	**-3,251 to -1,056**	**Yes**	********	**<0,0001**
***S. gordonii*:TNZT - HF vs. *P. gingivalis*:TNZT - CaPAg**	**-2,137**	**-3,118 to -1,155**	**Yes**	********	**<0,0001**
***S. gordonii*:TNZT - HF vs. *P. gingivalis*:TNZT - HF**	**-1,392**	**-2,373 to -0,4099**	**Yes**	********	**<0,0001**
***S. gordonii*:TNZT - HF vs. *P. gingivalis*:TNZT - HFAg**	**-1,832**	**-2,813 to -0,8499**	**Yes**	********	**<0,0001**
S. gordonii:TNZT - HFAg vs. *S. sanguinis*:TAV - Control	0,3083	-0,6734 to 1,290	No	ns	>0,9999
S. gordonii:TNZT - HFAg vs. *S. sanguinis*:TAV - CaP	-0,08333	-1,065 to 0,8984	No	ns	>0,9999
S. gordonii:TNZT - HFAg vs. *S. sanguinis*:TAV - CaPAg	0,09333	-0,8884 to 1,075	No	ns	>0,9999
S. gordonii:TNZT - HFAg vs. *S. sanguinis*:TAV - HF	-0,03833	-1,020 to 0,9434	No	ns	>0,9999
S. gordonii:TNZT - HFAg vs. *S. sanguinis*:TAV - HFAg	0,2567	-0,7251 to 1,238	No	ns	>0,9999
S. gordonii:TNZT - HFAg vs. *S. sanguinis*:TNZT - Control	-0,2783	-1,260 to 0,7034	No	ns	>0,9999
S. gordonii:TNZT - HFAg vs. *S. sanguinis*:TNZT - CaP	-0,4550	-1,437 to 0,5267	No	ns	0,9988
S. gordonii:TNZT - HFAg vs. *S. sanguinis*:TNZT - CaPAg	0,1033	-0,8784 to 1,085	No	ns	>0,9999
S. gordonii:TNZT - HFAg vs. *S. sanguinis*:TNZT - HF	0,09333	-0,8884 to 1,075	No	ns	>0,9999
S. gordonii:TNZT - HFAg vs. *S. sanguinis*:TNZT - HFAg	0,6467	-0,3351 to 1,628	No	ns	0,8243
S. gordonii:TNZT - HFAg vs. *A. naeslundii*:TAV - Control	-0,3783	-1,360 to 0,6034	No	ns	>0,9999
S. gordonii:TNZT - HFAg vs. *A. naeslundii*:TAV - CaP	-0,7733	-1,755 to 0,2084	No	ns	0,4337
S. gordonii:TNZT - HFAg vs. *A. naeslundii*:TAV - CaPAg	-0,9200	-1,902 to 0,06174	No	ns	0,1073
S. gordonii:TNZT - HFAg vs. *A. naeslundii*:TAV - HF	-0,2093	-1,239 to 0,8203	No	ns	>0,9999

S. gordonii:TNZT - HFAg vs. A. naeslundii:TAV - HFAg	0,000	-0,9817 to 0,9817	No	ns	>0,9999
S. gordonii:TNZT - HFAg vs. A. naeslundii:TNZT - Control	-0,9050	-1,887 to 0,07674	No	ns	0,1274
S. gordonii:TNZT - HFAg vs. A. naeslundii:TNZT - CaP	-1,075	-2,057 to -0,09326	Yes	*	0,0134
S. gordonii:TNZT - HFAg vs. A. naeslundii:TNZT - CaPAg	-0,7700	-1,752 to 0,2117	No	ns	0,4442
S. gordonii:TNZT - HFAg vs. A. naeslundii:TNZT - HF	-0,2600	-1,242 to 0,7217	No	ns	>0,9999
S. gordonii:TNZT - HFAg vs. A. naeslundii:TNZT - HFAg	-0,5993	-1,629 to 0,4303	No	ns	0,9539
S. gordonii:TNZT - HFAg vs. P. gingivalis:TAV - Control	-0,7500	-1,732 to 0,2317	No	ns	0,5087
S. gordonii:TNZT - HFAg vs. P. gingivalis:TAV - CaP	-1,388	-2,370 to -0,4066	Yes	****	<0,0001
S. gordonii:TNZT - HFAg vs. P. gingivalis:TAV - CaPAg	-1,785	-2,767 to -0,8033	Yes	****	<0,0001
S. gordonii:TNZT - HFAg vs. P. gingivalis:TAV - HF	-1,120	-2,102 to -0,1383	Yes	**	0,0067
S. gordonii:TNZT - HFAg vs. P. gingivalis:TAV - HFAg	-1,105	-2,087 to -0,1233	Yes	**	0,0085
S. gordonii:TNZT - HFAg vs. P. gingivalis:TNZT - Control	-0,8593	-1,889 to 0,1703	No	ns	0,3003
S. gordonii:TNZT - HFAg vs. P. gingivalis:TNZT - CaP	-2,058	-3,156 to -0,9607	Yes	****	<0,0001
S. gordonii:TNZT - HFAg vs. P. gingivalis:TNZT - CaPAg	-2,042	-3,023 to -1,060	Yes	****	<0,0001
S. gordonii:TNZT - HFAg vs. P. gingivalis:TNZT - HF	-1,297	-2,278 to -0,3149	Yes	***	0,0003
S. gordonii:TNZT - HFAg vs. P. gingivalis:TNZT - HFAg	-1,737	-2,718 to -0,7549	Yes	****	<0,0001
S. sanguinis:TAV - Control vs. S. sanguinis:TAV - CaP	-0,3917	-1,373 to 0,5901	No	ns	>0,9999
S. sanguinis:TAV - Control vs. S. sanguinis:TAV - CaPAg	-0,2150	-1,197 to 0,7667	No	ns	>0,9999
S. sanguinis:TAV - Control vs. S. sanguinis:TAV - HF	-0,3467	-1,328 to 0,6351	No	ns	>0,9999
S. sanguinis:TAV - Control vs. S. sanguinis:TAV - HFAg	-0,05167	-1,033 to 0,9301	No	ns	>0,9999
S. sanguinis:TAV - Control vs. S. sanguinis:TNZT - Control	-0,5867	-1,568 to 0,3951	No	ns	0,9364
S. sanguinis:TAV - Control vs. S. sanguinis:TNZT - CaP	-0,7633	-1,745 to 0,2184	No	ns	0,4655
S. sanguinis:TAV - Control vs. S. sanguinis:TNZT - CaPAg	-0,2050	-1,187 to 0,7767	No	ns	>0,9999
S. sanguinis:TAV - Control vs. S. sanguinis:TNZT - HF	-0,2150	-1,197 to 0,7667	No	ns	>0,9999
S. sanguinis:TAV - Control vs. S. sanguinis:TNZT - HFAg	0,3383	-0,6434 to 1,320	No	ns	>0,9999
S. sanguinis:TAV - Control vs. A. naeslundii:TAV - Control	-0,6867	-1,668 to 0,2951	No	ns	0,7137
S. sanguinis:TAV - Control vs. A. naeslundii:TAV - CaP	-1,082	-2,063 to -0,09992	Yes	*	0,0121
S. sanguinis:TAV - Control vs. A. naeslundii:TAV - CaPAg	-1,228	-2,210 to -0,2466	Yes	**	0,0011
S. sanguinis:TAV - Control vs. A. naeslundii:TAV - HF	-0,5177	-1,547 to 0,5120	No	ns	0,9948
S. sanguinis:TAV - Control vs. A. naeslundii:TAV - HFAg	-0,3083	-1,290 to 0,6734	No	ns	>0,9999
S. sanguinis:TAV - Control vs. A. naeslundii:TNZT - Control	-1,213	-2,195 to -0,2316	Yes	**	0,0014
S. sanguinis:TAV - Control vs. A. naeslundii:TNZT - CaP	-1,383	-2,365 to -0,4016	Yes	****	<0,0001
S. sanguinis:TAV - Control vs. A. naeslundii:TNZT - CaPAg	-1,078	-2,060 to -0,09659	Yes	*	0,0128
S. sanguinis:TAV - Control vs. A. naeslundii:TNZT - HF	-0,5683	-1,550 to 0,4134	No	ns	0,9570
S. sanguinis:TAV - Control vs. A. naeslundii:TNZT - HFAg	-0,9077	-1,937 to 0,1220	No	ns	0,1944
S. sanguinis:TAV - Control vs. P. gingivalis:TAV - Control	-1,058	-2,040 to -0,07659	Yes	*	0,0172
S. sanguinis:TAV - Control vs. P. gingivalis:TAV - CaP	-1,697	-2,678 to -0,7149	Yes	****	<0,0001
S. sanguinis:TAV - Control vs. P. gingivalis:TAV - CaPAg	-2,093	-3,075 to -1,112	Yes	****	<0,0001
S. sanguinis:TAV - Control vs. P. gingivalis:TAV - HF	-1,428	-2,410 to -0,4466	Yes	****	<0,0001
S. sanguinis:TAV - Control vs. P. gingivalis:TAV - HFAg	-1,413	-2,395 to -0,4316	Yes	****	<0,0001
S. sanguinis:TAV - Control vs. P. gingivalis:TNZT - Control	-1,168	-2,197 to -0,1380	Yes	**	0,0075
S. sanguinis:TAV - Control vs. P. gingivalis:TNZT - CaP	-2,367	-3,464 to -1,269	Yes	****	<0,0001
S. sanguinis:TAV - Control vs. P. gingivalis:TNZT - CaPAg	-2,350	-3,332 to -1,368	Yes	****	<0,0001
S. sanguinis:TAV - Control vs. P. gingivalis:TNZT - HF	-1,605	-2,587 to -0,6233	Yes	****	<0,0001
S. sanguinis:TAV - Control vs. P. gingivalis:TNZT - HFAg	-2,045	-3,027 to -1,063	Yes	****	<0,0001
S. sanguinis:TAV - CaP vs. S. sanguinis:TAV - CaPAg	0,1767	-0,8051 to 1,158	No	ns	>0,9999
S. sanguinis:TAV - CaP vs. S. sanguinis:TAV - HF	0,04500	-0,9367 to 1,027	No	ns	>0,9999
S. sanguinis:TAV - CaP vs. S. sanguinis:TAV - HFAg	0,3400	-0,6417 to 1,322	No	ns	>0,9999
S. sanguinis:TAV - CaP vs. S. sanguinis:TNZT - Control	-0,1950	-1,177 to 0,7867	No	ns	>0,9999
S. sanguinis:TAV - CaP vs. S. sanguinis:TNZT - CaP	-0,3717	-1,353 to 0,6101	No	ns	>0,9999
S. sanguinis:TAV - CaP vs. S. sanguinis:TNZT - CaPAg	0,1867	-0,7951 to 1,168	No	ns	>0,9999
S. sanguinis:TAV - CaP vs. S. sanguinis:TNZT - HF	0,1767	-0,8051 to 1,158	No	ns	>0,9999
S. sanguinis:TAV - CaP vs. S. sanguinis:TNZT - HFAg	0,7300	-0,2517 to 1,712	No	ns	0,5747
S. sanguinis:TAV - CaP vs. A. naeslundii:TAV - Control	-0,2950	-1,277 to 0,6867	No	ns	>0,9999
S. sanguinis:TAV - CaP vs. A. naeslundii:TAV - CaP	-0,6900	-1,672 to 0,2917	No	ns	0,7035
S. sanguinis:TAV - CaP vs. A. naeslundii:TAV - CaPAg	-0,8367	-1,818 to 0,1451	No	ns	0,2570
S. sanguinis:TAV - CaP vs. A. naeslundii:TAV - HF	-0,1260	-1,156 to 0,9037	No	ns	>0,9999
S. sanguinis:TAV - CaP vs. A. naeslundii:TAV - HFAg	0,08333	-0,8984 to 1,065	No	ns	>0,9999
S. sanguinis:TAV - CaP vs. A. naeslundii:TNZT - Control	-0,8217	-1,803 to 0,1601	No	ns	0,2943
S. sanguinis:TAV - CaP vs. A. naeslundii:TNZT - CaP	-0,9917	-1,973 to -0,009922	Yes	*	0,0439
S. sanguinis:TAV - CaP vs. A. naeslundii:TNZT - CaPAg	-0,6867	-1,668 to 0,2951	No	ns	0,7137
S. sanguinis:TAV - CaP vs. A. naeslundii:TNZT - HF	-0,1767	-1,158 to 0,8051	No	ns	>0,9999
S. sanguinis:TAV - CaP vs. A. naeslundii:TNZT - HFAg	-0,5160	-1,546 to 0,5137	No	ns	0,9951
S. sanguinis:TAV - CaP vs. P. gingivalis:TAV - Control	-0,6667	-1,648 to 0,3151	No	ns	0,7720
S. sanguinis:TAV - CaP vs. P. gingivalis:TAV - CaP	-1,305	-2,287 to -0,3233	Yes	***	0,0003
S. sanguinis:TAV - CaP vs. P. gingivalis:TAV - CaPAg	-1,702	-2,683 to -0,7199	Yes	****	<0,0001
S. sanguinis:TAV - CaP vs. P. gingivalis:TAV - HF	-1,037	-2,018 to -0,05492	Yes	*	0,0236
S. sanguinis:TAV - CaP vs. P. gingivalis:TAV - HFAg	-1,022	-2,003 to -0,03992	Yes	*	0,0291
S. sanguinis:TAV - CaP vs. P. gingivalis:TNZT - Control	-0,7760	-1,806 to 0,2537	No	ns	0,5420
S. sanguinis:TAV - CaP vs. P. gingivalis:TNZT - CaP	-1,975	-3,073 to -0,8774	Yes	****	<0,0001
S. sanguinis:TAV - CaP vs. P. gingivalis:TNZT - CaPAg	-1,958	-2,940 to -0,9766	Yes	****	<0,0001
S. sanguinis:TAV - CaP vs. P. gingivalis:TNZT - HF	-1,213	-2,195 to -0,2316	Yes	**	0,0014

**S. sanguinis:TAV - CaP vs. P. gingivalis:TNZT - HFAg**	**-1,653**	**-2,635 to -0,6716**	**Yes**	********	**<0,0001**
S. sanguinis:TAV - CaPAg vs. *S. sanguinis*:TAV - HF	-0,1317	-1,113 to 0,8501	No	ns	>0,9999
S. sanguinis:TAV - CaPAg vs. *S. sanguinis*:TAV - HFAg	0,1633	-0,8184 to 1,145	No	ns	>0,9999
S. sanguinis:TAV - CaPAg vs. *S. sanguinis*:TNZT - Control	-0,3717	-1,353 to 0,6101	No	ns	>0,9999
S. sanguinis:TAV - CaPAg vs. *S. sanguinis*:TNZT - CaP	-0,5483	-1,530 to 0,4334	No	ns	0,9734
S. sanguinis:TAV - CaPAg vs. *S. sanguinis*:TNZT - CaPAg	0,01000	-0,9717 to 0,9917	No	ns	>0,9999
S. sanguinis:TAV - CaPAg vs. *S. sanguinis*:TNZT - HF	0,000	-0,9817 to 0,9817	No	ns	>0,9999
S. sanguinis:TAV - CaPAg vs. *S. sanguinis*:TNZT - HFAg	0,5533	-0,4284 to 1,535	No	ns	0,9698
S. sanguinis:TAV - CaPAg vs. *A. naeslundii*:TAV - Control	-0,4717	-1,453 to 0,5101	No	ns	0,9977
S. sanguinis:TAV - CaPAg vs. *A. naeslundii*:TAV - CaP	-0,8667	-1,848 to 0,1151	No	ns	0,1920
**S. sanguinis:TAV - CaPAg vs. A. naeslundii:TAV - CaPAg**	**-1,013**	**-1,995 to -0,03159**	**Yes**	*****	**0,0327**
S. sanguinis:TAV - CaPAg vs. *A. naeslundii*:TAV - HF	-0,3027	-1,332 to 0,7270	No	ns	>0,9999
S. sanguinis:TAV - CaPAg vs. *A. naeslundii*:TAV - HFAg	-0,09333	-1,075 to 0,8884	No	ns	>0,9999
**S. sanguinis:TAV - CaPAg vs. A. naeslundii:TNZT - Control**	**-0,9983**	**-1,980 to -0,01659**	**Yes**	*****	**0,0401**
**S. sanguinis:TAV - CaPAg vs. A. naeslundii:TNZT - CaP**	**-1,168**	**-2,150 to -0,1866**	**Yes**	******	**0,0031**
S. sanguinis:TAV - CaPAg vs. *A. naeslundii*:TNZT - CaPAg	-0,8633	-1,845 to 0,1184	No	ns	0,1986
S. sanguinis:TAV - CaPAg vs. *A. naeslundii*:TNZT - HF	-0,3533	-1,335 to 0,6284	No	ns	>0,9999
S. sanguinis:TAV - CaPAg vs. *A. naeslundii*:TNZT - HFAg	-0,6927	-1,722 to 0,3370	No	ns	0,7890
S. sanguinis:TAV - CaPAg vs. *P. gingivalis*:TAV - Control	-0,8433	-1,825 to 0,1384	No	ns	0,2414
**S. sanguinis:TAV - CaPAg vs. P. gingivalis:TAV - CaP**	**-1,482**	**-2,463 to -0,4999**	**Yes**	********	**<0,0001**
**S. sanguinis:TAV - CaPAg vs. P. gingivalis:TAV - CaPAg**	**-1,878**	**-2,860 to -0,8966**	**Yes**	********	**<0,0001**
**S. sanguinis:TAV - CaPAg vs. P. gingivalis:TAV - HF**	**-1,213**	**-2,195 to -0,2316**	**Yes**	******	**0,0014**
**S. sanguinis:TAV - CaPAg vs. P. gingivalis:TAV - HFAg**	**-1,198**	**-2,180 to -0,2166**	**Yes**	******	**0,0018**
S. sanguinis:TAV - CaPAg vs. *P. gingivalis*:TNZT - Control	-0,9527	-1,982 to 0,07700	No	ns	0,1227
**S. sanguinis:TAV - CaPAg vs. P. gingivalis:TNZT - CaP**	**-2,152**	**-3,249 to -1,054**	**Yes**	********	**<0,0001**
**S. sanguinis:TAV - CaPAg vs. P. gingivalis:TNZT - CaPAg**	**-2,135**	**-3,117 to -1,153**	**Yes**	********	**<0,0001**
**S. sanguinis:TAV - CaPAg vs. P. gingivalis:TNZT - HF**	**-1,390**	**-2,372 to -0,4083**	**Yes**	********	**<0,0001**
**S. sanguinis:TAV - CaPAg vs. P. gingivalis:TNZT - HFAg**	**-1,830**	**-2,812 to -0,8483**	**Yes**	********	**<0,0001**
S. sanguinis:TAV - HF vs. *S. sanguinis*:TAV - HFAg	0,2950	-0,6867 to 1,277	No	ns	>0,9999
S. sanguinis:TAV - HF vs. *S. sanguinis*:TNZT - Control	-0,2400	-1,222 to 0,7417	No	ns	>0,9999
S. sanguinis:TAV - HF vs. *S. sanguinis*:TNZT - CaP	-0,4167	-1,398 to 0,5651	No	ns	0,9998
S. sanguinis:TAV - HF vs. *S. sanguinis*:TNZT - CaPAg	0,1417	-0,8401 to 1,123	No	ns	>0,9999
S. sanguinis:TAV - HF vs. *S. sanguinis*:TNZT - HF	0,1317	-0,8501 to 1,113	No	ns	>0,9999
S. sanguinis:TAV - HF vs. *S. sanguinis*:TNZT - HFAg	0,6850	-0,2967 to 1,667	No	ns	0,7188
S. sanguinis:TAV - HF vs. *A. naeslundii*:TAV - Control	-0,3400	-1,322 to 0,6417	No	ns	>0,9999
S. sanguinis:TAV - HF vs. *A. naeslundii*:TAV - CaP	-0,7350	-1,717 to 0,2467	No	ns	0,5581
S. sanguinis:TAV - HF vs. *A. naeslundii*:TAV - CaPAg	-0,8817	-1,863 to 0,1001	No	ns	0,1643
S. sanguinis:TAV - HF vs. *A. naeslundii*:TAV - HF	-0,1710	-1,201 to 0,8587	No	ns	>0,9999
S. sanguinis:TAV - HF vs. *A. naeslundii*:TAV - HFAg	0,03833	-0,9434 to 1,020	No	ns	>0,9999
S. sanguinis:TAV - HF vs. *A. naeslundii*:TNZT - Control	-0,8667	-1,848 to 0,1151	No	ns	0,1920
**S. sanguinis:TAV - HF vs. A. naeslundii:TNZT - CaP**	**-1,037**	**-2,018 to -0,05492**	**Yes**	*****	**0,0236**
S. sanguinis:TAV - HF vs. *A. naeslundii*:TNZT - CaPAg	-0,7317	-1,713 to 0,2501	No	ns	0,5692
S. sanguinis:TAV - HF vs. *A. naeslundii*:TNZT - HF	-0,2217	-1,203 to 0,7601	No	ns	>0,9999
S. sanguinis:TAV - HF vs. *A. naeslundii*:TNZT - HFAg	-0,5610	-1,591 to 0,4687	No	ns	0,9813
S. sanguinis:TAV - HF vs. *P. gingivalis*:TAV - Control	-0,7117	-1,693 to 0,2701	No	ns	0,6348
**S. sanguinis:TAV - HF vs. P. gingivalis:TAV - CaP**	**-1,350**	**-2,332 to -0,3683**	**Yes**	*******	**0,0001**
**S. sanguinis:TAV - HF vs. P. gingivalis:TAV - CaPAg**	**-1,747**	**-2,728 to -0,7649**	**Yes**	********	**<0,0001**
**S. sanguinis:TAV - HF vs. P. gingivalis:TAV - HF**	**-1,082**	**-2,063 to -0,09992**	**Yes**	*****	**0,0121**
**S. sanguinis:TAV - HF vs. P. gingivalis:TAV - HFAg**	**-1,067**	**-2,048 to -0,08492**	**Yes**	*****	**0,0152**
S. sanguinis:TAV - HF vs. *P. gingivalis*:TNZT - Control	-0,8210	-1,851 to 0,2087	No	ns	0,4044
**S. sanguinis:TAV - HF vs. P. gingivalis:TNZT - CaP**	**-2,020**	**-3,118 to -0,9224**	**Yes**	********	**<0,0001**
**S. sanguinis:TAV - HF vs. P. gingivalis:TNZT - CaPAg**	**-2,003**	**-2,985 to -1,022**	**Yes**	********	**<0,0001**
**S. sanguinis:TAV - HF vs. P. gingivalis:TNZT - HF**	**-1,258**	**-2,240 to -0,2766**	**Yes**	*******	**0,0006**
**S. sanguinis:TAV - HF vs. P. gingivalis:TNZT - HFAg**	**-1,698**	**-2,680 to -0,7166**	**Yes**	********	**<0,0001**
S. sanguinis:TAV - HFAg vs. *S. sanguinis*:TNZT - Control	-0,5350	-1,517 to 0,4467	No	ns	0,9813
S. sanguinis:TAV - HFAg vs. *S. sanguinis*:TNZT - CaP	-0,7117	-1,693 to 0,2701	No	ns	0,6348
S. sanguinis:TAV - HFAg vs. *S. sanguinis*:TNZT - CaPAg	-0,1533	-1,135 to 0,8284	No	ns	>0,9999
S. sanguinis:TAV - HFAg vs. *S. sanguinis*:TNZT - HF	-0,1633	-1,145 to 0,8184	No	ns	>0,9999
S. sanguinis:TAV - HFAg vs. *S. sanguinis*:TNZT - HFAg	0,3900	-0,5917 to 1,372	No	ns	>0,9999
S. sanguinis:TAV - HFAg vs. *A. naeslundii*:TAV - Control	-0,6350	-1,617 to 0,3467	No	ns	0,8516
**S. sanguinis:TAV - HFAg vs. A. naeslundii:TAV - CaP**	**-1,030**	**-2,012 to -0,04826**	**Yes**	*****	**0,0259**
**S. sanguinis:TAV - HFAg vs. A. naeslundii:TAV - CaPAg**	**-1,177**	**-2,158 to -0,1949**	**Yes**	******	**0,0027**
S. sanguinis:TAV - HFAg vs. *A. naeslundii*:TAV - HF	-0,4660	-1,496 to 0,5637	No	ns	0,9993
S. sanguinis:TAV - HFAg vs. *A. naeslundii*:TAV - HFAg	-0,2567	-1,238 to 0,7251	No	ns	>0,9999
**S. sanguinis:TAV - HFAg vs. A. naeslundii:TNZT - Control**	**-1,162**	**-2,143 to -0,1799**	**Yes**	******	**0,0034**
**S. sanguinis:TAV - HFAg vs. A. naeslundii:TNZT - CaP**	**-1,332**	**-2,313 to -0,3499**	**Yes**	*******	**0,0002**
**S. sanguinis:TAV - HFAg vs. A. naeslundii:TNZT - CaPAg**	**-1,027**	**-2,008 to -0,04492**	**Yes**	*****	**0,0271**
S. sanguinis:TAV - HFAg vs. *A. naeslundii*:TNZT - HF	-0,5167	-1,498 to 0,4651	No	ns	0,9890
S. sanguinis:TAV - HFAg vs. *A. naeslundii*:TNZT - HFAg	-0,8560	-1,886 to 0,1737	No	ns	0,3087
**S. sanguinis:TAV - HFAg vs. P. gingivalis:TAV - Control**	**-1,007**	**-1,988 to -0,02492**	**Yes**	*****	**0,0358**
**S. sanguinis:TAV - HFAg vs. P. gingivalis:TAV - CaP**	**-1,645**	**-2,627 to -0,6633**	**Yes**	********	**<0,0001**
**S. sanguinis:TAV - HFAg vs. P. gingivalis:TAV - CaPAg**	**-2,042**	**-3,023 to -1,060**	**Yes**	********	**<0,0001**

**_S. sanguinis_:TAV - HFAg vs. _P. gingivalis_:TAV - HF**	**-1,377**	**-2,358 to -0,3949**	**Yes**	********	**<0,0001**
**_S. sanguinis_:TAV - HFAg vs. _P. gingivalis_:TAV - HFAg**	**-1,362**	**-2,343 to -0,3799**	**Yes**	********	**<0,0001**
**_S. sanguinis_:TAV - HFAg vs. _P. gingivalis_:TNZT - Control**	**-1,116**	**-2,146 to -0,08634**	**Yes**	*****	**0,0158**
**_S. sanguinis_:TAV - HFAg vs. _P. gingivalis_:TNZT - CaP**	**-2,315**	**-3,413 to -1,217**	**Yes**	********	**<0,0001**
**_S. sanguinis_:TAV - HFAg vs. _P. gingivalis_:TNZT - CaPAg**	**-2,298**	**-3,280 to -1,317**	**Yes**	********	**<0,0001**
**_S. sanguinis_:TAV - HFAg vs. _P. gingivalis_:TNZT - HF**	**-1,553**	**-2,535 to -0,5716**	**Yes**	********	**<0,0001**
**_S. sanguinis_:TAV - HFAg vs. _P. gingivalis_:TNZT - HFAg**	**-1,993**	**-2,975 to -1,012**	**Yes**	********	**<0,0001**
S. sanguinis:TNZT - Control vs. _S. sanguinis_:TNZT - CaP	-0,1767	-1,158 to 0,8051	No	ns	>0,9999
S. sanguinis:TNZT - Control vs. _S. sanguinis_:TNZT - CaPAg	0,3817	-0,6001 to 1,363	No	ns	>0,9999
S. sanguinis:TNZT - Control vs. _S. sanguinis_:TNZT - HF	0,3717	-0,6101 to 1,353	No	ns	>0,9999
S. sanguinis:TNZT - Control vs. _S. sanguinis_:TNZT - HFAg	0,9250	-0,05674 to 1,907	No	ns	0,1013
S. sanguinis:TNZT - Control vs. _A. naeslundii_:TAV - Control	-0,1000	-1,082 to 0,8817	No	ns	>0,9999
S. sanguinis:TNZT - Control vs. _A. naeslundii_:TAV - CaP	-0,4950	-1,477 to 0,4867	No	ns	0,9945
S. sanguinis:TNZT - Control vs. _A. naeslundii_:TAV - CaPAg	-0,6417	-1,623 to 0,3401	No	ns	0,8363
S. sanguinis:TNZT - Control vs. _A. naeslundii_:TAV - HF	0,06900	-0,9607 to 1,099	No	ns	>0,9999
S. sanguinis:TNZT - Control vs. _A. naeslundii_:TAV - HFAg	0,2783	-0,7034 to 1,260	No	ns	>0,9999
S. sanguinis:TNZT - Control vs. _A. naeslundii_:TNZT - Control	-0,6267	-1,608 to 0,3551	No	ns	0,8694
S. sanguinis:TNZT - Control vs. _A. naeslundii_:TNZT - CaP	-0,7967	-1,778 to 0,1851	No	ns	0,3631
S. sanguinis:TNZT - Control vs. _A. naeslundii_:TNZT - CaPAg	-0,4917	-1,473 to 0,4901	No	ns	0,9951
S. sanguinis:TNZT - Control vs. _A. naeslundii_:TNZT - HF	0,01833	-0,9634 to 1,000	No	ns	>0,9999
S. sanguinis:TNZT - Control vs. _A. naeslundii_:TNZT - HFAg	-0,3210	-1,351 to 0,7087	No	ns	>0,9999
S. sanguinis:TNZT - Control vs. _P. gingivalis_:TAV - Control	-0,4717	-1,453 to 0,5101	No	ns	0,9977
**_S. sanguinis_:TNZT - Control vs. _P. gingivalis_:TAV - CaP**	**-1,110**	**-2,092 to -0,1283**	**Yes**	******	**0,0079**
**_S. sanguinis_:TNZT - Control vs. _P. gingivalis_:TAV - CaPAg**	**-1,507**	**-2,488 to -0,5249**	**Yes**	********	**<0,0001**
S. sanguinis:TNZT - Control vs. _P. gingivalis_:TAV - HF	-0,8417	-1,823 to 0,1401	No	ns	0,2452
S. sanguinis:TNZT - Control vs. _P. gingivalis_:TAV - HFAg	-0,8267	-1,808 to 0,1551	No	ns	0,2815
S. sanguinis:TNZT - Control vs. _P. gingivalis_:TNZT - Control	-0,5810	-1,611 to 0,4487	No	ns	0,9694
**_S. sanguinis_:TNZT - Control vs. _P. gingivalis_:TNZT - CaP**	**-1,780**	**-2,878 to -0,6824**	**Yes**	********	**<0,0001**
**_S. sanguinis_:TNZT - Control vs. _P. gingivalis_:TNZT - CaPAg**	**-1,763**	**-2,745 to -0,7816**	**Yes**	********	**<0,0001**
**_S. sanguinis_:TNZT - Control vs. _P. gingivalis_:TNZT - HF**	**-1,018**	**-2,000 to -0,03659**	**Yes**	*****	**0,0305**
**_S. sanguinis_:TNZT - Control vs. _P. gingivalis_:TNZT - HFAg**	**-1,458**	**-2,440 to -0,4766**	**Yes**	********	**<0,0001**
S. sanguinis:TNZT - CaP vs. _S. sanguinis_:TNZT - CaPAg	0,5583	-0,4234 to 1,540	No	ns	0,9659
S. sanguinis:TNZT - CaP vs. _S. sanguinis_:TNZT - HF	0,5483	-0,4334 to 1,530	No	ns	0,9734
**_S. sanguinis_:TNZT - CaP vs. _S. sanguinis_:TNZT - HFAg**	**1,102**	**0,1199 to 2,083**	**Yes**	******	**0,0089**
S. sanguinis:TNZT - CaP vs. _A. naeslundii_:TAV - Control	0,07667	-0,9051 to 1,058	No	ns	>0,9999
S. sanguinis:TNZT - CaP vs. _A. naeslundii_:TAV - CaP	-0,3183	-1,300 to 0,6634	No	ns	>0,9999
S. sanguinis:TNZT - CaP vs. _A. naeslundii_:TAV - CaPAg	-0,4650	-1,447 to 0,5167	No	ns	0,9982
S. sanguinis:TNZT - CaP vs. _A. naeslundii_:TAV - HF	0,2457	-0,7840 to 1,275	No	ns	>0,9999
S. sanguinis:TNZT - CaP vs. _A. naeslundii_:TAV - HFAg	0,4550	-0,5267 to 1,437	No	ns	0,9988
S. sanguinis:TNZT - CaP vs. _A. naeslundii_:TNZT - Control	-0,4500	-1,432 to 0,5317	No	ns	0,9990
S. sanguinis:TNZT - CaP vs. _A. naeslundii_:TNZT - CaP	-0,6200	-1,602 to 0,3617	No	ns	0,8828
S. sanguinis:TNZT - CaP vs. _A. naeslundii_:TNZT - CaPAg	-0,3150	-1,297 to 0,6667	No	ns	>0,9999
S. sanguinis:TNZT - CaP vs. _A. naeslundii_:TNZT - HF	0,1950	-0,7867 to 1,177	No	ns	>0,9999
S. sanguinis:TNZT - CaP vs. _A. naeslundii_:TNZT - HFAg	-0,1443	-1,174 to 0,8853	No	ns	>0,9999
S. sanguinis:TNZT - CaP vs. _P. gingivalis_:TAV - Control	-0,2950	-1,277 to 0,6867	No	ns	>0,9999
S. sanguinis:TNZT - CaP vs. _P. gingivalis_:TAV - CaP	-0,9333	-1,915 to 0,04841	No	ns	0,0917
**_S. sanguinis_:TNZT - CaP vs. _P. gingivalis_:TAV - CaPAg**	**-1,330**	**-2,312 to -0,3483**	**Yes**	*******	**0,0002**
S. sanguinis:TNZT - CaP vs. _P. gingivalis_:TAV - HF	-0,6650	-1,647 to 0,3167	No	ns	0,7766
S. sanguinis:TNZT - CaP vs. _P. gingivalis_:TAV - HFAg	-0,6500	-1,632 to 0,3317	No	ns	0,8161
S. sanguinis:TNZT - CaP vs. _P. gingivalis_:TNZT - Control	-0,4043	-1,434 to 0,6253	No	ns	>0,9999
**_S. sanguinis_:TNZT - CaP vs. _P. gingivalis_:TNZT - CaP**	**-1,603**	**-2,701 to -0,5057**	**Yes**	********	**<0,0001**
**_S. sanguinis_:TNZT - CaP vs. _P. gingivalis_:TNZT - CaPAg**	**-1,587**	**-2,568 to -0,6049**	**Yes**	********	**<0,0001**
S. sanguinis:TNZT - CaP vs. _P. gingivalis_:TNZT - HF	-0,8417	-1,823 to 0,1401	No	ns	0,2452
**_S. sanguinis_:TNZT - CaP vs. _P. gingivalis_:TNZT - HFAg**	**-1,282**	**-2,263 to -0,2999**	**Yes**	*******	**0,0004**
S. sanguinis:TNZT - CaPAg vs. _S. sanguinis_:TNZT - HF	-0,01000	-0,9917 to 0,9717	No	ns	>0,9999
S. sanguinis:TNZT - CaPAg vs. _S. sanguinis_:TNZT - HFAg	0,5433	-0,4384 to 1,525	No	ns	0,9766
S. sanguinis:TNZT - CaPAg vs. _A. naeslundii_:TAV - Control	-0,4817	-1,463 to 0,5001	No	ns	0,9966
S. sanguinis:TNZT - CaPAg vs. _A. naeslundii_:TAV - CaP	-0,8767	-1,858 to 0,1051	No	ns	0,1732
**_S. sanguinis_:TNZT - CaPAg vs. _A. naeslundii_:TAV - CaPAg**	**-1,023**	**-2,005 to -0,04159**	**Yes**	*****	**0,0284**
S. sanguinis:TNZT - CaPAg vs. _A. naeslundii_:TAV - HF	-0,3127	-1,342 to 0,7170	No	ns	>0,9999
S. sanguinis:TNZT - CaPAg vs. _A. naeslundii_:TAV - HFAg	-0,1033	-1,085 to 0,8784	No	ns	>0,9999
**_S. sanguinis_:TNZT - CaPAg vs. _A. naeslundii_:TNZT - Control**	**-1,008**	**-1,990 to -0,02659**	**Yes**	*****	**0,0350**
**_S. sanguinis_:TNZT - CaPAg vs. _A. naeslundii_:TNZT - CaP**	**-1,178**	**-2,160 to -0,1966**	**Yes**	******	**0,0026**
S. sanguinis:TNZT - CaPAg vs. _A. naeslundii_:TNZT - CaPAg	-0,8733	-1,855 to 0,1084	No	ns	0,1793
S. sanguinis:TNZT - CaPAg vs. _A. naeslundii_:TNZT - HF	-0,3633	-1,345 to 0,6184	No	ns	>0,9999
S. sanguinis:TNZT - CaPAg vs. _A. naeslundii_:TNZT - HFAg	-0,7027	-1,732 to 0,3270	No	ns	0,7628
S. sanguinis:TNZT - CaPAg vs. _P. gingivalis_:TAV - Control	-0,8533	-1,835 to 0,1284	No	ns	0,2193
**_S. sanguinis_:TNZT - CaPAg vs. _P. gingivalis_:TAV - CaP**	**-1,492**	**-2,473 to -0,5099**	**Yes**	********	**<0,0001**
**_S. sanguinis_:TNZT - CaPAg vs. _P. gingivalis_:TAV - CaPAg**	**-1,888**	**-2,870 to -0,9066**	**Yes**	********	**<0,0001**
**_S. sanguinis_:TNZT - CaPAg vs. _P. gingivalis_:TAV - HF**	**-1,223**	**-2,205 to -0,2416**	**Yes**	******	**0,0012**
**_S. sanguinis_:TNZT - CaPAg vs. _P. gingivalis_:TAV - HFAg**	**-1,208**	**-2,190 to -0,2266**	**Yes**	******	**0,0016**
S. sanguinis:TNZT - CaPAg vs. _P. gingivalis_:TNZT - Control	-0,9627	-1,992 to 0,06700	No	ns	0,1100

***S. sanguinis*:TNZT - CaPAg vs. *P. gingivalis*:TNZT - CaP**	**-2,162**	**-3,259 to -1,064**	**Yes**	********	**<0,0001**
***S. sanguinis*:TNZT - CaPAg vs. *P. gingivalis*:TNZT - CaPAg**	**-2,145**	**-3,127 to -1,163**	**Yes**	********	**<0,0001**
***S. sanguinis*:TNZT - CaPAg vs. *P. gingivalis*:TNZT - HF**	**-1,400**	**-2,382 to -0,4183**	**Yes**	********	**<0,0001**
***S. sanguinis*:TNZT - CaPAg vs. *P. gingivalis*:TNZT - HFAg**	**-1,840**	**-2,822 to -0,8583**	**Yes**	********	**<0,0001**
S. sanguinis:TNZT - HF vs. *S. sanguinis*:TNZT - HFAg	0,5533	-0,4284 to 1,535	No	ns	0,9698
S. sanguinis:TNZT - HF vs. *A. naeslundii*:TAV - Control	-0,4717	-1,453 to 0,5101	No	ns	0,9977
S. sanguinis:TNZT - HF vs. *A. naeslundii*:TAV - CaP	-0,8667	-1,848 to 0,1151	No	ns	0,1920
***S. sanguinis*:TNZT - HF vs. *A. naeslundii*:TAV - CaPAg**	**-1,013**	**-1,995 to -0,03159**	**Yes**	*****	**0,0327**
S. sanguinis:TNZT - HF vs. *A. naeslundii*:TAV - HF	-0,3027	-1,332 to 0,7270	No	ns	>0,9999
S. sanguinis:TNZT - HF vs. *A. naeslundii*:TAV - HFAg	-0,09333	-1,075 to 0,8884	No	ns	>0,9999
***S. sanguinis*:TNZT - HF vs. *A. naeslundii*:TNZT - Control**	**-0,9983**	**-1,980 to -0,01659**	**Yes**	*****	**0,0401**
***S. sanguinis*:TNZT - HF vs. *A. naeslundii*:TNZT - CaP**	**-1,168**	**-2,150 to -0,1866**	**Yes**	******	**0,0031**
S. sanguinis:TNZT - HF vs. *A. naeslundii*:TNZT - CaPAg	-0,8633	-1,845 to 0,1184	No	ns	0,1986
S. sanguinis:TNZT - HF vs. *A. naeslundii*:TNZT - HF	-0,3533	-1,335 to 0,6284	No	ns	>0,9999
S. sanguinis:TNZT - HF vs. *A. naeslundii*:TNZT - HFAg	-0,6927	-1,722 to 0,3370	No	ns	0,7890
S. sanguinis:TNZT - HF vs. *P. gingivalis*:TAV - Control	-0,8433	-1,825 to 0,1384	No	ns	0,2414
***S. sanguinis*:TNZT - HF vs. *P. gingivalis*:TAV - CaP**	**-1,482**	**-2,463 to -0,4999**	**Yes**	********	**<0,0001**
***S. sanguinis*:TNZT - HF vs. *P. gingivalis*:TAV - CaPAg**	**-1,878**	**-2,860 to -0,8966**	**Yes**	********	**<0,0001**
***S. sanguinis*:TNZT - HF vs. *P. gingivalis*:TAV - HF**	**-1,213**	**-2,195 to -0,2316**	**Yes**	******	**0,0014**
***S. sanguinis*:TNZT - HF vs. *P. gingivalis*:TAV - HFAg**	**-1,198**	**-2,180 to -0,2166**	**Yes**	******	**0,0018**
S. sanguinis:TNZT - HF vs. *P. gingivalis*:TNZT - Control	-0,9527	-1,982 to 0,07700	No	ns	0,1227
***S. sanguinis*:TNZT - HF vs. *P. gingivalis*:TNZT - CaP**	**-2,152**	**-3,249 to -1,054**	**Yes**	********	**<0,0001**
***S. sanguinis*:TNZT - HF vs. *P. gingivalis*:TNZT - CaPAg**	**-2,135**	**-3,117 to -1,153**	**Yes**	********	**<0,0001**
***S. sanguinis*:TNZT - HF vs. *P. gingivalis*:TNZT - HF**	**-1,390**	**-2,372 to -0,4083**	**Yes**	********	**<0,0001**
***S. sanguinis*:TNZT - HF vs. *P. gingivalis*:TNZT - HFAg**	**-1,830**	**-2,812 to -0,8483**	**Yes**	********	**<0,0001**
***S. sanguinis*:TNZT - HFAg vs. *A. naeslundii*:TAV - Control**	**-1,025**	**-2,007 to -0,04326**	**Yes**	*****	**0,0278**
***S. sanguinis*:TNZT - HFAg vs. *A. naeslundii*:TAV - CaP**	**-1,420**	**-2,402 to -0,4383**	**Yes**	********	**<0,0001**
***S. sanguinis*:TNZT - HFAg vs. *A. naeslundii*:TAV - CaPAg**	**-1,567**	**-2,548 to -0,5849**	**Yes**	********	**<0,0001**
S. sanguinis:TNZT - HFAg vs. *A. naeslundii*:TAV - HF	-0,8560	-1,886 to 0,1737	No	ns	0,3087
S. sanguinis:TNZT - HFAg vs. *A. naeslundii*:TAV - HFAg	-0,6467	-1,628 to 0,3351	No	ns	0,8243
***S. sanguinis*:TNZT - HFAg vs. *A. naeslundii*:TNZT - Control**	**-1,552**	**-2,533 to -0,5699**	**Yes**	********	**<0,0001**
***S. sanguinis*:TNZT - HFAg vs. *A. naeslundii*:TNZT - CaP**	**-1,722**	**-2,703 to -0,7399**	**Yes**	********	**<0,0001**
***S. sanguinis*:TNZT - HFAg vs. *A. naeslundii*:TNZT - CaPAg**	**-1,417**	**-2,398 to -0,4349**	**Yes**	********	**<0,0001**
S. sanguinis:TNZT - HFAg vs. *A. naeslundii*:TNZT - HF	-0,9067	-1,888 to 0,07508	No	ns	0,1250
***S. sanguinis*:TNZT - HFAg vs. *A. naeslundii*:TNZT - HFAg**	**-1,246**	**-2,276 to -0,2163**	**Yes**	******	**0,0022**
***S. sanguinis*:TNZT - HFAg vs. *P. gingivalis*:TAV - Control**	**-1,397**	**-2,378 to -0,4149**	**Yes**	********	**<0,0001**
***S. sanguinis*:TNZT - HFAg vs. *P. gingivalis*:TAV - CaP**	**-2,035**	**-3,017 to -1,053**	**Yes**	********	**<0,0001**
***S. sanguinis*:TNZT - HFAg vs. *P. gingivalis*:TAV - CaPAg**	**-2,432**	**-3,413 to -1,450**	**Yes**	********	**<0,0001**
***S. sanguinis*:TNZT - HFAg vs. *P. gingivalis*:TAV - HF**	**-1,767**	**-2,748 to -0,7849**	**Yes**	********	**<0,0001**
***S. sanguinis*:TNZT - HFAg vs. *P. gingivalis*:TAV - HFAg**	**-1,752**	**-2,733 to -0,7699**	**Yes**	********	**<0,0001**
***S. sanguinis*:TNZT - HFAg vs. *P. gingivalis*:TNZT - Control**	**-1,506**	**-2,536 to -0,4763**	**Yes**	********	**<0,0001**
***S. sanguinis*:TNZT - HFAg vs. *P. gingivalis*:TNZT - CaP**	**-2,705**	**-3,803 to -1,607**	**Yes**	********	**<0,0001**
***S. sanguinis*:TNZT - HFAg vs. *P. gingivalis*:TNZT - CaPAg**	**-2,688**	**-3,670 to -1,707**	**Yes**	********	**<0,0001**
***S. sanguinis*:TNZT - HFAg vs. *P. gingivalis*:TNZT - HF**	**-1,943**	**-2,925 to -0,9616**	**Yes**	********	**<0,0001**
***S. sanguinis*:TNZT - HFAg vs. *P. gingivalis*:TNZT - HFAg**	**-2,383**	**-3,365 to -1,402**	**Yes**	********	**<0,0001**
A. naeslundii:TAV - Control vs. *A. naeslundii*:TAV - CaP	-0,3950	-1,377 to 0,5867	No	ns	>0,9999
A. naeslundii:TAV - Control vs. *A. naeslundii*:TAV - CaPAg	-0,5417	-1,523 to 0,4401	No	ns	0,9776
A. naeslundii:TAV - Control vs. *A. naeslundii*:TAV - HF	0,1690	-0,8607 to 1,199	No	ns	>0,9999
A. naeslundii:TAV - Control vs. *A. naeslundii*:TAV - HFAg	0,3783	-0,6034 to 1,360	No	ns	>0,9999
A. naeslundii:TAV - Control vs. *A. naeslundii*:TNZT - Control	-0,5267	-1,508 to 0,4551	No	ns	0,9852
A. naeslundii:TAV - Control vs. *A. naeslundii*:TNZT - CaP	-0,6967	-1,678 to 0,2851	No	ns	0,6828
A. naeslundii:TAV - Control vs. *A. naeslundii*:TNZT - CaPAg	-0,3917	-1,373 to 0,5901	No	ns	>0,9999
A. naeslundii:TAV - Control vs. *A. naeslundii*:TNZT - HF	0,1183	-0,8634 to 1,100	No	ns	>0,9999
A. naeslundii:TAV - Control vs. *A. naeslundii*:TNZT - HFAg	-0,2210	-1,251 to 0,8087	No	ns	>0,9999
A. naeslundii:TAV - Control vs. *P. gingivalis*:TAV - Control	-0,3717	-1,353 to 0,6101	No	ns	>0,9999
***A. naeslundii*:TAV - Control vs. *P. gingivalis*:TAV - CaP**	**-1,010**	**-1,992 to -0,02826**	**Yes**	*****	**0,0342**
***A. naeslundii*:TAV - Control vs. *P. gingivalis*:TAV - CaPAg**	**-1,407**	**-2,388 to -0,4249**	**Yes**	********	**<0,0001**
A. naeslundii:TAV - Control vs. *P. gingivalis*:TAV - HF	-0,7417	-1,723 to 0,2401	No	ns	0,5361
A. naeslundii:TAV - Control vs. *P. gingivalis*:TAV - HFAg	-0,7267	-1,708 to 0,2551	No	ns	0,5857
A. naeslundii:TAV - Control vs. *P. gingivalis*:TNZT - Control	-0,4810	-1,511 to 0,5487	No	ns	0,9986
***A. naeslundii*:TAV - Control vs. *P. gingivalis*:TNZT - CaP**	**-1,680**	**-2,778 to -0,5824**	**Yes**	********	**<0,0001**
***A. naeslundii*:TAV - Control vs. *P. gingivalis*:TNZT - CaPAg**	**-1,663**	**-2,645 to -0,6816**	**Yes**	********	**<0,0001**
A. naeslundii:TAV - Control vs. *P. gingivalis*:TNZT - HF	-0,9183	-1,900 to 0,06341	No	ns	0,1094
***A. naeslundii*:TAV - Control vs. *P. gingivalis*:TNZT - HFAg**	**-1,358**	**-2,340 to -0,3766**	**Yes**	*******	**0,0001**
A. naeslundii:TAV - CaP vs. *A. naeslundii*:TAV - CaPAg	-0,1467	-1,128 to 0,8351	No	ns	>0,9999
A. naeslundii:TAV - CaP vs. *A. naeslundii*:TAV - HF	0,5640	-0,4657 to 1,594	No	ns	0,9798
A. naeslundii:TAV - CaP vs. *A. naeslundii*:TAV - HFAg	0,7733	-0,2084 to 1,755	No	ns	0,4337
A. naeslundii:TAV - CaP vs. *A. naeslundii*:TNZT - Control	-0,1317	-1,113 to 0,8501	No	ns	>0,9999
A. naeslundii:TAV - CaP vs. *A. naeslundii*:TNZT - CaP	-0,3017	-1,283 to 0,6801	No	ns	>0,9999
A. naeslundii:TAV - CaP vs. *A. naeslundii*:TNZT - CaPAg	0,003333	-0,9784 to 0,9851	No	ns	>0,9999
A. naeslundii:TAV - CaP vs. *A. naeslundii*:TNZT - HF	0,5133	-0,4684 to 1,495	No	ns	0,9900
A. naeslundii:TAV - CaP vs. *A. naeslundii*:TNZT - HFAg	0,1740	-0,8557 to 1,204	No	ns	>0,9999

A. naeslundii:TAV - CaP vs. *P. gingivalis*:TAV - Control	0,02333	-0,9584 to 1,005	No	ns	>0,9999
A. naeslundii:TAV - CaP vs. *P. gingivalis*:TAV - CaP	-0,6150	-1,597 to 0,3667	No	ns	0,8922
**_A. naeslundii_:TAV - CaP vs. _P. gingivalis_:TAV - CaPAg**	**-1,012**	**-1,993 to -0,02992**	**Yes**	*****	**0,0334**
A. naeslundii:TAV - CaP vs. *P. gingivalis*:TAV - HF	-0,3467	-1,328 to 0,6351	No	ns	>0,9999
A. naeslundii:TAV - CaP vs. *P. gingivalis*:TAV - HFAg	-0,3317	-1,313 to 0,6501	No	ns	>0,9999
A. naeslundii:TAV - CaP vs. *P. gingivalis*:TNZT - Control	-0,08600	-1,116 to 0,9437	No	ns	>0,9999
**_A. naeslundii_:TAV - CaP vs. _P. gingivalis_:TNZT - CaP**	**-1,285**	**-2,383 to -0,1874**	**Yes**	******	**0,0042**
**_A. naeslundii_:TAV - CaP vs. _P. gingivalis_:TNZT - CaPAg**	**-1,268**	**-2,250 to -0,2866**	**Yes**	*******	**0,0005**
A. naeslundii:TAV - CaP vs. *P. gingivalis*:TNZT - HF	-0,5233	-1,505 to 0,4584	No	ns	0,9865
A. naeslundii:TAV - CaP vs. *P. gingivalis*:TNZT - HFAg	-0,9633	-1,945 to 0,01841	No	ns	0,0634
A. naeslundii:TAV - CaPAg vs. *A. naeslundii*:TAV - HF	0,7107	-0,3190 to 1,740	No	ns	0,7408
A. naeslundii:TAV - CaPAg vs. *A. naeslundii*:TAV - HFAg	0,9200	-0,06174 to 1,902	No	ns	0,1073
A. naeslundii:TAV - CaPAg vs. *A. naeslundii*:TNZT - Control	0,01500	-0,9667 to 0,9967	No	ns	>0,9999
A. naeslundii:TAV - CaPAg vs. *A. naeslundii*:TNZT - CaP	-0,1550	-1,137 to 0,8267	No	ns	>0,9999
A. naeslundii:TAV - CaPAg vs. *A. naeslundii*:TNZT - CaPAg	0,1500	-0,8317 to 1,132	No	ns	>0,9999
A. naeslundii:TAV - CaPAg vs. *A. naeslundii*:TNZT - HF	0,6600	-0,3217 to 1,642	No	ns	0,7902
A. naeslundii:TAV - CaPAg vs. *A. naeslundii*:TNZT - HFAg	0,3207	-0,7090 to 1,350	No	ns	>0,9999
A. naeslundii:TAV - CaPAg vs. *P. gingivalis*:TAV - Control	0,1700	-0,8117 to 1,152	No	ns	>0,9999
A. naeslundii:TAV - CaPAg vs. *P. gingivalis*:TAV - CaP	-0,4683	-1,450 to 0,5134	No	ns	0,9979
A. naeslundii:TAV - CaPAg vs. *P. gingivalis*:TAV - CaPAg	-0,8650	-1,847 to 0,1167	No	ns	0,1952
A. naeslundii:TAV - CaPAg vs. *P. gingivalis*:TAV - HF	-0,2000	-1,182 to 0,7817	No	ns	>0,9999
A. naeslundii:TAV - CaPAg vs. *P. gingivalis*:TAV - HFAg	-0,1850	-1,167 to 0,7967	No	ns	>0,9999
A. naeslundii:TAV - CaPAg vs. *P. gingivalis*:TNZT - Control	0,06067	-0,9690 to 1,090	No	ns	>0,9999
**_A. naeslundii_:TAV - CaPAg vs. _P. gingivalis_:TNZT - CaP**	**-1,138**	**-2,236 to -0,04071**	**Yes**	*****	**0,0306**
**_A. naeslundii_:TAV - CaPAg vs. _P. gingivalis_:TNZT - CaPAg**	**-1,122**	**-2,103 to -0,1399**	**Yes**	******	**0,0065**
A. naeslundii:TAV - CaPAg vs. *P. gingivalis*:TNZT - HF	-0,3767	-1,358 to 0,6051	No	ns	>0,9999
A. naeslundii:TAV - CaPAg vs. *P. gingivalis*:TNZT - HFAg	-0,8167	-1,798 to 0,1651	No	ns	0,3074
A. naeslundii:TAV - HF vs. *A. naeslundii*:TAV - HFAg	0,2093	-0,8203 to 1,239	No	ns	>0,9999
A. naeslundii:TAV - HF vs. *A. naeslundii*:TNZT - Control	-0,6957	-1,725 to 0,3340	No	ns	0,7813
A. naeslundii:TAV - HF vs. *A. naeslundii*:TNZT - CaP	-0,8657	-1,895 to 0,1640	No	ns	0,2847
A. naeslundii:TAV - HF vs. *A. naeslundii*:TNZT - CaPAg	-0,5607	-1,590 to 0,4690	No	ns	0,9815
A. naeslundii:TAV - HF vs. *A. naeslundii*:TNZT - HF	-0,05067	-1,080 to 0,9790	No	ns	>0,9999
A. naeslundii:TAV - HF vs. *A. naeslundii*:TNZT - HFAg	-0,3900	-1,465 to 0,6854	No	ns	>0,9999
A. naeslundii:TAV - HF vs. *P. gingivalis*:TAV - Control	-0,5407	-1,570 to 0,4890	No	ns	0,9893
**_A. naeslundii_:TAV - HF vs. _P. gingivalis_:TAV - CaP**	**-1,179**	**-2,209 to -0,1493**	**Yes**	******	**0,0063**
**_A. naeslundii_:TAV - HF vs. _P. gingivalis_:TAV - CaPAg**	**-1,576**	**-2,605 to -0,5460**	**Yes**	********	**<0,0001**
A. naeslundii:TAV - HF vs. *P. gingivalis*:TAV - HF	-0,9107	-1,940 to 0,1190	No	ns	0,1888
A. naeslundii:TAV - HF vs. *P. gingivalis*:TAV - HFAg	-0,8957	-1,925 to 0,1340	No	ns	0,2179
A. naeslundii:TAV - HF vs. *P. gingivalis*:TNZT - Control	-0,6500	-1,725 to 0,4254	No	ns	0,9273
**_A. naeslundii_:TAV - HF vs. _P. gingivalis_:TNZT - CaP**	**-1,849**	**-2,990 to -0,7083**	**Yes**	********	**<0,0001**
**_A. naeslundii_:TAV - HF vs. _P. gingivalis_:TNZT - CaPAg**	**-1,832**	**-2,862 to -0,8027**	**Yes**	********	**<0,0001**
**_A. naeslundii_:TAV - HF vs. _P. gingivalis_:TNZT - HF**	**-1,087**	**-2,117 to -0,05767**	**Yes**	*****	**0,0235**
**_A. naeslundii_:TAV - HF vs. _P. gingivalis_:TNZT - HFAg**	**-1,527**	**-2,557 to -0,4977**	**Yes**	********	**<0,0001**
A. naeslundii:TAV - HFAg vs. *A. naeslundii*:TNZT - Control	-0,9050	-1,887 to 0,07674	No	ns	0,1274
**_A. naeslundii_:TAV - HFAg vs. _A. naeslundii_:TNZT - CaP**	**-1,075**	**-2,057 to -0,09326**	**Yes**	*****	**0,0134**
A. naeslundii:TAV - HFAg vs. *A. naeslundii*:TNZT - CaPAg	-0,7700	-1,752 to 0,2117	No	ns	0,4442
A. naeslundii:TAV - HFAg vs. *A. naeslundii*:TNZT - HF	-0,2600	-1,242 to 0,7217	No	ns	>0,9999
A. naeslundii:TAV - HFAg vs. *A. naeslundii*:TNZT - HFAg	-0,5993	-1,629 to 0,4303	No	ns	0,9539
A. naeslundii:TAV - HFAg vs. *P. gingivalis*:TAV - Control	-0,7500	-1,732 to 0,2317	No	ns	0,5087
**_A. naeslundii_:TAV - HFAg vs. _P. gingivalis_:TAV - CaP**	**-1,388**	**-2,370 to -0,4066**	**Yes**	********	**<0,0001**
**_A. naeslundii_:TAV - HFAg vs. _P. gingivalis_:TAV - CaPAg**	**-1,785**	**-2,767 to -0,8033**	**Yes**	********	**<0,0001**
**_A. naeslundii_:TAV - HFAg vs. _P. gingivalis_:TAV - HF**	**-1,120**	**-2,102 to -0,1383**	**Yes**	******	**0,0067**
**_A. naeslundii_:TAV - HFAg vs. _P. gingivalis_:TAV - HFAg**	**-1,105**	**-2,087 to -0,1233**	**Yes**	******	**0,0085**
A. naeslundii:TAV - HFAg vs. *P. gingivalis*:TNZT - Control	-0,8593	-1,889 to 0,1703	No	ns	0,3003
**_A. naeslundii_:TAV - HFAg vs. _P. gingivalis_:TNZT - CaP**	**-2,058**	**-3,156 to -0,9607**	**Yes**	********	**<0,0001**
**_A. naeslundii_:TAV - HFAg vs. _P. gingivalis_:TNZT - CaPAg**	**-2,042**	**-3,023 to -1,060**	**Yes**	********	**<0,0001**
**_A. naeslundii_:TAV - HFAg vs. _P. gingivalis_:TNZT - HF**	**-1,297**	**-2,278 to -0,3149**	**Yes**	*******	**0,0003**
**_A. naeslundii_:TAV - HFAg vs. _P. gingivalis_:TNZT - HFAg**	**-1,737**	**-2,718 to -0,7549**	**Yes**	********	**<0,0001**
A. naeslundii:TNZT - Control vs. *A. naeslundii*:TNZT - CaP	-0,1700	-1,152 to 0,8117	No	ns	>0,9999
A. naeslundii:TNZT - Control vs. *A. naeslundii*:TNZT - CaPAg	0,1350	-0,8467 to 1,117	No	ns	>0,9999
A. naeslundii:TNZT - Control vs. *A. naeslundii*:TNZT - HF	0,6450	-0,3367 to 1,627	No	ns	0,8283
A. naeslundii:TNZT - Control vs. *A. naeslundii*:TNZT - HFAg	0,3057	-0,7240 to 1,335	No	ns	>0,9999
A. naeslundii:TNZT - Control vs. *P. gingivalis*:TAV - Control	0,1550	-0,8267 to 1,137	No	ns	>0,9999
A. naeslundii:TNZT - Control vs. *P. gingivalis*:TAV - CaP	-0,4833	-1,465 to 0,4984	No	ns	0,9964
A. naeslundii:TNZT - Control vs. *P. gingivalis*:TAV - CaPAg	-0,8800	-1,862 to 0,1017	No	ns	0,1672
A. naeslundii:TNZT - Control vs. *P. gingivalis*:TAV - HF	-0,2150	-1,197 to 0,7667	No	ns	>0,9999
A. naeslundii:TNZT - Control vs. *P. gingivalis*:TAV - HFAg	-0,2000	-1,182 to 0,7817	No	ns	>0,9999
A. naeslundii:TNZT - Control vs. *P. gingivalis*:TNZT - Control	0,04567	-0,9840 to 1,075	No	ns	>0,9999
**_A. naeslundii_:TNZT - Control vs. _P. gingivalis_:TNZT - CaP**	**-1,153**	**-2,251 to -0,05571**	**Yes**	*****	**0,0253**
**_A. naeslundii_:TNZT - Control vs. _P. gingivalis_:TNZT - CaPAg**	**-1,137**	**-2,118 to -0,1549**	**Yes**	******	**0,0051**
A. naeslundii:TNZT - Control vs. *P. gingivalis*:TNZT - HF	-0,3917	-1,373 to 0,5901	No	ns	>0,9999
A. naeslundii:TNZT - Control vs. *P. gingivalis*:TNZT - HFAg	-0,8317	-1,813 to 0,1501	No	ns	0,2690

A. naeslundii:TNZT - CaP vs. *A. naeslundii*:TNZT - CaPAg	0,3050	-0,6767 to 1,287	No	ns	>0,9999
A. naeslundii:TNZT - CaP vs. *A. naeslundii*:TNZT - HF	0,8150	-0,1667 to 1,797	No	ns	0,3118
A. naeslundii:TNZT - CaP vs. *A. naeslundii*:TNZT - HFAg	0,4757	-0,5540 to 1,505	No	ns	0,9989
A. naeslundii:TNZT - CaP vs. *P. gingivalis*:TAV - Control	0,3250	-0,6567 to 1,307	No	ns	>0,9999
A. naeslundii:TNZT - CaP vs. *P. gingivalis*:TAV - CaP	-0,3133	-1,295 to 0,6684	No	ns	>0,9999
A. naeslundii:TNZT - CaP vs. *P. gingivalis*:TAV - CaPAg	-0,7100	-1,692 to 0,2717	No	ns	0,6402
A. naeslundii:TNZT - CaP vs. *P. gingivalis*:TAV - HF	-0,04500	-1,027 to 0,9367	No	ns	>0,9999
A. naeslundii:TNZT - CaP vs. *P. gingivalis*:TAV - HFAg	-0,03000	-1,012 to 0,9517	No	ns	>0,9999
A. naeslundii:TNZT - CaP vs. *P. gingivalis*:TNZT - Control	0,2157	-0,8140 to 1,245	No	ns	>0,9999
A. naeslundii:TNZT - CaP vs. *P. gingivalis*:TNZT - CaP	-0,9833	-2,081 to 0,1143	No	ns	0,1681
A. naeslundii:TNZT - CaP vs. *P. gingivalis*:TNZT - CaPAg	-0,9667	-1,948 to 0,01508	No	ns	0,0608
A. naeslundii:TNZT - CaP vs. *P. gingivalis*:TNZT - HF	-0,2217	-1,203 to 0,7601	No	ns	>0,9999
A. naeslundii:TNZT - CaP vs. *P. gingivalis*:TNZT - HFAg	-0,6617	-1,643 to 0,3201	No	ns	0,7857
A. naeslundii:TNZT - CaPAg vs. *A. naeslundii*:TNZT - HF	0,5100	-0,4717 to 1,492	No	ns	0,9910
A. naeslundii:TNZT - CaPAg vs. *A. naeslundii*:TNZT - HFAg	0,1707	-0,8590 to 1,200	No	ns	>0,9999
A. naeslundii:TNZT - CaPAg vs. *P. gingivalis*:TAV - Control	0,02000	-0,9617 to 1,002	No	ns	>0,9999
A. naeslundii:TNZT - CaPAg vs. *P. gingivalis*:TAV - CaP	-0,6183	-1,600 to 0,3634	No	ns	0,8860
***A. naeslundii*:TNZT - CaPAg vs. *P. gingivalis*:TAV - CaPAg**	**-1,015**	**-1,997 to -0,03326**	**Yes**	*****	**0,0319**
A. naeslundii:TNZT - CaPAg vs. *P. gingivalis*:TAV - HF	-0,3500	-1,332 to 0,6317	No	ns	>0,9999
A. naeslundii:TNZT - CaPAg vs. *P. gingivalis*:TAV - HFAg	-0,3350	-1,317 to 0,6467	No	ns	>0,9999
A. naeslundii:TNZT - CaPAg vs. *P. gingivalis*:TNZT - Control	-0,08933	-1,119 to 0,9403	No	ns	>0,9999
***A. naeslundii*:TNZT - CaPAg vs. *P. gingivalis*:TNZT - CaP**	**-1,288**	**-2,386 to -0,1907**	**Yes**	******	**0,0040**
***A. naeslundii*:TNZT - CaPAg vs. *P. gingivalis*:TNZT - CaPAg**	**-1,272**	**-2,253 to -0,2899**	**Yes**	*******	**0,0005**
A. naeslundii:TNZT - CaPAg vs. *P. gingivalis*:TNZT - HF	-0,5267	-1,508 to 0,4551	No	ns	0,9852
A. naeslundii:TNZT - CaPAg vs. *P. gingivalis*:TNZT - HFAg	-0,9667	-1,948 to 0,01508	No	ns	0,0608
A. naeslundii:TNZT - HF vs. *A. naeslundii*:TNZT - HFAg	-0,3393	-1,369 to 0,6903	No	ns	>0,9999
A. naeslundii:TNZT - HF vs. *P. gingivalis*:TAV - Control	-0,4900	-1,472 to 0,4917	No	ns	0,9954
***A. naeslundii*:TNZT - HF vs. *P. gingivalis*:TAV - CaP**	**-1,128**	**-2,110 to -0,1466**	**Yes**	******	**0,0059**
***A. naeslundii*:TNZT - HF vs. *P. gingivalis*:TAV - CaPAg**	**-1,525**	**-2,507 to -0,5433**	**Yes**	********	**<0,0001**
A. naeslundii:TNZT - HF vs. *P. gingivalis*:TAV - HF	-0,8600	-1,842 to 0,1217	No	ns	0,2053
A. naeslundii:TNZT - HF vs. *P. gingivalis*:TAV - HFAg	-0,8450	-1,827 to 0,1367	No	ns	0,2376
A. naeslundii:TNZT - HF vs. *P. gingivalis*:TNZT - Control	-0,5993	-1,629 to 0,4303	No	ns	0,9539
***A. naeslundii*:TNZT - HF vs. *P. gingivalis*:TNZT - CaP**	**-1,798**	**-2,896 to -0,7007**	**Yes**	********	**<0,0001**
***A. naeslundii*:TNZT - HF vs. *P. gingivalis*:TNZT - CaPAg**	**-1,782**	**-2,763 to -0,7999**	**Yes**	********	**<0,0001**
***A. naeslundii*:TNZT - HF vs. *P. gingivalis*:TNZT - HF**	**-1,037**	**-2,018 to -0,05492**	**Yes**	*****	**0,0236**
***A. naeslundii*:TNZT - HF vs. *P. gingivalis*:TNZT - HFAg**	**-1,477**	**-2,458 to -0,4949**	**Yes**	********	**<0,0001**
A. naeslundii:TNZT - HFAg vs. *P. gingivalis*:TAV - Control	-0,1507	-1,180 to 0,8790	No	ns	>0,9999
A. naeslundii:TNZT - HFAg vs. *P. gingivalis*:TAV - CaP	-0,7890	-1,819 to 0,2407	No	ns	0,5013
***A. naeslundii*:TNZT - HFAg vs. *P. gingivalis*:TAV - CaPAg**	**-1,186**	**-2,215 to -0,1560**	**Yes**	******	**0,0057**
A. naeslundii:TNZT - HFAg vs. *P. gingivalis*:TAV - HF	-0,5207	-1,550 to 0,5090	No	ns	0,9942
A. naeslundii:TNZT - HFAg vs. *P. gingivalis*:TAV - HFAg	-0,5057	-1,535 to 0,5240	No	ns	0,9965
A. naeslundii:TNZT - HFAg vs. *P. gingivalis*:TNZT - Control	-0,2600	-1,335 to 0,8154	No	ns	>0,9999
***A. naeslundii*:TNZT - HFAg vs. *P. gingivalis*:TNZT - CaP**	**-1,459**	**-2,600 to -0,3183**	**Yes**	*******	**0,0007**
***A. naeslundii*:TNZT - HFAg vs. *P. gingivalis*:TNZT - CaPAg**	**-1,442**	**-2,472 to -0,4127**	**Yes**	********	**<0,0001**
A. naeslundii:TNZT - HFAg vs. *P. gingivalis*:TNZT - HF	-0,6973	-1,727 to 0,3323	No	ns	0,7769
***A. naeslundii*:TNZT - HFAg vs. *P. gingivalis*:TNZT - HFAg**	**-1,137**	**-2,167 to -0,1077**	**Yes**	*****	**0,0117**
P. gingivalis:TAV - Control vs. *P. gingivalis*:TAV - CaP	-0,6383	-1,620 to 0,3434	No	ns	0,8440
***P. gingivalis*:TAV - Control vs. *P. gingivalis*:TAV - CaPAg**	**-1,035**	**-2,017 to -0,05326**	**Yes**	*****	**0,0241**
P. gingivalis:TAV - Control vs. *P. gingivalis*:TAV - HF	-0,3700	-1,352 to 0,6117	No	ns	>0,9999
P. gingivalis:TAV - Control vs. *P. gingivalis*:TAV - HFAg	-0,3550	-1,337 to 0,6267	No	ns	>0,9999
P. gingivalis:TAV - Control vs. *P. gingivalis*:TNZT - Control	-0,1093	-1,139 to 0,9203	No	ns	>0,9999
***P. gingivalis*:TAV - Control vs. *P. gingivalis*:TNZT - CaP**	**-1,308**	**-2,406 to -0,2107**	**Yes**	******	**0,0030**
***P. gingivalis*:TAV - Control vs. *P. gingivalis*:TNZT - CaPAg**	**-1,292**	**-2,273 to -0,3099**	**Yes**	*******	**0,0004**
P. gingivalis:TAV - Control vs. *P. gingivalis*:TNZT - HF	-0,5467	-1,528 to 0,4351	No	ns	0,9745
***P. gingivalis*:TAV - Control vs. *P. gingivalis*:TNZT - HFAg**	**-0,9867**	**-1,968 to -0,004922**	**Yes**	*****	**0,0469**
P. gingivalis:TAV - CaP vs. *P. gingivalis*:TAV - CaPAg	-0,3967	-1,378 to 0,5851	No	ns	>0,9999
P. gingivalis:TAV - CaP vs. *P. gingivalis*:TAV - HF	0,2683	-0,7134 to 1,250	No	ns	>0,9999
P. gingivalis:TAV - CaP vs. *P. gingivalis*:TAV - HFAg	0,2833	-0,6984 to 1,265	No	ns	>0,9999
P. gingivalis:TAV - CaP vs. *P. gingivalis*:TNZT - Control	0,5290	-0,5007 to 1,559	No	ns	0,9925
P. gingivalis:TAV - CaP vs. *P. gingivalis*:TNZT - CaP	-0,6700	-1,768 to 0,4276	No	ns	0,9186
P. gingivalis:TAV - CaP vs. *P. gingivalis*:TNZT - CaPAg	-0,6533	-1,635 to 0,3284	No	ns	0,8076
P. gingivalis:TAV - CaP vs. *P. gingivalis*:TNZT - HF	0,09167	-0,8901 to 1,073	No	ns	>0,9999
P. gingivalis:TAV - CaP vs. *P. gingivalis*:TNZT - HFAg	-0,3483	-1,330 to 0,6334	No	ns	>0,9999
P. gingivalis:TAV - CaPAg vs. *P. gingivalis*:TAV - HF	0,6650	-0,3167 to 1,647	No	ns	0,7766
P. gingivalis:TAV - CaPAg vs. *P. gingivalis*:TAV - HFAg	0,6800	-0,3017 to 1,662	No	ns	0,7337
P. gingivalis:TAV - CaPAg vs. *P. gingivalis*:TNZT - Control	0,9257	-0,1040 to 1,955	No	ns	0,1627
P. gingivalis:TAV - CaPAg vs. *P. gingivalis*:TNZT - CaP	-0,2733	-1,371 to 0,8243	No	ns	>0,9999
P. gingivalis:TAV - CaPAg vs. *P. gingivalis*:TNZT - CaPAg	-0,2567	-1,238 to 0,7251	No	ns	>0,9999
P. gingivalis:TAV - CaPAg vs. *P. gingivalis*:TNZT - HF	0,4883	-0,4934 to 1,470	No	ns	0,9957
P. gingivalis:TAV - CaPAg vs. *P. gingivalis*:TNZT - HFAg	0,04833	-0,9334 to 1,030	No	ns	>0,9999
P. gingivalis:TAV - HF vs. *P. gingivalis*:TAV - HFAg	0,01500	-0,9667 to 0,9967	No	ns	>0,9999
P. gingivalis:TAV - HF vs. *P. gingivalis*:TNZT - Control	0,2607	-0,7690 to 1,290	No	ns	>0,9999

P. gingivalis:TAV - HF vs. *P. gingivalis*:TNZT - CaP	-0,9383	-2,036 to 0,1593	No	ns	0,2508
P. gingivalis:TAV - HF vs. *P. gingivalis*:TNZT - CaPAg	-0,9217	-1,903 to 0,06008	No	ns	0,1053
P. gingivalis:TAV - HF vs. *P. gingivalis*:TNZT - HF	-0,1767	-1,158 to 0,8051	No	ns	>0,9999
P. gingivalis:TAV - HF vs. *P. gingivalis*:TNZT - HFAg	-0,6167	-1,598 to 0,3651	No	ns	0,8891
P. gingivalis:TAV - HFAg vs. *P. gingivalis*:TNZT - Control	0,2457	-0,7840 to 1,275	No	ns	>0,9999
P. gingivalis:TAV - HFAg vs. *P. gingivalis*:TNZT - CaP	-0,9533	-2,051 to 0,1443	No	ns	0,2206
P. gingivalis:TAV - HFAg vs. *P. gingivalis*:TNZT - CaPAg	-0,9367	-1,918 to 0,04508	No	ns	0,0881
P. gingivalis:TAV - HFAg vs. *P. gingivalis*:TNZT - HF	-0,1917	-1,173 to 0,7901	No	ns	>0,9999
P. gingivalis:TAV - HFAg vs. *P. gingivalis*:TNZT - HFAg	-0,6317	-1,613 to 0,3501	No	ns	0,8589
***P. gingivalis*:TNZT - Control vs. *P. gingivalis*:TNZT - CaP**	**-1,199**	**-2,340 to -0,05832**	**Yes**	*****	**0,0252**
***P. gingivalis*:TNZT - Control vs. *P. gingivalis*:TNZT - CaPAg**	**-1,182**	**-2,212 to -0,1527**	**Yes**	******	**0,0060**
P. gingivalis:TNZT - Control vs. *P. gingivalis*:TNZT - HF	-0,4373	-1,467 to 0,5923	No	ns	0,9998
P. gingivalis:TNZT - Control vs. *P. gingivalis*:TNZT - HFAg	-0,8773	-1,907 to 0,1523	No	ns	0,2573
P. gingivalis:TNZT - CaP vs. *P. gingivalis*:TNZT - CaPAg	0,01667	-1,081 to 1,114	No	ns	>0,9999
P. gingivalis:TNZT - CaP vs. *P. gingivalis*:TNZT - HF	0,7617	-0,3360 to 1,859	No	ns	0,7300
P. gingivalis:TNZT - CaP vs. *P. gingivalis*:TNZT - HFAg	0,3217	-0,7760 to 1,419	No	ns	>0,9999
P. gingivalis:TNZT - CaPAg vs. *P. gingivalis*:TNZT - HF	0,7450	-0,2367 to 1,727	No	ns	0,5251
P. gingivalis:TNZT - CaPAg vs. *P. gingivalis*:TNZT - HFAg	0,3050	-0,6767 to 1,287	No	ns	>0,9999
P. gingivalis:TNZT - HF vs. *P. gingivalis*:TNZT - HFAg	-0,4400	-1,422 to 0,5417	No	ns	0,9994

Table S2. Statistical analysis of the bacterial biofilm data.

Table Analyzed	Grouped:				
Two-way ANOVA	Ordinary				
Alpha	0,05				
Source of Variation	% of total variation	P value	P value summary	Significant?	
Interaction	5,578	0,7982	ns	No	
Species	41,05	<0,0001	****	Yes	
Surface	2,052	0,5811	ns	No	
ANOVA table	SS (Type III)	DF	MS	F (DFn, DFd)	P value
Interaction	18,58	27	0,6883	F (27, 191) = 0,7601	P=0,7982
Species	136,7	3	45,58	F (3, 191) = 50,34	P<0,0001
Surface	6,837	9	0,7597	F (9, 191) = 0,8390	P=0,5811
Residual	173,0	191	0,9055		
Data summary					
Number of columns (Surface)	10				
Number of rows (Species)	4				
Number of values	231				

Multiple comparisons

Compare cell means regardless of rows and columns					
Number of families	1				
Number of comparisons per family	780				
Alpha	0,05				
Tukey's multiple comparisons test	Predicted (LS) mean diff,	95,00% CI of diff,	Below threshold?	Summary	Adjusted P Value
S. gordonii:TAV - Control vs. *S. gordonii*:TAV - CaP	0,6583	-1,513 to 2,830	No	ns	>0,9999
S. gordonii:TAV - Control vs. *S. gordonii*:TAV - CaPAg	0,1467	-2,025 to 2,318	No	ns	>0,9999
S. gordonii:TAV - Control vs. *S. gordonii*:TAV - HF	0,1200	-2,051 to 2,291	No	ns	>0,9999
S. gordonii:TAV - Control vs. *S. gordonii*:TAV - HFAg	0,3467	-1,825 to 2,518	No	ns	>0,9999
S. gordonii:TAV - Control vs. *S. gordonii*:TNZT - Control	-0,08000	-2,251 to 2,091	No	ns	>0,9999
S. gordonii:TAV - Control vs. *S. gordonii*:TNZT - CaP	1,058	-1,220 to 3,335	No	ns	0,9987
S. gordonii:TAV - Control vs. *S. gordonii*:TNZT - CaPAg	-0,04333	-2,215 to 2,128	No	ns	>0,9999
S. gordonii:TAV - Control vs. *S. gordonii*:TNZT - HF	0,3983	-1,773 to 2,570	No	ns	>0,9999
S. gordonii:TAV - Control vs. *S. gordonii*:TNZT - HFAg	0,2600	-1,911 to 2,431	No	ns	>0,9999
S. gordonii:TAV - Control vs. *S. sanguinis*:TAV - Control	-0,7000	-2,871 to 1,471	No	ns	>0,9999
S. gordonii:TAV - Control vs. *S. sanguinis*:TAV - CaP	-0,9500	-3,121 to 1,221	No	ns	0,9996
S. gordonii:TAV - Control vs. *S. sanguinis*:TAV - CaPAg	-0,7767	-2,948 to 1,395	No	ns	>0,9999
S. gordonii:TAV - Control vs. *S. sanguinis*:TAV - HF	-0,9967	-3,168 to 1,175	No	ns	0,9990
S. gordonii:TAV - Control vs. *S. sanguinis*:TAV - HFAg	-0,7350	-2,906 to 1,436	No	ns	>0,9999
S. gordonii:TAV - Control vs. *S. sanguinis*:TNZT - Control	-0,7500	-2,921 to 1,421	No	ns	>0,9999
S. gordonii:TAV - Control vs. *S. sanguinis*:TNZT - CaP	-1,615	-3,786 to 0,5564	No	ns	0,5735
S. gordonii:TAV - Control vs. *S. sanguinis*:TNZT - CaPAg	-1,207	-3,378 to 0,9647	No	ns	0,9750
S. gordonii:TAV - Control vs. *S. sanguinis*:TNZT - HF	-0,9763	-3,254 to 1,301	No	ns	0,9998
S. gordonii:TAV - Control vs. *S. sanguinis*:TNZT - HFAg	-1,108	-3,386 to 1,169	No	ns	0,9970
S. gordonii:TAV - Control vs. *A. naeslundii*:TAV - Control	0,3017	-1,870 to 2,473	No	ns	>0,9999
S. gordonii:TAV - Control vs. *A. naeslundii*:TAV - CaP	1,202	-1,226 to 3,629	No	ns	0,9960
S. gordonii:TAV - Control vs. *A. naeslundii*:TAV - CaPAg	0,2450	-1,926 to 2,416	No	ns	>0,9999
S. gordonii:TAV - Control vs. *A. naeslundii*:TAV - HF	0,2600	-1,911 to 2,431	No	ns	>0,9999
S. gordonii:TAV - Control vs. *A. naeslundii*:TAV - HFAg	0,4567	-1,715 to 2,628	No	ns	>0,9999
S. gordonii:TAV - Control vs. *A. naeslundii*:TNZT - Control	0,2183	-1,953 to 2,390	No	ns	>0,9999
S. gordonii:TAV - Control vs. *A. naeslundii*:TNZT - CaP	0,3017	-1,870 to 2,473	No	ns	>0,9999
S. gordonii:TAV - Control vs. *A. naeslundii*:TNZT - CaPAg	0,1233	-2,048 to 2,295	No	ns	>0,9999
S. gordonii:TAV - Control vs. *A. naeslundii*:TNZT - HF	0,6717	-1,500 to 2,843	No	ns	>0,9999
S. gordonii:TAV - Control vs. *A. naeslundii*:TNZT - HFAg	-0,04583	-2,474 to 2,382	No	ns	>0,9999
S. gordonii:TAV - Control vs. *P. gingivalis*:TAV - Control	-0,5317	-2,703 to 1,640	No	ns	>0,9999
S. gordonii:TAV - Control vs. *P. gingivalis*:TAV - CaP	-1,687	-3,858 to 0,4847	No	ns	0,4674
S. gordonii:TAV - Control vs. *P. gingivalis*:TAV - CaPAg	-1,795	-3,966 to 0,3764	No	ns	0,3208
S. gordonii:TAV - Control vs. *P. gingivalis*:TAV - HF	-0,9667	-3,138 to 1,205	No	ns	0,9995
S. gordonii:TAV - Control vs. *P. gingivalis*:TAV - HFAg	-1,476	-3,754 to 0,8010	No	ns	0,8479
S. gordonii:TAV - Control vs. *P. gingivalis*:TNZT - Control	-1,692	-3,863 to 0,4797	No	ns	0,4601
S. gordonii:TAV - Control vs. *P. gingivalis*:TNZT - CaP	-1,177	-3,348 to 0,9947	No	ns	0,9826
S. gordonii:TAV - Control vs. *P. gingivalis*:TNZT - CaPAg	-1,927	-4,098 to 0,2447	No	ns	0,1833
S. gordonii:TAV - Control vs. *P. gingivalis*:TNZT - HF	-1,252	-3,423 to 0,9197	No	ns	0,9591
S. gordonii:TAV - Control vs. *P. gingivalis*:TNZT - HFAg	-1,454	-3,732 to 0,8230	No	ns	0,8684
S. gordonii:TAV - CaP vs. *S. gordonii*:TAV - CaPAg	-0,5117	-2,683 to 1,660	No	ns	>0,9999
S. gordonii:TAV - CaP vs. *S. gordonii*:TAV - HF	-0,5383	-2,710 to 1,633	No	ns	>0,9999

S. gordonii:TAV - CaP vs. *S. gordonii*:TAV - HFAg	-0,3117	-2,483 to 1,860	No	ns	>0,9999
S. gordonii:TAV - CaP vs. *S. gordonii*:TNZT - Control	-0,7383	-2,910 to 1,433	No	ns	>0,9999
S. gordonii:TAV - CaP vs. *S. gordonii*:TNZT - CaP	0,3993	-1,878 to 2,677	No	ns	>0,9999
S. gordonii:TAV - CaP vs. *S. gordonii*:TNZT - CaPAg	-0,7017	-2,873 to 1,470	No	ns	>0,9999
S. gordonii:TAV - CaP vs. *S. gordonii*:TNZT - HF	-0,2600	-2,431 to 1,911	No	ns	>0,9999
S. gordonii:TAV - CaP vs. *S. gordonii*:TNZT - HFAg	-0,3983	-2,570 to 1,773	No	ns	>0,9999
S. gordonii:TAV - CaP vs. *S. sanguinis*:TAV - Control	-1,358	-3,530 to 0,8131	No	ns	0,8934
S. gordonii:TAV - CaP vs. *S. sanguinis*:TAV - CaP	-1,608	-3,780 to 0,5631	No	ns	0,5834
S. gordonii:TAV - CaP vs. *S. sanguinis*:TAV - CaPAg	-1,435	-3,606 to 0,7364	No	ns	0,8185
S. gordonii:TAV - CaP vs. *S. sanguinis*:TAV - HF	-1,655	-3,826 to 0,5164	No	ns	0,5139
S. gordonii:TAV - CaP vs. *S. sanguinis*:TAV - HFAg	-1,393	-3,565 to 0,7781	No	ns	0,8621
S. gordonii:TAV - CaP vs. *S. sanguinis*:TNZT - Control	-1,408	-3,580 to 0,7631	No	ns	0,8471
***S. gordonii*:TAV - CaP vs. *S. sanguinis*:TNZT - CaP**	**-2,273**	**-4,445 to -0,1019**	**Yes**	*****	**0,0267**
S. gordonii:TAV - CaP vs. *S. sanguinis*:TNZT - CaPAg	-1,865	-4,036 to 0,3064	No	ns	0,2414
S. gordonii:TAV - CaP vs. *S. sanguinis*:TNZT - HF	-1,635	-3,912 to 0,6427	No	ns	0,6568
S. gordonii:TAV - CaP vs. *S. sanguinis*:TNZT - HFAg	-1,767	-4,044 to 0,5107	No	ns	0,4706
S. gordonii:TAV - CaP vs. *A. naeslundii*:TAV - Control	-0,3567	-2,528 to 1,815	No	ns	>0,9999
S. gordonii:TAV - CaP vs. *A. naeslundii*:TAV - CaP	0,5433	-1,884 to 2,971	No	ns	>0,9999
S. gordonii:TAV - CaP vs. *A. naeslundii*:TAV - CaPAg	-0,4133	-2,585 to 1,758	No	ns	>0,9999
S. gordonii:TAV - CaP vs. *A. naeslundii*:TAV - HF	-0,3983	-2,570 to 1,773	No	ns	>0,9999
S. gordonii:TAV - CaP vs. *A. naeslundii*:TAV - HFAg	-0,2017	-2,373 to 1,970	No	ns	>0,9999
S. gordonii:TAV - CaP vs. *A. naeslundii*:TNZT - Control	-0,4400	-2,611 to 1,731	No	ns	>0,9999
S. gordonii:TAV - CaP vs. *A. naeslundii*:TNZT - CaP	-0,3567	-2,528 to 1,815	No	ns	>0,9999
S. gordonii:TAV - CaP vs. *A. naeslundii*:TNZT - CaPAg	-0,5350	-2,706 to 1,636	No	ns	>0,9999
S. gordonii:TAV - CaP vs. *A. naeslundii*:TNZT - HF	0,01333	-2,158 to 2,185	No	ns	>0,9999
S. gordonii:TAV - CaP vs. *A. naeslundii*:TNZT - HFAg	-0,7042	-3,132 to 1,724	No	ns	>0,9999
S. gordonii:TAV - CaP vs. *P. gingivalis*:TAV - Control	-1,190	-3,361 to 0,9814	No	ns	0,9795
***S. gordonii*:TAV - CaP vs. *P. gingivalis*:TAV - CaP**	**-2,345**	**-4,516 to -0,1736**	**Yes**	*****	**0,0168**
***S. gordonii*:TAV - CaP vs. *P. gingivalis*:TAV - CaPAg**	**-2,453**	**-4,625 to -0,2819**	**Yes**	******	**0,0080**
S. gordonii:TAV - CaP vs. *P. gingivalis*:TAV - HF	-1,625	-3,796 to 0,5464	No	ns	0,5585
S. gordonii:TAV - CaP vs. *P. gingivalis*:TAV - HFAg	-2,135	-4,412 to 0,1427	No	ns	0,1070
***S. gordonii*:TAV - CaP vs. *P. gingivalis*:TNZT - Control**	**-2,350**	**-4,521 to -0,1786**	**Yes**	*****	**0,0162**
S. gordonii:TAV - CaP vs. *P. gingivalis*:TNZT - CaP	-1,835	-4,006 to 0,3364	No	ns	0,2737
***S. gordonii*:TAV - CaP vs. *P. gingivalis*:TNZT - CaPAg**	**-2,585**	**-4,756 to -0,4136**	**Yes**	******	**0,0030**
S. gordonii:TAV - CaP vs. *P. gingivalis*:TNZT - HF	-1,910	-4,081 to 0,2614	No	ns	0,1979
S. gordonii:TAV - CaP vs. *P. gingivalis*:TNZT - HFAg	-2,113	-4,390 to 0,1647	No	ns	0,1193
S. gordonii:TAV - CaPAg vs. *S. gordonii*:TAV - HF	-0,02667	-2,198 to 2,145	No	ns	>0,9999
S. gordonii:TAV - CaPAg vs. *S. gordonii*:TAV - HFAg	0,2000	-1,971 to 2,371	No	ns	>0,9999
S. gordonii:TAV - CaPAg vs. *S. gordonii*:TNZT - Control	-0,2267	-2,398 to 1,945	No	ns	>0,9999
S. gordonii:TAV - CaPAg vs. *S. gordonii*:TNZT - CaP	0,9110	-1,366 to 3,188	No	ns	>0,9999
S. gordonii:TAV - CaPAg vs. *S. gordonii*:TNZT - CaPAg	-0,1900	-2,361 to 1,981	No	ns	>0,9999
S. gordonii:TAV - CaPAg vs. *S. gordonii*:TNZT - HF	0,2517	-1,920 to 2,423	No	ns	>0,9999
S. gordonii:TAV - CaPAg vs. *S. gordonii*:TNZT - HFAg	0,1133	-2,058 to 2,285	No	ns	>0,9999
S. gordonii:TAV - CaPAg vs. *S. sanguinis*:TAV - Control	-0,8467	-3,018 to 1,325	No	ns	>0,9999
S. gordonii:TAV - CaPAg vs. *S. sanguinis*:TAV - CaP	-1,097	-3,268 to 1,075	No	ns	0,9943
S. gordonii:TAV - CaPAg vs. *S. sanguinis*:TAV - CaPAg	-0,9233	-3,095 to 1,248	No	ns	0,9998
S. gordonii:TAV - CaPAg vs. *S. sanguinis*:TAV - HF	-1,143	-3,315 to 1,028	No	ns	0,9888
S. gordonii:TAV - CaPAg vs. *S. sanguinis*:TAV - HFAg	-0,8817	-3,053 to 1,290	No	ns	>0,9999
S. gordonii:TAV - CaPAg vs. *S. sanguinis*:TNZT - Control	-0,8967	-3,068 to 1,275	No	ns	0,9999
S. gordonii:TAV - CaPAg vs. *S. sanguinis*:TNZT - CaP	-1,762	-3,933 to 0,4097	No	ns	0,3632
S. gordonii:TAV - CaPAg vs. *S. sanguinis*:TNZT - CaPAg	-1,353	-3,525 to 0,8181	No	ns	0,8974
S. gordonii:TAV - CaPAg vs. *S. sanguinis*:TNZT - HF	-1,123	-3,400 to 1,154	No	ns	0,9962
S. gordonii:TAV - CaPAg vs. *S. sanguinis*:TNZT - HFAg	-1,255	-3,532 to 1,022	No	ns	0,9778
S. gordonii:TAV - CaPAg vs. *A. naeslundii*:TAV - Control	0,1550	-2,016 to 2,326	No	ns	>0,9999
S. gordonii:TAV - CaPAg vs. *A. naeslundii*:TAV - CaP	1,055	-1,373 to 3,483	No	ns	0,9997
S. gordonii:TAV - CaPAg vs. *A. naeslundii*:TAV - CaPAg	0,09833	-2,073 to 2,270	No	ns	>0,9999
S. gordonii:TAV - CaPAg vs. *A. naeslundii*:TAV - HF	0,1133	-2,058 to 2,285	No	ns	>0,9999
S. gordonii:TAV - CaPAg vs. *A. naeslundii*:TAV - HFAg	0,3100	-1,861 to 2,481	No	ns	>0,9999
S. gordonii:TAV - CaPAg vs. *A. naeslundii*:TNZT - Control	0,07167	-2,100 to 2,243	No	ns	>0,9999
S. gordonii:TAV - CaPAg vs. *A. naeslundii*:TNZT - CaP	0,1550	-2,016 to 2,326	No	ns	>0,9999
S. gordonii:TAV - CaPAg vs. *A. naeslundii*:TNZT - CaPAg	-0,02333	-2,195 to 2,148	No	ns	>0,9999
S. gordonii:TAV - CaPAg vs. *A. naeslundii*:TNZT - HF	0,5250	-1,646 to 2,696	No	ns	>0,9999
S. gordonii:TAV - CaPAg vs. *A. naeslundii*:TNZT - HFAg	-0,1925	-2,620 to 2,235	No	ns	>0,9999
S. gordonii:TAV - CaPAg vs. *P. gingivalis*:TAV - Control	-0,6783	-2,850 to 1,493	No	ns	>0,9999
S. gordonii:TAV - CaPAg vs. *P. gingivalis*:TAV - CaP	-1,833	-4,005 to 0,3381	No	ns	0,2756
S. gordonii:TAV - CaPAg vs. *P. gingivalis*:TAV - CaPAg	-1,942	-4,113 to 0,2297	No	ns	0,1709
S. gordonii:TAV - CaPAg vs. *P. gingivalis*:TAV - HF	-1,113	-3,285 to 1,058	No	ns	0,9927
S. gordonii:TAV - CaPAg vs. *P. gingivalis*:TAV - HFAg	-1,623	-3,900 to 0,6544	No	ns	0,6728
S. gordonii:TAV - CaPAg vs. *P. gingivalis*:TNZT - Control	-1,838	-4,010 to 0,3331	No	ns	0,2700
S. gordonii:TAV - CaPAg vs. *P. gingivalis*:TNZT - CaP	-1,323	-3,495 to 0,8481	No	ns	0,9197
S. gordonii:TAV - CaPAg vs. *P. gingivalis*:TNZT - CaPAg	-2,073	-4,245 to 0,09806	No	ns	0,0873
S. gordonii:TAV - CaPAg vs. *P. gingivalis*:TNZT - HF	-1,398	-3,570 to 0,7731	No	ns	0,8572

S. gordonii:TAV - CaPAg vs. *P. gingivalis*:TNZT - HFAg	-1,601	-3,878 to 0,6764	No	ns	0,7024
S. gordonii:TAV - HF vs. *S. gordonii*:TAV - HFAg	0,2267	-1,945 to 2,398	No	ns	>0,9999
S. gordonii:TAV - HF vs. *S. gordonii*:TNZT - Control	-0,2000	-2,371 to 1,971	No	ns	>0,9999
S. gordonii:TAV - HF vs. *S. gordonii*:TNZT - CaP	0,9377	-1,340 to 3,215	No	ns	0,9999
S. gordonii:TAV - HF vs. *S. gordonii*:TNZT - CaPAg	-0,1633	-2,335 to 2,008	No	ns	>0,9999
S. gordonii:TAV - HF vs. *S. gordonii*:TNZT - HF	0,2783	-1,893 to 2,450	No	ns	>0,9999
S. gordonii:TAV - HF vs. *S. gordonii*:TNZT - HFAg	0,1400	-2,031 to 2,311	No	ns	>0,9999
S. gordonii:TAV - HF vs. *S. sanguinis*:TAV - Control	-0,8200	-2,991 to 1,351	No	ns	>0,9999
S. gordonii:TAV - HF vs. *S. sanguinis*:TAV - CaP	-1,070	-3,241 to 1,101	No	ns	0,9963
S. gordonii:TAV - HF vs. *S. sanguinis*:TAV - CaPAg	-0,8967	-3,068 to 1,275	No	ns	0,9999
S. gordonii:TAV - HF vs. *S. sanguinis*:TAV - HF	-1,117	-3,288 to 1,055	No	ns	0,9923
S. gordonii:TAV - HF vs. *S. sanguinis*:TAV - HFAg	-0,8550	-3,026 to 1,316	No	ns	>0,9999
S. gordonii:TAV - HF vs. *S. sanguinis*:TNZT - Control	-0,8700	-3,041 to 1,301	No	ns	>0,9999
S. gordonii:TAV - HF vs. *S. sanguinis*:TNZT - CaP	-1,735	-3,906 to 0,4364	No	ns	0,3990
S. gordonii:TAV - HF vs. *S. sanguinis*:TNZT - CaPAg	-1,327	-3,498 to 0,8447	No	ns	0,9174
S. gordonii:TAV - HF vs. *S. sanguinis*:TNZT - HF	-1,096	-3,374 to 1,181	No	ns	0,9975
S. gordonii:TAV - HF vs. *S. sanguinis*:TNZT - HFAg	-1,228	-3,506 to 1,049	No	ns	0,9838
S. gordonii:TAV - HF vs. *A. naeslundii*:TAV - Control	0,1817	-1,990 to 2,353	No	ns	>0,9999
S. gordonii:TAV - HF vs. *A. naeslundii*:TAV - CaP	1,082	-1,346 to 3,509	No	ns	0,9995
S. gordonii:TAV - HF vs. *A. naeslundii*:TAV - CaPAg	0,1250	-2,046 to 2,296	No	ns	>0,9999
S. gordonii:TAV - HF vs. *A. naeslundii*:TAV - HF	0,1400	-2,031 to 2,311	No	ns	>0,9999
S. gordonii:TAV - HF vs. *A. naeslundii*:TAV - HFAg	0,3367	-1,835 to 2,508	No	ns	>0,9999
S. gordonii:TAV - HF vs. *A. naeslundii*:TNZT - Control	0,09833	-2,073 to 2,270	No	ns	>0,9999
S. gordonii:TAV - HF vs. *A. naeslundii*:TNZT - CaP	0,1817	-1,990 to 2,353	No	ns	>0,9999
S. gordonii:TAV - HF vs. *A. naeslundii*:TNZT - CaPAg	0,003333	-2,168 to 2,175	No	ns	>0,9999
S. gordonii:TAV - HF vs. *A. naeslundii*:TNZT - HF	0,5517	-1,620 to 2,723	No	ns	>0,9999
S. gordonii:TAV - HF vs. *A. naeslundii*:TNZT - HFAg	-0,1658	-2,594 to 2,262	No	ns	>0,9999
S. gordonii:TAV - HF vs. *P. gingivalis*:TAV - Control	-0,6517	-2,823 to 1,520	No	ns	>0,9999
S. gordonii:TAV - HF vs. *P. gingivalis*:TAV - CaP	-1,807	-3,978 to 0,3647	No	ns	0,3066
S. gordonii:TAV - HF vs. *P. gingivalis*:TAV - CaPAg	-1,915	-4,086 to 0,2564	No	ns	0,1934
S. gordonii:TAV - HF vs. *P. gingivalis*:TAV - HF	-1,087	-3,258 to 1,085	No	ns	0,9951
S. gordonii:TAV - HF vs. *P. gingivalis*:TAV - HFAg	-1,596	-3,874 to 0,6810	No	ns	0,7086
S. gordonii:TAV - HF vs. *P. gingivalis*:TNZT - Control	-1,812	-3,983 to 0,3597	No	ns	0,3006
S. gordonii:TAV - HF vs. *P. gingivalis*:TNZT - CaP	-1,297	-3,468 to 0,8747	No	ns	0,9366
S. gordonii:TAV - HF vs. *P. gingivalis*:TNZT - CaPAg	-2,047	-4,218 to 0,1247	No	ns	0,1008
S. gordonii:TAV - HF vs. *P. gingivalis*:TNZT - HF	-1,372	-3,543 to 0,7997	No	ns	0,8820
S. gordonii:TAV - HF vs. *P. gingivalis*:TNZT - HFAg	-1,574	-3,852 to 0,7030	No	ns	0,7371
S. gordonii:TAV - HFAg vs. *S. gordonii*:TNZT - Control	-0,4267	-2,598 to 1,745	No	ns	>0,9999
S. gordonii:TAV - HFAg vs. *S. gordonii*:TNZT - CaP	0,7110	-1,566 to 2,988	No	ns	>0,9999
S. gordonii:TAV - HFAg vs. *S. gordonii*:TNZT - CaPAg	-0,3900	-2,561 to 1,781	No	ns	>0,9999
S. gordonii:TAV - HFAg vs. *S. gordonii*:TNZT - HF	0,05167	-2,120 to 2,223	No	ns	>0,9999
S. gordonii:TAV - HFAg vs. *S. gordonii*:TNZT - HFAg	-0,08667	-2,258 to 2,085	No	ns	>0,9999
S. gordonii:TAV - HFAg vs. *S. sanguinis*:TAV - Control	-1,047	-3,218 to 1,125	No	ns	0,9975
S. gordonii:TAV - HFAg vs. *S. sanguinis*:TAV - CaP	-1,297	-3,468 to 0,8747	No	ns	0,9366
S. gordonii:TAV - HFAg vs. *S. sanguinis*:TAV - CaPAg	-1,123	-3,295 to 1,048	No	ns	0,9915
S. gordonii:TAV - HFAg vs. *S. sanguinis*:TAV - HF	-1,343	-3,515 to 0,8281	No	ns	0,9053
S. gordonii:TAV - HFAg vs. *S. sanguinis*:TAV - HFAg	-1,082	-3,253 to 1,090	No	ns	0,9955
S. gordonii:TAV - HFAg vs. *S. sanguinis*:TNZT - Control	-1,097	-3,268 to 1,075	No	ns	0,9943
S. gordonii:TAV - HFAg vs. *S. sanguinis*:TNZT - CaP	-1,962	-4,133 to 0,2097	No	ns	0,1552
S. gordonii:TAV - HFAg vs. *S. sanguinis*:TNZT - CaPAg	-1,553	-3,725 to 0,6181	No	ns	0,6644
S. gordonii:TAV - HFAg vs. *S. sanguinis*:TNZT - HF	-1,323	-3,600 to 0,9544	No	ns	0,9548
S. gordonii:TAV - HFAg vs. *S. sanguinis*:TNZT - HFAg	-1,455	-3,732 to 0,8224	No	ns	0,8678
S. gordonii:TAV - HFAg vs. *A. naeslundii*:TAV - Control	-0,04500	-2,216 to 2,126	No	ns	>0,9999
S. gordonii:TAV - HFAg vs. *A. naeslundii*:TAV - CaP	0,8550	-1,573 to 3,283	No	ns	>0,9999
S. gordonii:TAV - HFAg vs. *A. naeslundii*:TAV - CaPAg	-0,1017	-2,273 to 2,070	No	ns	>0,9999
S. gordonii:TAV - HFAg vs. *A. naeslundii*:TAV - HF	-0,08667	-2,258 to 2,085	No	ns	>0,9999
S. gordonii:TAV - HFAg vs. *A. naeslundii*:TAV - HFAg	0,1100	-2,061 to 2,281	No	ns	>0,9999
S. gordonii:TAV - HFAg vs. *A. naeslundii*:TNZT - Control	-0,1283	-2,300 to 2,043	No	ns	>0,9999
S. gordonii:TAV - HFAg vs. *A. naeslundii*:TNZT - CaP	-0,04500	-2,216 to 2,126	No	ns	>0,9999
S. gordonii:TAV - HFAg vs. *A. naeslundii*:TNZT - CaPAg	-0,2233	-2,395 to 1,948	No	ns	>0,9999
S. gordonii:TAV - HFAg vs. *A. naeslundii*:TNZT - HF	0,3250	-1,846 to 2,496	No	ns	>0,9999
S. gordonii:TAV - HFAg vs. *A. naeslundii*:TNZT - HFAg	-0,3925	-2,820 to 2,035	No	ns	>0,9999
S. gordonii:TAV - HFAg vs. *P. gingivalis*:TAV - Control	-0,8783	-3,050 to 1,293	No	ns	>0,9999
S. gordonii:TAV - HFAg vs. *P. gingivalis*:TAV - CaP	-2,033	-4,205 to 0,1381	No	ns	0,1081
S. gordonii:TAV - HFAg vs. *P. gingivalis*:TAV - CaPAg	-2,142	-4,313 to 0,02972	No	ns	0,0595
S. gordonii:TAV - HFAg vs. *P. gingivalis*:TAV - HF	-1,313	-3,485 to 0,8581	No	ns	0,9264
S. gordonii:TAV - HFAg vs. *P. gingivalis*:TAV - HFAg	-1,823	-4,100 to 0,4544	No	ns	0,3947
S. gordonii:TAV - HFAg vs. *P. gingivalis*:TNZT - Control	-2,038	-4,210 to 0,1331	No	ns	0,1053
S. gordonii:TAV - HFAg vs. *P. gingivalis*:TNZT - CaP	-1,523	-3,695 to 0,6481	No	ns	0,7068
***S. gordonii*:TAV - HFAg vs. *P. gingivalis*:TNZT - CaPAg**	**-2,273**	**-4,445 to -0,1019**	**Yes**	*****	**0,0267**
S. gordonii:TAV - HFAg vs. *P. gingivalis*:TNZT - HF	-1,598	-3,770 to 0,5731	No	ns	0,5983
S. gordonii:TAV - HFAg vs. *P. gingivalis*:TNZT - HFAg	-1,801	-4,078 to 0,4764	No	ns	0,4237

S. gordonii:TNZT - Control vs. *S. gordonii*:TNZT - CaP	1,138	-1,140 to 3,415	No	ns	0,9953
S. gordonii:TNZT - Control vs. *S. gordonii*:TNZT - CaPAg	0,03667	-2,135 to 2,208	No	ns	>0,9999
S. gordonii:TNZT - Control vs. *S. gordonii*:TNZT - HF	0,4783	-1,693 to 2,650	No	ns	>0,9999
S. gordonii:TNZT - Control vs. *S. gordonii*:TNZT - HFAg	0,3400	-1,831 to 2,511	No	ns	>0,9999
S. gordonii:TNZT - Control vs. *S. sanguinis*:TAV - Control	-0,6200	-2,791 to 1,551	No	ns	>0,9999
S. gordonii:TNZT - Control vs. *S. sanguinis*:TAV - CaP	-0,8700	-3,041 to 1,301	No	ns	>0,9999
S. gordonii:TNZT - Control vs. *S. sanguinis*:TAV - CaPAg	-0,6967	-2,868 to 1,475	No	ns	>0,9999
S. gordonii:TNZT - Control vs. *S. sanguinis*:TAV - HF	-0,9167	-3,088 to 1,255	No	ns	0,9998
S. gordonii:TNZT - Control vs. *S. sanguinis*:TAV - HFAg	-0,6550	-2,826 to 1,516	No	ns	>0,9999
S. gordonii:TNZT - Control vs. *S. sanguinis*:TNZT - Control	-0,6700	-2,841 to 1,501	No	ns	>0,9999
S. gordonii:TNZT - Control vs. *S. sanguinis*:TNZT - CaP	-1,535	-3,706 to 0,6364	No	ns	0,6905
S. gordonii:TNZT - Control vs. *S. sanguinis*:TNZT - CaPAg	-1,127	-3,298 to 1,045	No	ns	0,9911
S. gordonii:TNZT - Control vs. *S. sanguinis*:TNZT - HF	-0,8963	-3,174 to 1,381	No	ns	>0,9999
S. gordonii:TNZT - Control vs. *S. sanguinis*:TNZT - HFAg	-1,028	-3,306 to 1,249	No	ns	0,9993
S. gordonii:TNZT - Control vs. *A. naeslundii*:TAV - Control	0,3817	-1,790 to 2,553	No	ns	>0,9999
S. gordonii:TNZT - Control vs. *A. naeslundii*:TAV - CaP	1,282	-1,146 to 3,709	No	ns	0,9883
S. gordonii:TNZT - Control vs. *A. naeslundii*:TAV - CaPAg	0,3250	-1,846 to 2,496	No	ns	>0,9999
S. gordonii:TNZT - Control vs. *A. naeslundii*:TAV - HF	0,3400	-1,831 to 2,511	No	ns	>0,9999
S. gordonii:TNZT - Control vs. *A. naeslundii*:TAV - HFAg	0,5367	-1,635 to 2,708	No	ns	>0,9999
S. gordonii:TNZT - Control vs. *A. naeslundii*:TNZT - Control	0,2983	-1,873 to 2,470	No	ns	>0,9999
S. gordonii:TNZT - Control vs. *A. naeslundii*:TNZT - CaP	0,3817	-1,790 to 2,553	No	ns	>0,9999
S. gordonii:TNZT - Control vs. *A. naeslundii*:TNZT - CaPAg	0,2033	-1,968 to 2,375	No	ns	>0,9999
S. gordonii:TNZT - Control vs. *A. naeslundii*:TNZT - HF	0,7517	-1,420 to 2,923	No	ns	>0,9999
S. gordonii:TNZT - Control vs. *A. naeslundii*:TNZT - HFAg	0,03417	-2,394 to 2,462	No	ns	>0,9999
S. gordonii:TNZT - Control vs. *P. gingivalis*:TAV - Control	-0,4517	-2,623 to 1,720	No	ns	>0,9999
S. gordonii:TNZT - Control vs. *P. gingivalis*:TAV - CaP	-1,607	-3,778 to 0,5647	No	ns	0,5859
S. gordonii:TNZT - Control vs. *P. gingivalis*:TAV - CaPAg	-1,715	-3,886 to 0,4564	No	ns	0,4268
S. gordonii:TNZT - Control vs. *P. gingivalis*:TAV - HF	-0,8867	-3,058 to 1,285	No	ns	>0,9999
S. gordonii:TNZT - Control vs. *P. gingivalis*:TAV - HFAg	-1,396	-3,674 to 0,8810	No	ns	0,9141
S. gordonii:TNZT - Control vs. *P. gingivalis*:TNZT - Control	-1,612	-3,783 to 0,5597	No	ns	0,5784
S. gordonii:TNZT - Control vs. *P. gingivalis*:TNZT - CaP	-1,097	-3,268 to 1,075	No	ns	0,9943
S. gordonii:TNZT - Control vs. *P. gingivalis*:TNZT - CaPAg	-1,847	-4,018 to 0,3247	No	ns	0,2608
S. gordonii:TNZT - Control vs. *P. gingivalis*:TNZT - HF	-1,172	-3,343 to 0,9997	No	ns	0,9837
S. gordonii:TNZT - Control vs. *P. gingivalis*:TNZT - HFAg	-1,374	-3,652 to 0,9030	No	ns	0,9283
S. gordonii:TNZT - CaP vs. *S. gordonii*:TNZT - CaPAg	-1,101	-3,378 to 1,176	No	ns	0,9974
S. gordonii:TNZT - CaP vs. *S. gordonii*:TNZT - HF	-0,6593	-2,937 to 1,618	No	ns	>0,9999
S. gordonii:TNZT - CaP vs. *S. gordonii*:TNZT - HFAg	-0,7977	-3,075 to 1,480	No	ns	>0,9999
S. gordonii:TNZT - CaP vs. *S. sanguinis*:TAV - Control	-1,758	-4,035 to 0,5197	No	ns	0,4831
S. gordonii:TNZT - CaP vs. *S. sanguinis*:TAV - CaP	-2,008	-4,285 to 0,2697	No	ns	0,1941
S. gordonii:TNZT - CaP vs. *S. sanguinis*:TAV - CaPAg	-1,834	-4,112 to 0,4430	No	ns	0,3800
S. gordonii:TNZT - CaP vs. *S. sanguinis*:TAV - HF	-2,054	-4,332 to 0,2230	No	ns	0,1575
S. gordonii:TNZT - CaP vs. *S. sanguinis*:TAV - HFAg	-1,793	-4,070 to 0,4847	No	ns	0,4349
S. gordonii:TNZT - CaP vs. *S. sanguinis*:TNZT - Control	-1,808	-4,085 to 0,4697	No	ns	0,4148
***S. gordonii*:TNZT - CaP vs. *S. sanguinis*:TNZT - CaP**	**-2,673**	**-4,950 to -0,3953**	**Yes**	******	**0,0040**
S. gordonii:TNZT - CaP vs. *S. sanguinis*:TNZT - CaPAg	-2,264	-4,542 to 0,01304	No	ns	0,0538
S. gordonii:TNZT - CaP vs. *S. sanguinis*:TNZT - HF	-2,034	-4,413 to 0,3446	No	ns	0,2500
S. gordonii:TNZT - CaP vs. *S. sanguinis*:TNZT - HFAg	-2,166	-4,545 to 0,2126	No	ns	0,1438
S. gordonii:TNZT - CaP vs. *A. naeslundii*:TAV - Control	-0,7560	-3,033 to 1,521	No	ns	>0,9999
S. gordonii:TNZT - CaP vs. *A. naeslundii*:TAV - CaP	0,1440	-2,379 to 2,667	No	ns	>0,9999
S. gordonii:TNZT - CaP vs. *A. naeslundii*:TAV - CaPAg	-0,8127	-3,090 to 1,465	No	ns	>0,9999
S. gordonii:TNZT - CaP vs. *A. naeslundii*:TAV - HF	-0,7977	-3,075 to 1,480	No	ns	>0,9999
S. gordonii:TNZT - CaP vs. *A. naeslundii*:TAV - HFAg	-0,6010	-2,878 to 1,676	No	ns	>0,9999
S. gordonii:TNZT - CaP vs. *A. naeslundii*:TNZT - Control	-0,8393	-3,117 to 1,438	No	ns	>0,9999
S. gordonii:TNZT - CaP vs. *A. naeslundii*:TNZT - CaP	-0,7560	-3,033 to 1,521	No	ns	>0,9999
S. gordonii:TNZT - CaP vs. *A. naeslundii*:TNZT - CaPAg	-0,9343	-3,212 to 1,343	No	ns	>0,9999
S. gordonii:TNZT - CaP vs. *A. naeslundii*:TNZT - HF	-0,3860	-2,663 to 1,891	No	ns	>0,9999
S. gordonii:TNZT - CaP vs. *A. naeslundii*:TNZT - HFAg	-1,104	-3,626 to 1,419	No	ns	0,9996
S. gordonii:TNZT - CaP vs. *P. gingivalis*:TAV - Control	-1,589	-3,867 to 0,6880	No	ns	0,7177
***S. gordonii*:TNZT - CaP vs. *P. gingivalis*:TAV - CaP**	**-2,744**	**-5,022 to -0,4670**	**Yes**	******	**0,0024**
***S. gordonii*:TNZT - CaP vs. *P. gingivalis*:TAV - CaPAg**	**-2,853**	**-5,130 to -0,5753**	**Yes**	******	**0,0011**
S. gordonii:TNZT - CaP vs. *P. gingivalis*:TAV - HF	-2,024	-4,302 to 0,2530	No	ns	0,1804
***S. gordonii*:TNZT - CaP vs. *P. gingivalis*:TAV - HFAg**	**-2,534**	**-4,913 to -0,1554**	**Yes**	*****	**0,0207**
***S. gordonii*:TNZT - CaP vs. *P. gingivalis*:TNZT - Control**	**-2,749**	**-5,027 to -0,4720**	**Yes**	******	**0,0023**
S. gordonii:TNZT - CaP vs. *P. gingivalis*:TNZT - CaP	-2,234	-4,512 to 0,04304	No	ns	0,0635
***S. gordonii*:TNZT - CaP vs. *P. gingivalis*:TNZT - CaPAg**	**-2,984**	**-5,262 to -0,7070**	**Yes**	*******	**0,0004**
***S. gordonii*:TNZT - CaP vs. *P. gingivalis*:TNZT - HF**	**-2,309**	**-4,587 to -0,03196**	**Yes**	*****	**0,0417**
***S. gordonii*:TNZT - CaP vs. *P. gingivalis*:TNZT - HFAg**	**-2,512**	**-4,891 to -0,1334**	**Yes**	*****	**0,0235**
S. gordonii:TNZT - CaPAg vs. *S. gordonii*:TNZT - HF	0,4417	-1,730 to 2,613	No	ns	>0,9999
S. gordonii:TNZT - CaPAg vs. *S. gordonii*:TNZT - HFAg	0,3033	-1,868 to 2,475	No	ns	>0,9999
S. gordonii:TNZT - CaPAg vs. *S. sanguinis*:TAV - Control	-0,6567	-2,828 to 1,515	No	ns	>0,9999
S. gordonii:TNZT - CaPAg vs. *S. sanguinis*:TAV - CaP	-0,9067	-3,078 to 1,265	No	ns	0,9999
S. gordonii:TNZT - CaPAg vs. *S. sanguinis*:TAV - CaPAg	-0,7333	-2,905 to 1,438	No	ns	>0,9999

S. gordonii:TNZT - CaPAg vs. *S. sanguinis*:TAV - HF	-0,9533	-3,125 to 1,218	No	ns	0,9996
S. gordonii:TNZT - CaPAg vs. *S. sanguinis*:TAV - HFAg	-0,6917	-2,863 to 1,480	No	ns	>0,9999
S. gordonii:TNZT - CaPAg vs. *S. sanguinis*:TNZT - Control	-0,7067	-2,878 to 1,465	No	ns	>0,9999
S. gordonii:TNZT - CaPAg vs. *S. sanguinis*:TNZT - CaP	-1,572	-3,743 to 0,5997	No	ns	0,6377
S. gordonii:TNZT - CaPAg vs. *S. sanguinis*:TNZT - CaPAg	-1,163	-3,335 to 1,008	No	ns	0,9854
S. gordonii:TNZT - CaPAg vs. *S. sanguinis*:TNZT - HF	-0,9330	-3,210 to 1,344	No	ns	>0,9999
S. gordonii:TNZT - CaPAg vs. *S. sanguinis*:TNZT - HFAg	-1,065	-3,342 to 1,212	No	ns	0,9986
S. gordonii:TNZT - CaPAg vs. *A. naeslundii*:TAV - Control	0,3450	-1,826 to 2,516	No	ns	>0,9999
S. gordonii:TNZT - CaPAg vs. *A. naeslundii*:TAV - CaP	1,245	-1,183 to 3,673	No	ns	0,9927
S. gordonii:TNZT - CaPAg vs. *A. naeslundii*:TAV - CaPAg	0,2883	-1,883 to 2,460	No	ns	>0,9999
S. gordonii:TNZT - CaPAg vs. *A. naeslundii*:TAV - HF	0,3033	-1,868 to 2,475	No	ns	>0,9999
S. gordonii:TNZT - CaPAg vs. *A. naeslundii*:TAV - HFAg	0,5000	-1,671 to 2,671	No	ns	>0,9999
S. gordonii:TNZT - CaPAg vs. *A. naeslundii*:TNZT - Control	0,2617	-1,910 to 2,433	No	ns	>0,9999
S. gordonii:TNZT - CaPAg vs. *A. naeslundii*:TNZT - CaP	0,3450	-1,826 to 2,516	No	ns	>0,9999
S. gordonii:TNZT - CaPAg vs. *A. naeslundii*:TNZT - CaPAg	0,1667	-2,005 to 2,338	No	ns	>0,9999
S. gordonii:TNZT - CaPAg vs. *A. naeslundii*:TNZT - HF	0,7150	-1,456 to 2,886	No	ns	>0,9999
S. gordonii:TNZT - CaPAg vs. *A. naeslundii*:TNZT - HFAg	-0,002500	-2,430 to 2,425	No	ns	>0,9999
S. gordonii:TNZT - CaPAg vs. *P. gingivalis*:TAV - Control	-0,4883	-2,660 to 1,683	No	ns	>0,9999
S. gordonii:TNZT - CaPAg vs. *P. gingivalis*:TAV - CaP	-1,643	-3,815 to 0,5281	No	ns	0,5312
S. gordonii:TNZT - CaPAg vs. *P. gingivalis*:TAV - CaPAg	-1,752	-3,923 to 0,4197	No	ns	0,3764
S. gordonii:TNZT - CaPAg vs. *P. gingivalis*:TAV - HF	-0,9233	-3,095 to 1,248	No	ns	0,9998
S. gordonii:TNZT - CaPAg vs. *P. gingivalis*:TAV - HFAg	-1,433	-3,710 to 0,8444	No	ns	0,8867
S. gordonii:TNZT - CaPAg vs. *P. gingivalis*:TNZT - Control	-1,648	-3,820 to 0,5231	No	ns	0,5238
S. gordonii:TNZT - CaPAg vs. *P. gingivalis*:TNZT - CaP	-1,133	-3,305 to 1,038	No	ns	0,9902
S. gordonii:TNZT - CaPAg vs. *P. gingivalis*:TNZT - CaPAg	-1,883	-4,055 to 0,2881	No	ns	0,2230
S. gordonii:TNZT - CaPAg vs. *P. gingivalis*:TNZT - HF	-1,208	-3,380 to 0,9631	No	ns	0,9745
S. gordonii:TNZT - CaPAg vs. *P. gingivalis*:TNZT - HFAg	-1,411	-3,688 to 0,8664	No	ns	0,9037
S. gordonii:TNZT - HF vs. *S. gordonii*:TNZT - HFAg	-0,1383	-2,310 to 2,033	No	ns	>0,9999
S. gordonii:TNZT - HF vs. *S. sanguinis*:TAV - Control	-1,098	-3,270 to 1,073	No	ns	0,9942
S. gordonii:TNZT - HF vs. *S. sanguinis*:TAV - CaP	-1,348	-3,520 to 0,8231	No	ns	0,9014
S. gordonii:TNZT - HF vs. *S. sanguinis*:TAV - CaPAg	-1,175	-3,346 to 0,9964	No	ns	0,9830
S. gordonii:TNZT - HF vs. *S. sanguinis*:TAV - HF	-1,395	-3,566 to 0,7764	No	ns	0,8604
S. gordonii:TNZT - HF vs. *S. sanguinis*:TAV - HFAg	-1,133	-3,305 to 1,038	No	ns	0,9902
S. gordonii:TNZT - HF vs. *S. sanguinis*:TNZT - Control	-1,148	-3,320 to 1,023	No	ns	0,9880
S. gordonii:TNZT - HF vs. *S. sanguinis*:TNZT - CaP	-2,013	-4,185 to 0,1581	No	ns	0,1199
S. gordonii:TNZT - HF vs. *S. sanguinis*:TNZT - CaPAg	-1,605	-3,776 to 0,5664	No	ns	0,5884
S. gordonii:TNZT - HF vs. *S. sanguinis*:TNZT - HF	-1,375	-3,652 to 0,9027	No	ns	0,9281
S. gordonii:TNZT - HF vs. *S. sanguinis*:TNZT - HFAg	-1,507	-3,784 to 0,7707	No	ns	0,8168
S. gordonii:TNZT - HF vs. *A. naeslundii*:TAV - Control	-0,09667	-2,268 to 2,075	No	ns	>0,9999
S. gordonii:TNZT - HF vs. *A. naeslundii*:TAV - CaP	0,8033	-1,624 to 3,231	No	ns	>0,9999
S. gordonii:TNZT - HF vs. *A. naeslundii*:TAV - CaPAg	-0,1533	-2,325 to 2,018	No	ns	>0,9999
S. gordonii:TNZT - HF vs. *A. naeslundii*:TAV - HF	-0,1383	-2,310 to 2,033	No	ns	>0,9999
S. gordonii:TNZT - HF vs. *A. naeslundii*:TAV - HFAg	0,05833	-2,113 to 2,230	No	ns	>0,9999
S. gordonii:TNZT - HF vs. *A. naeslundii*:TNZT - Control	-0,1800	-2,351 to 1,991	No	ns	>0,9999
S. gordonii:TNZT - HF vs. *A. naeslundii*:TNZT - CaP	-0,09667	-2,268 to 2,075	No	ns	>0,9999
S. gordonii:TNZT - HF vs. *A. naeslundii*:TNZT - CaPAg	-0,2750	-2,446 to 1,896	No	ns	>0,9999
S. gordonii:TNZT - HF vs. *A. naeslundii*:TNZT - HF	0,2733	-1,898 to 2,445	No	ns	>0,9999
S. gordonii:TNZT - HF vs. *A. naeslundii*:TNZT - HFAg	-0,4442	-2,872 to 1,984	No	ns	>0,9999
S. gordonii:TNZT - HF vs. *P. gingivalis*:TAV - Control	-0,9300	-3,101 to 1,241	No	ns	0,9998
S. gordonii:TNZT - HF vs. *P. gingivalis*:TAV - CaP	-2,085	-4,256 to 0,08639	No	ns	0,0819
***S. gordonii*:TNZT - HF vs. *P. gingivalis*:TAV - CaPAg**	**-2,193**	**-4,365 to -0,02194**	**Yes**	*****	**0,0439**
S. gordonii:TNZT - HF vs. *P. gingivalis*:TAV - HF	-1,365	-3,536 to 0,8064	No	ns	0,8878
S. gordonii:TNZT - HF vs. *P. gingivalis*:TAV - HFAg	-1,875	-4,152 to 0,4027	No	ns	0,3302
S. gordonii:TNZT - HF vs. *P. gingivalis*:TNZT - Control	-2,090	-4,261 to 0,08139	No	ns	0,0797
S. gordonii:TNZT - HF vs. *P. gingivalis*:TNZT - CaP	-1,575	-3,746 to 0,5964	No	ns	0,6328
***S. gordonii*:TNZT - HF vs. *P. gingivalis*:TNZT - CaPAg**	**-2,325**	**-4,496 to -0,1536**	**Yes**	*****	**0,0191**
S. gordonii:TNZT - HF vs. *P. gingivalis*:TNZT - HF	-1,650	-3,821 to 0,5214	No	ns	0,5213
S. gordonii:TNZT - HF vs. *P. gingivalis*:TNZT - HFAg	-1,853	-4,130 to 0,4247	No	ns	0,3569
S. gordonii:TNZT - HFAg vs. *S. sanguinis*:TAV - Control	-0,9600	-3,131 to 1,211	No	ns	0,9995
S. gordonii:TNZT - HFAg vs. *S. sanguinis*:TAV - CaP	-1,210	-3,381 to 0,9614	No	ns	0,9741
S. gordonii:TNZT - HFAg vs. *S. sanguinis*:TAV - CaPAg	-1,037	-3,208 to 1,135	No	ns	0,9979
S. gordonii:TNZT - HFAg vs. *S. sanguinis*:TAV - HF	-1,257	-3,428 to 0,9147	No	ns	0,9569
S. gordonii:TNZT - HFAg vs. *S. sanguinis*:TAV - HFAg	-0,9950	-3,166 to 1,176	No	ns	0,9990
S. gordonii:TNZT - HFAg vs. *S. sanguinis*:TNZT - Control	-1,010	-3,181 to 1,161	No	ns	0,9987
S. gordonii:TNZT - HFAg vs. *S. sanguinis*:TNZT - CaP	-1,875	-4,046 to 0,2964	No	ns	0,2312
S. gordonii:TNZT - HFAg vs. *S. sanguinis*:TNZT - CaPAg	-1,467	-3,638 to 0,7047	No	ns	0,7812
S. gordonii:TNZT - HFAg vs. *S. sanguinis*:TNZT - HF	-1,236	-3,514 to 1,041	No	ns	0,9822
S. gordonii:TNZT - HFAg vs. *S. sanguinis*:TNZT - HFAg	-1,368	-3,646 to 0,9090	No	ns	0,9318
S. gordonii:TNZT - HFAg vs. *A. naeslundii*:TAV - Control	0,04167	-2,130 to 2,213	No	ns	>0,9999
S. gordonii:TNZT - HFAg vs. *A. naeslundii*:TAV - CaP	0,9417	-1,486 to 3,369	No	ns	>0,9999
S. gordonii:TNZT - HFAg vs. *A. naeslundii*:TAV - CaPAg	-0,01500	-2,186 to 2,156	No	ns	>0,9999
S. gordonii:TNZT - HFAg vs. *A. naeslundii*:TAV - HF	0,000	-2,171 to 2,171	No	ns	>0,9999

S. gordonii:TNZT - HFAg vs. *A. naeslundii*:TAV - HFAg	0,1967	-1,975 to 2,368	No	ns	>0,9999
S. gordonii:TNZT - HFAg vs. *A. naeslundii*:TNZT - Control	-0,04167	-2,213 to 2,130	No	ns	>0,9999
S. gordonii:TNZT - HFAg vs. *A. naeslundii*:TNZT - CaP	0,04167	-2,130 to 2,213	No	ns	>0,9999
S. gordonii:TNZT - HFAg vs. *A. naeslundii*:TNZT - CaPAg	-0,1367	-2,308 to 2,035	No	ns	>0,9999
S. gordonii:TNZT - HFAg vs. *A. naeslundii*:TNZT - HF	0,4117	-1,760 to 2,583	No	ns	>0,9999
S. gordonii:TNZT - HFAg vs. *A. naeslundii*:TNZT - HFAg	-0,3058	-2,734 to 2,122	No	ns	>0,9999
S. gordonii:TNZT - HFAg vs. *P. gingivalis*:TAV - Control	-0,7917	-2,963 to 1,380	No	ns	>0,9999
S. gordonii:TNZT - HFAg vs. *P. gingivalis*:TAV - CaP	-1,947	-4,118 to 0,2247	No	ns	0,1668
S. gordonii:TNZT - HFAg vs. *P. gingivalis*:TAV - CaPAg	-2,055	-4,226 to 0,1164	No	ns	0,0964
S. gordonii:TNZT - HFAg vs. *P. gingivalis*:TAV - HF	-1,227	-3,398 to 0,9447	No	ns	0,9687
S. gordonii:TNZT - HFAg vs. *P. gingivalis*:TAV - HFAg	-1,736	-4,014 to 0,5410	No	ns	0,5131
S. gordonii:TNZT - HFAg vs. *P. gingivalis*:TNZT - Control	-1,952	-4,123 to 0,2197	No	ns	0,1629
S. gordonii:TNZT - HFAg vs. *P. gingivalis*:TNZT - CaP	-1,437	-3,608 to 0,7347	No	ns	0,8166
***S. gordonii*:TNZT - HFAg vs. *P. gingivalis*:TNZT - CaPAg**	**-2,187**	**-4,358 to -0,01528**	**Yes**	*****	**0,0457**
S. gordonii:TNZT - HFAg vs. *P. gingivalis*:TNZT - HF	-1,512	-3,683 to 0,6597	No	ns	0,7228
S. gordonii:TNZT - HFAg vs. *P. gingivalis*:TNZT - HFAg	-1,714	-3,992 to 0,5630	No	ns	0,5443
S. sanguinis:TAV - Control vs. *S. sanguinis*:TAV - CaP	-0,2500	-2,421 to 1,921	No	ns	>0,9999
S. sanguinis:TAV - Control vs. *S. sanguinis*:TAV - CaPAg	-0,07667	-2,248 to 2,095	No	ns	>0,9999
S. sanguinis:TAV - Control vs. *S. sanguinis*:TAV - HF	-0,2967	-2,468 to 1,875	No	ns	>0,9999
S. sanguinis:TAV - Control vs. *S. sanguinis*:TAV - HFAg	-0,03500	-2,206 to 2,136	No	ns	>0,9999
S. sanguinis:TAV - Control vs. *S. sanguinis*:TNZT - Control	-0,05000	-2,221 to 2,121	No	ns	>0,9999
S. sanguinis:TAV - Control vs. *S. sanguinis*:TNZT - CaP	-0,9150	-3,086 to 1,256	No	ns	0,9998
S. sanguinis:TAV - Control vs. *S. sanguinis*:TNZT - CaPAg	-0,5067	-2,678 to 1,665	No	ns	>0,9999
S. sanguinis:TAV - Control vs. *S. sanguinis*:TNZT - HF	-0,2763	-2,554 to 2,001	No	ns	>0,9999
S. sanguinis:TAV - Control vs. *S. sanguinis*:TNZT - HFAg	-0,4083	-2,686 to 1,869	No	ns	>0,9999
S. sanguinis:TAV - Control vs. *A. naeslundii*:TAV - Control	1,002	-1,170 to 3,173	No	ns	0,9989
S. sanguinis:TAV - Control vs. *A. naeslundii*:TAV - CaP	1,902	-0,5260 to 4,329	No	ns	0,4468
S. sanguinis:TAV - Control vs. *A. naeslundii*:TAV - CaPAg	0,9450	-1,226 to 3,116	No	ns	0,9997
S. sanguinis:TAV - Control vs. *A. naeslundii*:TAV - HF	0,9600	-1,211 to 3,131	No	ns	0,9995
S. sanguinis:TAV - Control vs. *A. naeslundii*:TAV - HFAg	1,157	-1,015 to 3,328	No	ns	0,9866
S. sanguinis:TAV - Control vs. *A. naeslundii*:TNZT - Control	0,9183	-1,253 to 3,090	No	ns	0,9998
S. sanguinis:TAV - Control vs. *A. naeslundii*:TNZT - CaP	1,002	-1,170 to 3,173	No	ns	0,9989
S. sanguinis:TAV - Control vs. *A. naeslundii*:TNZT - CaPAg	0,8233	-1,348 to 2,995	No	ns	>0,9999
S. sanguinis:TAV - Control vs. *A. naeslundii*:TNZT - HF	1,372	-0,7997 to 3,543	No	ns	0,8820
S. sanguinis:TAV - Control vs. *A. naeslundii*:TNZT - HFAg	0,6542	-1,774 to 3,082	No	ns	>0,9999
S. sanguinis:TAV - Control vs. *P. gingivalis*:TAV - Control	0,1683	-2,003 to 2,340	No	ns	>0,9999
S. sanguinis:TAV - Control vs. *P. gingivalis*:TAV - CaP	-0,9867	-3,158 to 1,185	No	ns	0,9992
S. sanguinis:TAV - Control vs. *P. gingivalis*:TAV - CaPAg	-1,095	-3,266 to 1,076	No	ns	0,9945
S. sanguinis:TAV - Control vs. *P. gingivalis*:TAV - HF	-0,2667	-2,438 to 1,905	No	ns	>0,9999
S. sanguinis:TAV - Control vs. *P. gingivalis*:TAV - HFAg	-0,7763	-3,054 to 1,501	No	ns	>0,9999
S. sanguinis:TAV - Control vs. *P. gingivalis*:TNZT - Control	-0,9917	-3,163 to 1,180	No	ns	0,9991
S. sanguinis:TAV - Control vs. *P. gingivalis*:TNZT - CaP	-0,4767	-2,648 to 1,695	No	ns	>0,9999
S. sanguinis:TAV - Control vs. *P. gingivalis*:TNZT - CaPAg	-1,227	-3,398 to 0,9447	No	ns	0,9687
S. sanguinis:TAV - Control vs. *P. gingivalis*:TNZT - HF	-0,5517	-2,723 to 1,620	No	ns	>0,9999
S. sanguinis:TAV - Control vs. *P. gingivalis*:TNZT - HFAg	-0,7543	-3,032 to 1,523	No	ns	>0,9999
S. sanguinis:TAV - CaP vs. *S. sanguinis*:TAV - CaPAg	0,1733	-1,998 to 2,345	No	ns	>0,9999
S. sanguinis:TAV - CaP vs. *S. sanguinis*:TAV - HF	-0,04667	-2,218 to 2,125	No	ns	>0,9999
S. sanguinis:TAV - CaP vs. *S. sanguinis*:TAV - HFAg	0,2150	-1,956 to 2,386	No	ns	>0,9999
S. sanguinis:TAV - CaP vs. *S. sanguinis*:TNZT - Control	0,2000	-1,971 to 2,371	No	ns	>0,9999
S. sanguinis:TAV - CaP vs. *S. sanguinis*:TNZT - CaP	-0,6650	-2,836 to 1,506	No	ns	>0,9999
S. sanguinis:TAV - CaP vs. *S. sanguinis*:TNZT - CaPAg	-0,2567	-2,428 to 1,915	No	ns	>0,9999
S. sanguinis:TAV - CaP vs. *S. sanguinis*:TNZT - HF	-0,02633	-2,304 to 2,251	No	ns	>0,9999
S. sanguinis:TAV - CaP vs. *S. sanguinis*:TNZT - HFAg	-0,1583	-2,436 to 2,119	No	ns	>0,9999
S. sanguinis:TAV - CaP vs. *A. naeslundii*:TAV - Control	1,252	-0,9197 to 3,423	No	ns	0,9591
S. sanguinis:TAV - CaP vs. *A. naeslundii*:TAV - CaP	2,152	-0,2760 to 4,579	No	ns	0,1852
S. sanguinis:TAV - CaP vs. *A. naeslundii*:TAV - CaPAg	1,195	-0,9764 to 3,366	No	ns	0,9783
S. sanguinis:TAV - CaP vs. *A. naeslundii*:TAV - HF	1,210	-0,9614 to 3,381	No	ns	0,9741
S. sanguinis:TAV - CaP vs. *A. naeslundii*:TAV - HFAg	1,407	-0,7647 to 3,578	No	ns	0,8488
S. sanguinis:TAV - CaP vs. *A. naeslundii*:TNZT - Control	1,168	-1,003 to 3,340	No	ns	0,9844
S. sanguinis:TAV - CaP vs. *A. naeslundii*:TNZT - CaP	1,252	-0,9197 to 3,423	No	ns	0,9591
S. sanguinis:TAV - CaP vs. *A. naeslundii*:TNZT - CaPAg	1,073	-1,098 to 3,245	No	ns	0,9961
S. sanguinis:TAV - CaP vs. *A. naeslundii*:TNZT - HF	1,622	-0,5497 to 3,793	No	ns	0,5635
S. sanguinis:TAV - CaP vs. *A. naeslundii*:TNZT - HFAg	0,9042	-1,524 to 3,332	No	ns	>0,9999
S. sanguinis:TAV - CaP vs. *P. gingivalis*:TAV - Control	0,4183	-1,753 to 2,590	No	ns	>0,9999
S. sanguinis:TAV - CaP vs. *P. gingivalis*:TAV - CaP	-0,7367	-2,908 to 1,435	No	ns	>0,9999
S. sanguinis:TAV - CaP vs. *P. gingivalis*:TAV - CaPAg	-0,8450	-3,016 to 1,326	No	ns	>0,9999
S. sanguinis:TAV - CaP vs. *P. gingivalis*:TAV - HF	-0,01667	-2,188 to 2,155	No	ns	>0,9999
S. sanguinis:TAV - CaP vs. *P. gingivalis*:TAV - HFAg	-0,5263	-2,804 to 1,751	No	ns	>0,9999
S. sanguinis:TAV - CaP vs. *P. gingivalis*:TNZT - Control	-0,7417	-2,913 to 1,430	No	ns	>0,9999
S. sanguinis:TAV - CaP vs. *P. gingivalis*:TNZT - CaP	-0,2267	-2,398 to 1,945	No	ns	>0,9999
S. sanguinis:TAV - CaP vs. *P. gingivalis*:TNZT - CaPAg	-0,9767	-3,148 to 1,195	No	ns	0,9993
S. sanguinis:TAV - CaP vs. *P. gingivalis*:TNZT - HF	-0,3017	-2,473 to 1,870	No	ns	>0,9999

S. sanguinis:TAV - CaP vs. *P. gingivalis*:TNZT - HFAg	-0,5043	-2,782 to 1,773	No	ns	>0,9999
S. sanguinis:TAV - CaPAg vs. *S. sanguinis*:TAV - HF	-0,2200	-2,391 to 1,951	No	ns	>0,9999
S. sanguinis:TAV - CaPAg vs. *S. sanguinis*:TAV - HFAg	0,04167	-2,130 to 2,213	No	ns	>0,9999
S. sanguinis:TAV - CaPAg vs. *S. sanguinis*:TNZT - Control	0,02667	-2,145 to 2,198	No	ns	>0,9999
S. sanguinis:TAV - CaPAg vs. *S. sanguinis*:TNZT - CaP	-0,8383	-3,010 to 1,333	No	ns	>0,9999
S. sanguinis:TAV - CaPAg vs. *S. sanguinis*:TNZT - CaPAg	-0,4300	-2,601 to 1,741	No	ns	>0,9999
S. sanguinis:TAV - CaPAg vs. *S. sanguinis*:TNZT - HF	-0,1997	-2,477 to 2,078	No	ns	>0,9999
S. sanguinis:TAV - CaPAg vs. *S. sanguinis*:TNZT - HFAg	-0,3317	-2,609 to 1,946	No	ns	>0,9999
S. sanguinis:TAV - CaPAg vs. *A. naeslundii*:TAV - Control	1,078	-1,093 to 3,250	No	ns	0,9957
S. sanguinis:TAV - CaPAg vs. *A. naeslundii*:TAV - CaP	1,978	-0,4494 to 4,406	No	ns	0,3530
S. sanguinis:TAV - CaPAg vs. *A. naeslundii*:TAV - CaPAg	1,022	-1,150 to 3,193	No	ns	0,9984
S. sanguinis:TAV - CaPAg vs. *A. naeslundii*:TAV - HF	1,037	-1,135 to 3,208	No	ns	0,9979
S. sanguinis:TAV - CaPAg vs. *A. naeslundii*:TAV - HFAg	1,233	-0,9381 to 3,405	No	ns	0,9663
S. sanguinis:TAV - CaPAg vs. *A. naeslundii*:TNZT - Control	0,9950	-1,176 to 3,166	No	ns	0,9990
S. sanguinis:TAV - CaPAg vs. *A. naeslundii*:TNZT - CaP	1,078	-1,093 to 3,250	No	ns	0,9957
S. sanguinis:TAV - CaPAg vs. *A. naeslundii*:TNZT - CaPAg	0,9000	-1,271 to 3,071	No	ns	0,9999
S. sanguinis:TAV - CaPAg vs. *A. naeslundii*:TNZT - HF	1,448	-0,7231 to 3,620	No	ns	0,8032
S. sanguinis:TAV - CaPAg vs. *A. naeslundii*:TNZT - HFAg	0,7308	-1,697 to 3,159	No	ns	>0,9999
S. sanguinis:TAV - CaPAg vs. *P. gingivalis*:TAV - Control	0,2450	-1,926 to 2,416	No	ns	>0,9999
S. sanguinis:TAV - CaPAg vs. *P. gingivalis*:TAV - CaP	-0,9100	-3,081 to 1,261	No	ns	0,9998
S. sanguinis:TAV - CaPAg vs. *P. gingivalis*:TAV - CaPAg	-1,018	-3,190 to 1,153	No	ns	0,9985
S. sanguinis:TAV - CaPAg vs. *P. gingivalis*:TAV - HF	-0,1900	-2,361 to 1,981	No	ns	>0,9999
S. sanguinis:TAV - CaPAg vs. *P. gingivalis*:TAV - HFAg	-0,6997	-2,977 to 1,578	No	ns	>0,9999
S. sanguinis:TAV - CaPAg vs. *P. gingivalis*:TNZT - Control	-0,9150	-3,086 to 1,256	No	ns	0,9998
S. sanguinis:TAV - CaPAg vs. *P. gingivalis*:TNZT - CaP	-0,4000	-2,571 to 1,771	No	ns	>0,9999
S. sanguinis:TAV - CaPAg vs. *P. gingivalis*:TNZT - CaPAg	-1,150	-3,321 to 1,021	No	ns	0,9877
S. sanguinis:TAV - CaPAg vs. *P. gingivalis*:TNZT - HF	-0,4750	-2,646 to 1,696	No	ns	>0,9999
S. sanguinis:TAV - CaPAg vs. *P. gingivalis*:TNZT - HFAg	-0,6777	-2,955 to 1,600	No	ns	>0,9999
S. sanguinis:TAV - HF vs. *S. sanguinis*:TAV - HFAg	0,2617	-1,910 to 2,433	No	ns	>0,9999
S. sanguinis:TAV - HF vs. *S. sanguinis*:TNZT - Control	0,2467	-1,925 to 2,418	No	ns	>0,9999
S. sanguinis:TAV - HF vs. *S. sanguinis*:TNZT - CaP	-0,6183	-2,790 to 1,553	No	ns	>0,9999
S. sanguinis:TAV - HF vs. *S. sanguinis*:TNZT - CaPAg	-0,2100	-2,381 to 1,961	No	ns	>0,9999
S. sanguinis:TAV - HF vs. *S. sanguinis*:TNZT - HF	0,02033	-2,257 to 2,298	No	ns	>0,9999
S. sanguinis:TAV - HF vs. *S. sanguinis*:TNZT - HFAg	-0,1117	-2,389 to 2,166	No	ns	>0,9999
S. sanguinis:TAV - HF vs. *A. naeslundii*:TAV - Control	1,298	-0,8731 to 3,470	No	ns	0,9356
S. sanguinis:TAV - HF vs. *A. naeslundii*:TAV - CaP	2,198	-0,2294 to 4,626	No	ns	0,1518
S. sanguinis:TAV - HF vs. *A. naeslundii*:TAV - CaPAg	1,242	-0,9297 to 3,413	No	ns	0,9632
S. sanguinis:TAV - HF vs. *A. naeslundii*:TAV - HF	1,257	-0,9147 to 3,428	No	ns	0,9569
S. sanguinis:TAV - HF vs. *A. naeslundii*:TAV - HFAg	1,453	-0,7181 to 3,625	No	ns	0,7973
S. sanguinis:TAV - HF vs. *A. naeslundii*:TNZT - Control	1,215	-0,9564 to 3,386	No	ns	0,9725
S. sanguinis:TAV - HF vs. *A. naeslundii*:TNZT - CaP	1,298	-0,8731 to 3,470	No	ns	0,9356
S. sanguinis:TAV - HF vs. *A. naeslundii*:TNZT - CaPAg	1,120	-1,051 to 3,291	No	ns	0,9919
S. sanguinis:TAV - HF vs. *A. naeslundii*:TNZT - HF	1,668	-0,5031 to 3,840	No	ns	0,4942
S. sanguinis:TAV - HF vs. *A. naeslundii*:TNZT - HFAg	0,9508	-1,477 to 3,379	No	ns	>0,9999
S. sanguinis:TAV - HF vs. *P. gingivalis*:TAV - Control	0,4650	-1,706 to 2,636	No	ns	>0,9999
S. sanguinis:TAV - HF vs. *P. gingivalis*:TAV - CaP	-0,6900	-2,861 to 1,481	No	ns	>0,9999
S. sanguinis:TAV - HF vs. *P. gingivalis*:TAV - CaPAg	-0,7983	-2,970 to 1,373	No	ns	>0,9999
S. sanguinis:TAV - HF vs. *P. gingivalis*:TAV - HF	0,03000	-2,141 to 2,201	No	ns	>0,9999
S. sanguinis:TAV - HF vs. *P. gingivalis*:TAV - HFAg	-0,4797	-2,757 to 1,798	No	ns	>0,9999
S. sanguinis:TAV - HF vs. *P. gingivalis*:TNZT - Control	-0,6950	-2,866 to 1,476	No	ns	>0,9999
S. sanguinis:TAV - HF vs. *P. gingivalis*:TNZT - CaP	-0,1800	-2,351 to 1,991	No	ns	>0,9999
S. sanguinis:TAV - HF vs. *P. gingivalis*:TNZT - CaPAg	-0,9300	-3,101 to 1,241	No	ns	0,9998
S. sanguinis:TAV - HF vs. *P. gingivalis*:TNZT - HF	-0,2550	-2,426 to 1,916	No	ns	>0,9999
S. sanguinis:TAV - HF vs. *P. gingivalis*:TNZT - HFAg	-0,4577	-2,735 to 1,820	No	ns	>0,9999
S. sanguinis:TAV - HFAg vs. *S. sanguinis*:TNZT - Control	-0,01500	-2,186 to 2,156	No	ns	>0,9999
S. sanguinis:TAV - HFAg vs. *S. sanguinis*:TNZT - CaP	-0,8800	-3,051 to 1,291	No	ns	>0,9999
S. sanguinis:TAV - HFAg vs. *S. sanguinis*:TNZT - CaPAg	-0,4717	-2,643 to 1,700	No	ns	>0,9999
S. sanguinis:TAV - HFAg vs. *S. sanguinis*:TNZT - HF	-0,2413	-2,519 to 2,036	No	ns	>0,9999
S. sanguinis:TAV - HFAg vs. *S. sanguinis*:TNZT - HFAg	-0,3733	-2,651 to 1,904	No	ns	>0,9999
S. sanguinis:TAV - HFAg vs. *A. naeslundii*:TAV - Control	1,037	-1,135 to 3,208	No	ns	0,9979
S. sanguinis:TAV - HFAg vs. *A. naeslundii*:TAV - CaP	1,937	-0,4910 to 4,364	No	ns	0,4028
S. sanguinis:TAV - HFAg vs. *A. naeslundii*:TAV - CaPAg	0,9800	-1,191 to 3,151	No	ns	0,9993
S. sanguinis:TAV - HFAg vs. *A. naeslundii*:TAV - HF	0,9950	-1,176 to 3,166	No	ns	0,9990
S. sanguinis:TAV - HFAg vs. *A. naeslundii*:TAV - HFAg	1,192	-0,9797 to 3,363	No	ns	0,9791
S. sanguinis:TAV - HFAg vs. *A. naeslundii*:TNZT - Control	0,9533	-1,218 to 3,125	No	ns	0,9996
S. sanguinis:TAV - HFAg vs. *A. naeslundii*:TNZT - CaP	1,037	-1,135 to 3,208	No	ns	0,9979
S. sanguinis:TAV - HFAg vs. *A. naeslundii*:TNZT - CaPAg	0,8583	-1,313 to 3,030	No	ns	>0,9999
S. sanguinis:TAV - HFAg vs. *A. naeslundii*:TNZT - HF	1,407	-0,7647 to 3,578	No	ns	0,8488
S. sanguinis:TAV - HFAg vs. *A. naeslundii*:TNZT - HFAg	0,6892	-1,739 to 3,117	No	ns	>0,9999
S. sanguinis:TAV - HFAg vs. *P. gingivalis*:TAV - Control	0,2033	-1,968 to 2,375	No	ns	>0,9999
S. sanguinis:TAV - HFAg vs. *P. gingivalis*:TAV - CaP	-0,9517	-3,123 to 1,220	No	ns	0,9996
S. sanguinis:TAV - HFAg vs. *P. gingivalis*:TAV - CaPAg	-1,060	-3,231 to 1,111	No	ns	0,9968

S. sanguinis:TAV - HFAg vs. *P. gingivalis*:TAV - HF	-0,2317	-2,403 to 1,940	No	ns	>0,9999
S. sanguinis:TAV - HFAg vs. *P. gingivalis*:TAV - HFAg	-0,7413	-3,019 to 1,536	No	ns	>0,9999
S. sanguinis:TAV - HFAg vs. *P. gingivalis*:TNZT - Control	-0,9567	-3,128 to 1,215	No	ns	0,9996
S. sanguinis:TAV - HFAg vs. *P. gingivalis*:TNZT - CaP	-0,4417	-2,613 to 1,730	No	ns	>0,9999
S. sanguinis:TAV - HFAg vs. *P. gingivalis*:TNZT - CaPAg	-1,192	-3,363 to 0,9797	No	ns	0,9791
S. sanguinis:TAV - HFAg vs. *P. gingivalis*:TNZT - HF	-0,5167	-2,688 to 1,655	No	ns	>0,9999
S. sanguinis:TAV - HFAg vs. *P. gingivalis*:TNZT - HFAg	-0,7193	-2,997 to 1,558	No	ns	>0,9999
S. sanguinis:TNZT - Control vs. *S. sanguinis*:TNZT - CaP	-0,8650	-3,036 to 1,306	No	ns	>0,9999
S. sanguinis:TNZT - Control vs. *S. sanguinis*:TNZT - CaPAg	-0,4567	-2,628 to 1,715	No	ns	>0,9999
S. sanguinis:TNZT - Control vs. *S. sanguinis*:TNZT - HF	-0,2263	-2,504 to 2,051	No	ns	>0,9999
S. sanguinis:TNZT - Control vs. *S. sanguinis*:TNZT - HFAg	-0,3583	-2,636 to 1,919	No	ns	>0,9999
S. sanguinis:TNZT - Control vs. *A. naeslundii*:TAV - Control	1,052	-1,120 to 3,223	No	ns	0,9973
S. sanguinis:TNZT - Control vs. *A. naeslundii*:TAV - CaP	1,952	-0,4760 to 4,379	No	ns	0,3845
S. sanguinis:TNZT - Control vs. *A. naeslundii*:TAV - CaPAg	0,9950	-1,176 to 3,166	No	ns	0,9990
S. sanguinis:TNZT - Control vs. *A. naeslundii*:TAV - HF	1,010	-1,161 to 3,181	No	ns	0,9987
S. sanguinis:TNZT - Control vs. *A. naeslundii*:TAV - HFAg	1,207	-0,9647 to 3,378	No	ns	0,9750
S. sanguinis:TNZT - Control vs. *A. naeslundii*:TNZT - Control	0,9683	-1,203 to 3,140	No	ns	0,9994
S. sanguinis:TNZT - Control vs. *A. naeslundii*:TNZT - CaP	1,052	-1,120 to 3,223	No	ns	0,9973
S. sanguinis:TNZT - Control vs. *A. naeslundii*:TNZT - CaPAg	0,8733	-1,298 to 3,045	No	ns	>0,9999
S. sanguinis:TNZT - Control vs. *A. naeslundii*:TNZT - HF	1,422	-0,7497 to 3,593	No	ns	0,8332
S. sanguinis:TNZT - Control vs. *A. naeslundii*:TNZT - HFAg	0,7042	-1,724 to 3,132	No	ns	>0,9999
S. sanguinis:TNZT - Control vs. *P. gingivalis*:TAV - Control	0,2183	-1,953 to 2,390	No	ns	>0,9999
S. sanguinis:TNZT - Control vs. *P. gingivalis*:TAV - CaP	-0,9367	-3,108 to 1,235	No	ns	0,9997
S. sanguinis:TNZT - Control vs. *P. gingivalis*:TAV - CaPAg	-1,045	-3,216 to 1,126	No	ns	0,9976
S. sanguinis:TNZT - Control vs. *P. gingivalis*:TAV - HF	-0,2167	-2,388 to 1,955	No	ns	>0,9999
S. sanguinis:TNZT - Control vs. *P. gingivalis*:TAV - HFAg	-0,7263	-3,004 to 1,551	No	ns	>0,9999
S. sanguinis:TNZT - Control vs. *P. gingivalis*:TNZT - Control	-0,9417	-3,113 to 1,230	No	ns	0,9997
S. sanguinis:TNZT - Control vs. *P. gingivalis*:TNZT - CaP	-0,4267	-2,598 to 1,745	No	ns	>0,9999
S. sanguinis:TNZT - Control vs. *P. gingivalis*:TNZT - CaPAg	-1,177	-3,348 to 0,9947	No	ns	0,9826
S. sanguinis:TNZT - Control vs. *P. gingivalis*:TNZT - HF	-0,5017	-2,673 to 1,670	No	ns	>0,9999
S. sanguinis:TNZT - Control vs. *P. gingivalis*:TNZT - HFAg	-0,7043	-2,982 to 1,573	No	ns	>0,9999
S. sanguinis:TNZT - CaP vs. *S. sanguinis*:TNZT - CaPAg	0,4083	-1,763 to 2,580	No	ns	>0,9999
S. sanguinis:TNZT - CaP vs. *S. sanguinis*:TNZT - HF	0,6387	-1,639 to 2,916	No	ns	>0,9999
S. sanguinis:TNZT - CaP vs. *S. sanguinis*:TNZT - HFAg	0,5067	-1,771 to 2,784	No	ns	>0,9999
S. sanguinis:TNZT - CaP vs. *A. naeslundii*:TAV - Control	1,917	-0,2547 to 4,088	No	ns	0,1920
***S. sanguinis*:TNZT - CaP vs. *A. naeslundii*:TAV - CaP**	**2,817**	**0,3890 to 5,244**	**Yes**	******	**0,0050**
S. sanguinis:TNZT - CaP vs. *A. naeslundii*:TAV - CaPAg	1,860	-0,3114 to 4,031	No	ns	0,2466
S. sanguinis:TNZT - CaP vs. *A. naeslundii*:TAV - HF	1,875	-0,2964 to 4,046	No	ns	0,2312
S. sanguinis:TNZT - CaP vs. *A. naeslundii*:TAV - HFAg	2,072	-0,09972 to 4,243	No	ns	0,0881
S. sanguinis:TNZT - CaP vs. *A. naeslundii*:TNZT - Control	1,833	-0,3381 to 4,005	No	ns	0,2756
S. sanguinis:TNZT - CaP vs. *A. naeslundii*:TNZT - CaP	1,917	-0,2547 to 4,088	No	ns	0,1920
S. sanguinis:TNZT - CaP vs. *A. naeslundii*:TNZT - CaPAg	1,738	-0,4331 to 3,910	No	ns	0,3944
***S. sanguinis*:TNZT - CaP vs. *A. naeslundii*:TNZT - HF**	**2,287**	**0,1153 to 4,458**	**Yes**	*****	**0,0245**
S. sanguinis:TNZT - CaP vs. *A. naeslundii*:TNZT - HFAg	1,569	-0,8585 to 3,997	No	ns	0,8520
S. sanguinis:TNZT - CaP vs. *P. gingivalis*:TAV - Control	1,083	-1,088 to 3,255	No	ns	0,9954
S. sanguinis:TNZT - CaP vs. *P. gingivalis*:TAV - CaP	-0,07167	-2,243 to 2,100	No	ns	>0,9999
S. sanguinis:TNZT - CaP vs. *P. gingivalis*:TAV - CaPAg	-0,1800	-2,351 to 1,991	No	ns	>0,9999
S. sanguinis:TNZT - CaP vs. *P. gingivalis*:TAV - HF	0,6483	-1,523 to 2,820	No	ns	>0,9999
S. sanguinis:TNZT - CaP vs. *P. gingivalis*:TAV - HFAg	0,1387	-2,139 to 2,416	No	ns	>0,9999
S. sanguinis:TNZT - CaP vs. *P. gingivalis*:TNZT - Control	-0,07667	-2,248 to 2,095	No	ns	>0,9999
S. sanguinis:TNZT - CaP vs. *P. gingivalis*:TNZT - CaP	0,4383	-1,733 to 2,610	No	ns	>0,9999
S. sanguinis:TNZT - CaP vs. *P. gingivalis*:TNZT - CaPAg	-0,3117	-2,483 to 1,860	No	ns	>0,9999
S. sanguinis:TNZT - CaP vs. *P. gingivalis*:TNZT - HF	0,3633	-1,808 to 2,535	No	ns	>0,9999
S. sanguinis:TNZT - CaP vs. *P. gingivalis*:TNZT - HFAg	0,1607	-2,117 to 2,438	No	ns	>0,9999
S. sanguinis:TNZT - CaPAg vs. *S. sanguinis*:TNZT - HF	0,2303	-2,047 to 2,508	No	ns	>0,9999
S. sanguinis:TNZT - CaPAg vs. *S. sanguinis*:TNZT - HFAg	0,09833	-2,179 to 2,376	No	ns	>0,9999
S. sanguinis:TNZT - CaPAg vs. *A. naeslundii*:TAV - Control	1,508	-0,6631 to 3,680	No	ns	0,7273
S. sanguinis:TNZT - CaPAg vs. *A. naeslundii*:TAV - CaP	2,408	-0,01935 to 4,836	No	ns	0,0554
S. sanguinis:TNZT - CaPAg vs. *A. naeslundii*:TAV - CaPAg	1,452	-0,7197 to 3,623	No	ns	0,7993
S. sanguinis:TNZT - CaPAg vs. *A. naeslundii*:TAV - HF	1,467	-0,7047 to 3,638	No	ns	0,7812
S. sanguinis:TNZT - CaPAg vs. *A. naeslundii*:TAV - HFAg	1,663	-0,5081 to 3,835	No	ns	0,5015
S. sanguinis:TNZT - CaPAg vs. *A. naeslundii*:TNZT - Control	1,425	-0,7464 to 3,596	No	ns	0,8296
S. sanguinis:TNZT - CaPAg vs. *A. naeslundii*:TNZT - CaP	1,508	-0,6631 to 3,680	No	ns	0,7273
S. sanguinis:TNZT - CaPAg vs. *A. naeslundii*:TNZT - CaPAg	1,330	-0,8414 to 3,501	No	ns	0,9151
S. sanguinis:TNZT - CaPAg vs. *A. naeslundii*:TNZT - HF	1,878	-0,2931 to 4,050	No	ns	0,2279
S. sanguinis:TNZT - CaPAg vs. *A. naeslundii*:TNZT - HFAg	1,161	-1,267 to 3,589	No	ns	0,9978
S. sanguinis:TNZT - CaPAg vs. *P. gingivalis*:TAV - Control	0,6750	-1,496 to 2,846	No	ns	>0,9999
S. sanguinis:TNZT - CaPAg vs. *P. gingivalis*:TAV - CaP	-0,4800	-2,651 to 1,691	No	ns	>0,9999
S. sanguinis:TNZT - CaPAg vs. *P. gingivalis*:TAV - CaPAg	-0,5883	-2,760 to 1,583	No	ns	>0,9999
S. sanguinis:TNZT - CaPAg vs. *P. gingivalis*:TAV - HF	0,2400	-1,931 to 2,411	No	ns	>0,9999
S. sanguinis:TNZT - CaPAg vs. *P. gingivalis*:TAV - HFAg	-0,2697	-2,547 to 2,008	No	ns	>0,9999
S. sanguinis:TNZT - CaPAg vs. *P. gingivalis*:TNZT - Control	-0,4850	-2,656 to 1,686	No	ns	>0,9999

S. sanguinis:TNZT - CaPAg vs. *P. gingivalis*:TNZT - CaP	0,03000	-2,141 to 2,201	No	ns	>0,9999
S. sanguinis:TNZT - CaPAg vs. *P. gingivalis*:TNZT - CaPAg	-0,7200	-2,891 to 1,451	No	ns	>0,9999
S. sanguinis:TNZT - CaPAg vs. *P. gingivalis*:TNZT - HF	-0,04500	-2,216 to 2,126	No	ns	>0,9999
S. sanguinis:TNZT - CaPAg vs. *P. gingivalis*:TNZT - HFAg	-0,2477	-2,525 to 2,030	No	ns	>0,9999
S. sanguinis:TNZT - HF vs. *S. sanguinis*:TNZT - HFAg	-0,1320	-2,511 to 2,247	No	ns	>0,9999
S. sanguinis:TNZT - HF vs. *A. naeslundii*:TAV - Control	1,278	-0,9994 to 3,555	No	ns	0,9714
S. sanguinis:TNZT - HF vs. *A. naeslundii*:TAV - CaP	2,178	-0,3449 to 4,701	No	ns	0,2317
S. sanguinis:TNZT - HF vs. *A. naeslundii*:TAV - CaPAg	1,221	-1,056 to 3,499	No	ns	0,9851
S. sanguinis:TNZT - HF vs. *A. naeslundii*:TAV - HF	1,236	-1,041 to 3,514	No	ns	0,9822
S. sanguinis:TNZT - HF vs. *A. naeslundii*:TAV - HFAg	1,433	-0,8444 to 3,710	No	ns	0,8867
S. sanguinis:TNZT - HF vs. *A. naeslundii*:TNZT - Control	1,195	-1,083 to 3,472	No	ns	0,9894
S. sanguinis:TNZT - HF vs. *A. naeslundii*:TNZT - CaP	1,278	-0,9994 to 3,555	No	ns	0,9714
S. sanguinis:TNZT - HF vs. *A. naeslundii*:TNZT - CaPAg	1,100	-1,178 to 3,377	No	ns	0,9974
S. sanguinis:TNZT - HF vs. *A. naeslundii*:TNZT - HF	1,648	-0,6294 to 3,925	No	ns	0,6382
S. sanguinis:TNZT - HF vs. *A. naeslundii*:TNZT - HFAg	0,9305	-1,592 to 3,453	No	ns	>0,9999
S. sanguinis:TNZT - HF vs. *P. gingivalis*:TAV - Control	0,4447	-1,833 to 2,722	No	ns	>0,9999
S. sanguinis:TNZT - HF vs. *P. gingivalis*:TAV - CaP	-0,7103	-2,988 to 1,567	No	ns	>0,9999
S. sanguinis:TNZT - HF vs. *P. gingivalis*:TAV - CaPAg	-0,8187	-3,096 to 1,459	No	ns	>0,9999
S. sanguinis:TNZT - HF vs. *P. gingivalis*:TAV - HF	0,009667	-2,268 to 2,287	No	ns	>0,9999
S. sanguinis:TNZT - HF vs. *P. gingivalis*:TAV - HFAg	-0,5000	-2,879 to 1,879	No	ns	>0,9999
S. sanguinis:TNZT - HF vs. *P. gingivalis*:TNZT - Control	-0,7153	-2,993 to 1,562	No	ns	>0,9999
S. sanguinis:TNZT - HF vs. *P. gingivalis*:TNZT - CaP	-0,2003	-2,478 to 2,077	No	ns	>0,9999
S. sanguinis:TNZT - HF vs. *P. gingivalis*:TNZT - CaPAg	-0,9503	-3,228 to 1,327	No	ns	0,9999
S. sanguinis:TNZT - HF vs. *P. gingivalis*:TNZT - HF	-0,2753	-2,553 to 2,002	No	ns	>0,9999
S. sanguinis:TNZT - HF vs. *P. gingivalis*:TNZT - HFAg	-0,4780	-2,857 to 1,901	No	ns	>0,9999
S. sanguinis:TNZT - HFAg vs. *A. naeslundii*:TAV - Control	1,410	-0,8674 to 3,687	No	ns	0,9045
S. sanguinis:TNZT - HFAg vs. *A. naeslundii*:TAV - CaP	2,310	-0,2129 to 4,833	No	ns	0,1363
S. sanguinis:TNZT - HFAg vs. *A. naeslundii*:TAV - CaPAg	1,353	-0,9240 to 3,631	No	ns	0,9402
S. sanguinis:TNZT - HFAg vs. *A. naeslundii*:TAV - HF	1,368	-0,9090 to 3,646	No	ns	0,9318
S. sanguinis:TNZT - HFAg vs. *A. naeslundii*:TAV - HFAg	1,565	-0,7124 to 3,842	No	ns	0,7488
S. sanguinis:TNZT - HFAg vs. *A. naeslundii*:TNZT - Control	1,327	-0,9507 to 3,604	No	ns	0,9532
S. sanguinis:TNZT - HFAg vs. *A. naeslundii*:TNZT - CaP	1,410	-0,8674 to 3,687	No	ns	0,9045
S. sanguinis:TNZT - HFAg vs. *A. naeslundii*:TNZT - CaPAg	1,232	-1,046 to 3,509	No	ns	0,9831
S. sanguinis:TNZT - HFAg vs. *A. naeslundii*:TNZT - HF	1,780	-0,4974 to 4,057	No	ns	0,4522
S. sanguinis:TNZT - HFAg vs. *A. naeslundii*:TNZT - HFAg	1,063	-1,460 to 3,585	No	ns	0,9998
S. sanguinis:TNZT - HFAg vs. *P. gingivalis*:TAV - Control	0,5767	-1,701 to 2,854	No	ns	>0,9999
S. sanguinis:TNZT - HFAg vs. *P. gingivalis*:TAV - CaP	-0,5783	-2,856 to 1,699	No	ns	>0,9999
S. sanguinis:TNZT - HFAg vs. *P. gingivalis*:TAV - CaPAg	-0,6867	-2,964 to 1,591	No	ns	>0,9999
S. sanguinis:TNZT - HFAg vs. *P. gingivalis*:TAV - HF	0,1417	-2,136 to 2,419	No	ns	>0,9999
S. sanguinis:TNZT - HFAg vs. *P. gingivalis*:TAV - HFAg	-0,3680	-2,747 to 2,011	No	ns	>0,9999
S. sanguinis:TNZT - HFAg vs. *P. gingivalis*:TNZT - Control	-0,5833	-2,861 to 1,694	No	ns	>0,9999
S. sanguinis:TNZT - HFAg vs. *P. gingivalis*:TNZT - CaP	-0,06833	-2,346 to 2,209	No	ns	>0,9999
S. sanguinis:TNZT - HFAg vs. *P. gingivalis*:TNZT - CaPAg	-0,8183	-3,096 to 1,459	No	ns	>0,9999
S. sanguinis:TNZT - HFAg vs. *P. gingivalis*:TNZT - HF	-0,1433	-2,421 to 2,134	No	ns	>0,9999
S. sanguinis:TNZT - HFAg vs. *P. gingivalis*:TNZT - HFAg	-0,3460	-2,725 to 2,033	No	ns	>0,9999
A. naeslundii:TAV - Control vs. *A. naeslundii*:TAV - CaP	0,9000	-1,528 to 3,328	No	ns	>0,9999
A. naeslundii:TAV - Control vs. *A. naeslundii*:TAV - CaPAg	-0,05667	-2,228 to 2,115	No	ns	>0,9999
A. naeslundii:TAV - Control vs. *A. naeslundii*:TAV - HF	-0,04167	-2,213 to 2,130	No	ns	>0,9999
A. naeslundii:TAV - Control vs. *A. naeslundii*:TAV - HFAg	0,1550	-2,016 to 2,326	No	ns	>0,9999
A. naeslundii:TAV - Control vs. *A. naeslundii*:TNZT - Control	-0,08333	-2,255 to 2,088	No	ns	>0,9999
A. naeslundii:TAV - Control vs. *A. naeslundii*:TNZT - CaP	0,000	-2,171 to 2,171	No	ns	>0,9999
A. naeslundii:TAV - Control vs. *A. naeslundii*:TNZT - CaPAg	-0,1783	-2,350 to 1,993	No	ns	>0,9999
A. naeslundii:TAV - Control vs. *A. naeslundii*:TNZT - HF	0,3700	-1,801 to 2,541	No	ns	>0,9999
A. naeslundii:TAV - Control vs. *A. naeslundii*:TNZT - HFAg	-0,3475	-2,775 to 2,080	No	ns	>0,9999
A. naeslundii:TAV - Control vs. *P. gingivalis*:TAV - Control	-0,8333	-3,005 to 1,338	No	ns	>0,9999
A. naeslundii:TAV - Control vs. *P. gingivalis*:TAV - CaP	-1,988	-4,160 to 0,1831	No	ns	0,1361
A. naeslundii:TAV - Control vs. *P. gingivalis*:TAV - CaPAg	-2,097	-4,268 to 0,07472	No	ns	0,0768
A. naeslundii:TAV - Control vs. *P. gingivalis*:TAV - HF	-1,268	-3,440 to 0,9031	No	ns	0,9516
A. naeslundii:TAV - Control vs. *P. gingivalis*:TAV - HFAg	-1,778	-4,055 to 0,4994	No	ns	0,4549
A. naeslundii:TAV - Control vs. *P. gingivalis*:TNZT - Control	-1,993	-4,165 to 0,1781	No	ns	0,1328
A. naeslundii:TAV - Control vs. *P. gingivalis*:TNZT - CaP	-1,478	-3,650 to 0,6931	No	ns	0,7666
***A. naeslundii*:TAV - Control vs. *P. gingivalis*:TNZT - CaPAg**	**-2,228**	**-4,400 to -0,05694**	**Yes**	*****	**0,0354**
A. naeslundii:TAV - Control vs. *P. gingivalis*:TNZT - HF	-1,553	-3,725 to 0,6181	No	ns	0,6644
A. naeslundii:TAV - Control vs. *P. gingivalis*:TNZT - HFAg	-1,756	-4,033 to 0,5214	No	ns	0,4854
A. naeslundii:TAV - CaP vs. *A. naeslundii*:TAV - CaPAg	-0,9567	-3,384 to 1,471	No	ns	>0,9999
A. naeslundii:TAV - CaP vs. *A. naeslundii*:TAV - HF	-0,9417	-3,369 to 1,486	No	ns	>0,9999
A. naeslundii:TAV - CaP vs. *A. naeslundii*:TAV - HFAg	-0,7450	-3,173 to 1,683	No	ns	>0,9999
A. naeslundii:TAV - CaP vs. *A. naeslundii*:TNZT - Control	-0,9833	-3,411 to 1,444	No	ns	>0,9999
A. naeslundii:TAV - CaP vs. *A. naeslundii*:TNZT - CaP	-0,9000	-3,328 to 1,528	No	ns	>0,9999
A. naeslundii:TAV - CaP vs. *A. naeslundii*:TNZT - CaPAg	-1,078	-3,506 to 1,349	No	ns	0,9995
A. naeslundii:TAV - CaP vs. *A. naeslundii*:TNZT - HF	-0,5300	-2,958 to 1,898	No	ns	>0,9999
A. naeslundii:TAV - CaP vs. *A. naeslundii*:TNZT - HFAg	-1,248	-3,907 to 1,412	No	ns	0,9985

A. naeslundii:TAV - CaP vs. *P. gingivalis*:TAV - Control	-1,733	-4,161 to 0,6944	No	ns	0,6687
**A. naeslundii:TAV - CaP vs. P. gingivalis:TAV - CaP**	**-2,888**	**-5,316 to -0,4606**	**Yes**	******	**0,0031**
**A. naeslundii:TAV - CaP vs. P. gingivalis:TAV - CaPAg**	**-2,997**	**-5,424 to -0,5690**	**Yes**	******	**0,0015**
A. naeslundii:TAV - CaP vs. *P. gingivalis*:TAV - HF	-2,168	-4,596 to 0,2594	No	ns	0,1727
**A. naeslundii:TAV - CaP vs. P. gingivalis:TAV - HFAg**	**-2,678**	**-5,201 to -0,1551**	**Yes**	*****	**0,0218**
**A. naeslundii:TAV - CaP vs. P. gingivalis:TNZT - Control**	**-2,893**	**-5,321 to -0,4656**	**Yes**	******	**0,0030**
A. naeslundii:TAV - CaP vs. *P. gingivalis*:TNZT - CaP	-2,378	-4,806 to 0,04935	No	ns	0,0646
**A. naeslundii:TAV - CaP vs. P. gingivalis:TNZT - CaPAg**	**-3,128**	**-5,556 to -0,7006**	**Yes**	*******	**0,0006**
**A. naeslundii:TAV - CaP vs. P. gingivalis:TNZT - HF**	**-2,453**	**-4,881 to -0,02565**	**Yes**	*****	**0,0436**
**A. naeslundii:TAV - CaP vs. P. gingivalis:TNZT - HFAg**	**-2,656**	**-5,179 to -0,1331**	**Yes**	*****	**0,0247**
A. naeslundii:TAV - CaPAg vs. *A. naeslundii*:TAV - HF	0,01500	-2,156 to 2,186	No	ns	>0,9999
A. naeslundii:TAV - CaPAg vs. *A. naeslundii*:TAV - HFAg	0,2117	-1,960 to 2,383	No	ns	>0,9999
A. naeslundii:TAV - CaPAg vs. *A. naeslundii*:TNZT - Control	-0,02667	-2,198 to 2,145	No	ns	>0,9999
A. naeslundii:TAV - CaPAg vs. *A. naeslundii*:TNZT - CaP	0,05667	-2,115 to 2,228	No	ns	>0,9999
A. naeslundii:TAV - CaPAg vs. *A. naeslundii*:TNZT - CaPAg	-0,1217	-2,293 to 2,050	No	ns	>0,9999
A. naeslundii:TAV - CaPAg vs. *A. naeslundii*:TNZT - HF	0,4267	-1,745 to 2,598	No	ns	>0,9999
A. naeslundii:TAV - CaPAg vs. *A. naeslundii*:TNZT - HFAg	-0,2908	-2,719 to 2,137	No	ns	>0,9999
A. naeslundii:TAV - CaPAg vs. *P. gingivalis*:TAV - Control	-0,7767	-2,948 to 1,395	No	ns	>0,9999
A. naeslundii:TAV - CaPAg vs. *P. gingivalis*:TAV - CaP	-1,932	-4,103 to 0,2397	No	ns	0,1791
A. naeslundii:TAV - CaPAg vs. *P. gingivalis*:TAV - CaPAg	-2,040	-4,211 to 0,1314	No	ns	0,1044
A. naeslundii:TAV - CaPAg vs. *P. gingivalis*:TAV - HF	-1,212	-3,383 to 0,9597	No	ns	0,9736
A. naeslundii:TAV - CaPAg vs. *P. gingivalis*:TAV - HFAg	-1,721	-3,999 to 0,5560	No	ns	0,5343
A. naeslundii:TAV - CaPAg vs. *P. gingivalis*:TNZT - Control	-1,937	-4,108 to 0,2347	No	ns	0,1749
A. naeslundii:TAV - CaPAg vs. *P. gingivalis*:TNZT - CaP	-1,422	-3,593 to 0,7497	No	ns	0,8332
**A. naeslundii:TAV - CaPAg vs. P. gingivalis:TNZT - CaPAg**	**-2,172**	**-4,343 to -0,0002765**	**Yes**	*****	**0,0499**
A. naeslundii:TAV - CaPAg vs. *P. gingivalis*:TNZT - HF	-1,497	-3,668 to 0,6747	No	ns	0,7429
A. naeslundii:TAV - CaPAg vs. *P. gingivalis*:TNZT - HFAg	-1,699	-3,977 to 0,5780	No	ns	0,5656
A. naeslundii:TAV - HF vs. *A. naeslundii*:TAV - HFAg	0,1967	-1,975 to 2,368	No	ns	>0,9999
A. naeslundii:TAV - HF vs. *A. naeslundii*:TNZT - Control	-0,04167	-2,213 to 2,130	No	ns	>0,9999
A. naeslundii:TAV - HF vs. *A. naeslundii*:TNZT - CaP	0,04167	-2,130 to 2,213	No	ns	>0,9999
A. naeslundii:TAV - HF vs. *A. naeslundii*:TNZT - CaPAg	-0,1367	-2,308 to 2,035	No	ns	>0,9999
A. naeslundii:TAV - HF vs. *A. naeslundii*:TNZT - HF	0,4117	-1,760 to 2,583	No	ns	>0,9999
A. naeslundii:TAV - HF vs. *A. naeslundii*:TNZT - HFAg	-0,3058	-2,734 to 2,122	No	ns	>0,9999
A. naeslundii:TAV - HF vs. *P. gingivalis*:TAV - Control	-0,7917	-2,963 to 1,380	No	ns	>0,9999
A. naeslundii:TAV - HF vs. *P. gingivalis*:TAV - CaP	-1,947	-4,118 to 0,2247	No	ns	0,1668
A. naeslundii:TAV - HF vs. *P. gingivalis*:TAV - CaPAg	-2,055	-4,226 to 0,1164	No	ns	0,0964
A. naeslundii:TAV - HF vs. *P. gingivalis*:TAV - HF	-1,227	-3,398 to 0,9447	No	ns	0,9687
A. naeslundii:TAV - HF vs. *P. gingivalis*:TAV - HFAg	-1,736	-4,014 to 0,5410	No	ns	0,5131
A. naeslundii:TAV - HF vs. *P. gingivalis*:TNZT - Control	-1,952	-4,123 to 0,2197	No	ns	0,1629
A. naeslundii:TAV - HF vs. *P. gingivalis*:TNZT - CaP	-1,437	-3,608 to 0,7347	No	ns	0,8166
**A. naeslundii:TAV - HF vs. P. gingivalis:TNZT - CaPAg**	**-2,187**	**-4,358 to -0,01528**	**Yes**	*****	**0,0457**
A. naeslundii:TAV - HF vs. *P. gingivalis*:TNZT - HF	-1,512	-3,683 to 0,6597	No	ns	0,7228
A. naeslundii:TAV - HF vs. *P. gingivalis*:TNZT - HFAg	-1,714	-3,992 to 0,5630	No	ns	0,5443
A. naeslundii:TAV - HFAg vs. *A. naeslundii*:TNZT - Control	-0,2383	-2,410 to 1,933	No	ns	>0,9999
A. naeslundii:TAV - HFAg vs. *A. naeslundii*:TNZT - CaP	-0,1550	-2,326 to 2,016	No	ns	>0,9999
A. naeslundii:TAV - HFAg vs. *A. naeslundii*:TNZT - CaPAg	-0,3333	-2,505 to 1,838	No	ns	>0,9999
A. naeslundii:TAV - HFAg vs. *A. naeslundii*:TNZT - HF	0,2150	-1,956 to 2,386	No	ns	>0,9999
A. naeslundii:TAV - HFAg vs. *A. naeslundii*:TNZT - HFAg	-0,5025	-2,930 to 1,925	No	ns	>0,9999
A. naeslundii:TAV - HFAg vs. *P. gingivalis*:TAV - Control	-0,9883	-3,160 to 1,183	No	ns	0,9992
A. naeslundii:TAV - HFAg vs. *P. gingivalis*:TAV - CaP	-2,143	-4,315 to 0,02806	No	ns	0,0589
**A. naeslundii:TAV - HFAg vs. P. gingivalis:TAV - CaPAg**	**-2,252**	**-4,423 to -0,08028**	**Yes**	*****	**0,0306**
A. naeslundii:TAV - HFAg vs. *P. gingivalis*:TAV - HF	-1,423	-3,595 to 0,7481	No	ns	0,8314
A. naeslundii:TAV - HFAg vs. *P. gingivalis*:TAV - HFAg	-1,933	-4,210 to 0,3444	No	ns	0,2648
A. naeslundii:TAV - HFAg vs. *P. gingivalis*:TNZT - Control	-2,148	-4,320 to 0,02306	No	ns	0,0572
A. naeslundii:TAV - HFAg vs. *P. gingivalis*:TNZT - CaP	-1,633	-3,805 to 0,5381	No	ns	0,5461
**A. naeslundii:TAV - HFAg vs. P. gingivalis:TNZT - CaPAg**	**-2,383**	**-4,555 to -0,2119**	**Yes**	*****	**0,0129**
A. naeslundii:TAV - HFAg vs. *P. gingivalis*:TNZT - HF	-1,708	-3,880 to 0,4631	No	ns	0,4362
A. naeslundii:TAV - HFAg vs. *P. gingivalis*:TNZT - HFAg	-1,911	-4,188 to 0,3664	No	ns	0,2884
A. naeslundii:TNZT - Control vs. *A. naeslundii*:TNZT - CaP	0,08333	-2,088 to 2,255	No	ns	>0,9999
A. naeslundii:TNZT - Control vs. *A. naeslundii*:TNZT - CaPAg	-0,09500	-2,266 to 2,076	No	ns	>0,9999
A. naeslundii:TNZT - Control vs. *A. naeslundii*:TNZT - HF	0,4533	-1,718 to 2,625	No	ns	>0,9999
A. naeslundii:TNZT - Control vs. *A. naeslundii*:TNZT - HFAg	-0,2642	-2,692 to 2,164	No	ns	>0,9999
A. naeslundii:TNZT - Control vs. *P. gingivalis*:TAV - Control	-0,7500	-2,921 to 1,421	No	ns	>0,9999
A. naeslundii:TNZT - Control vs. *P. gingivalis*:TAV - CaP	-1,905	-4,076 to 0,2664	No	ns	0,2025
A. naeslundii:TNZT - Control vs. *P. gingivalis*:TAV - CaPAg	-2,013	-4,185 to 0,1581	No	ns	0,1199
A. naeslundii:TNZT - Control vs. *P. gingivalis*:TAV - HF	-1,185	-3,356 to 0,9864	No	ns	0,9807
A. naeslundii:TNZT - Control vs. *P. gingivalis*:TAV - HFAg	-1,695	-3,972 to 0,5827	No	ns	0,5723
A. naeslundii:TNZT - Control vs. *P. gingivalis*:TNZT - Control	-1,910	-4,081 to 0,2614	No	ns	0,1979
A. naeslundii:TNZT - Control vs. *P. gingivalis*:TNZT - CaP	-1,395	-3,566 to 0,7764	No	ns	0,8604
A. naeslundii:TNZT - Control vs. *P. gingivalis*:TNZT - CaPAg	-2,145	-4,316 to 0,02639	No	ns	0,0584
A. naeslundii:TNZT - Control vs. *P. gingivalis*:TNZT - HF	-1,470	-3,641 to 0,7014	No	ns	0,7771
A. naeslundii:TNZT - Control vs. *P. gingivalis*:TNZT - HFAg	-1,673	-3,950 to 0,6047	No	ns	0,6035

A. naeslundii:TNZT - CaP vs. *A. naeslundii*:TNZT - CaPAg	-0,1783	-2,350 to 1,993	No	ns	>0,9999
A. naeslundii:TNZT - CaP vs. *A. naeslundii*:TNZT - HF	0,3700	-1,801 to 2,541	No	ns	>0,9999
A. naeslundii:TNZT - CaP vs. *A. naeslundii*:TNZT - HFAg	-0,3475	-2,775 to 2,080	No	ns	>0,9999
A. naeslundii:TNZT - CaP vs. *P. gingivalis*:TAV - Control	-0,8333	-3,005 to 1,338	No	ns	>0,9999
A. naeslundii:TNZT - CaP vs. *P. gingivalis*:TAV - CaP	-1,988	-4,160 to 0,1831	No	ns	0,1361
A. naeslundii:TNZT - CaP vs. *P. gingivalis*:TAV - CaPAg	-2,097	-4,268 to 0,07472	No	ns	0,0768
A. naeslundii:TNZT - CaP vs. *P. gingivalis*:TAV - HF	-1,268	-3,440 to 0,9031	No	ns	0,9516
A. naeslundii:TNZT - CaP vs. *P. gingivalis*:TAV - HFAg	-1,778	-4,055 to 0,4994	No	ns	0,4549
A. naeslundii:TNZT - CaP vs. *P. gingivalis*:TNZT - Control	-1,993	-4,165 to 0,1781	No	ns	0,1328
A. naeslundii:TNZT - CaP vs. *P. gingivalis*:TNZT - CaP	-1,478	-3,650 to 0,6931	No	ns	0,7666
A. naeslundii**:TNZT - CaP vs. *****P. gingivalis*****:TNZT - CaPAg**	**-2,228**	**-4,400 to -0,05694**	**Yes**	*****	**0,0354**
A. naeslundii:TNZT - CaP vs. *P. gingivalis*:TNZT - HF	-1,553	-3,725 to 0,6181	No	ns	0,6644
A. naeslundii:TNZT - CaP vs. *P. gingivalis*:TNZT - HFAg	-1,756	-4,033 to 0,5214	No	ns	0,4854
A. naeslundii:TNZT - CaPAg vs. *A. naeslundii*:TNZT - HF	0,5483	-1,623 to 2,720	No	ns	>0,9999
A. naeslundii:TNZT - CaPAg vs. *A. naeslundii*:TNZT - HFAg	-0,1692	-2,597 to 2,259	No	ns	>0,9999
A. naeslundii:TNZT - CaPAg vs. *P. gingivalis*:TAV - Control	-0,6550	-2,826 to 1,516	No	ns	>0,9999
A. naeslundii:TNZT - CaPAg vs. *P. gingivalis*:TAV - CaP	-1,810	-3,981 to 0,3614	No	ns	0,3026
A. naeslundii:TNZT - CaPAg vs. *P. gingivalis*:TAV - CaPAg	-1,918	-4,090 to 0,2531	No	ns	0,1905
A. naeslundii:TNZT - CaPAg vs. *P. gingivalis*:TAV - HF	-1,090	-3,261 to 1,081	No	ns	0,9949
A. naeslundii:TNZT - CaPAg vs. *P. gingivalis*:TAV - HFAg	-1,600	-3,877 to 0,6777	No	ns	0,7042
A. naeslundii:TNZT - CaPAg vs. *P. gingivalis*:TNZT - Control	-1,815	-3,986 to 0,3564	No	ns	0,2967
A. naeslundii:TNZT - CaPAg vs. *P. gingivalis*:TNZT - CaP	-1,300	-3,471 to 0,8714	No	ns	0,9346
A. naeslundii:TNZT - CaPAg vs. *P. gingivalis*:TNZT - CaPAg	-2,050	-4,221 to 0,1214	No	ns	0,0990
A. naeslundii:TNZT - CaPAg vs. *P. gingivalis*:TNZT - HF	-1,375	-3,546 to 0,7964	No	ns	0,8791
A. naeslundii:TNZT - CaPAg vs. *P. gingivalis*:TNZT - HFAg	-1,578	-3,855 to 0,6997	No	ns	0,7328
A. naeslundii:TNZT - HF vs. *A. naeslundii*:TNZT - HFAg	-0,7175	-3,145 to 1,710	No	ns	>0,9999
A. naeslundii:TNZT - HF vs. *P. gingivalis*:TAV - Control	-1,203	-3,375 to 0,9681	No	ns	0,9760
A. naeslundii**:TNZT - HF vs. *****P. gingivalis*****:TAV - CaP**	**-2,358**	**-4,530 to -0,1869**	**Yes**	*****	**0,0153**
A. naeslundii**:TNZT - HF vs. *****P. gingivalis*****:TAV - CaPAg**	**-2,467**	**-4,638 to -0,2953**	**Yes**	******	**0,0072**
A. naeslundii:TNZT - HF vs. *P. gingivalis*:TAV - HF	-1,638	-3,810 to 0,5331	No	ns	0,5386
A. naeslundii:TNZT - HF vs. *P. gingivalis*:TAV - HFAg	-2,148	-4,425 to 0,1294	No	ns	0,1001
A. naeslundii**:TNZT - HF vs. *****P. gingivalis*****:TNZT - Control**	**-2,363**	**-4,535 to -0,1919**	**Yes**	*****	**0,0148**
A. naeslundii:TNZT - HF vs. *P. gingivalis*:TNZT - CaP	-1,848	-4,020 to 0,3231	No	ns	0,2590
A. naeslundii**:TNZT - HF vs. *****P. gingivalis*****:TNZT - CaPAg**	**-2,598**	**-4,770 to -0,4269**	**Yes**	******	**0,0028**
A. naeslundii:TNZT - HF vs. *P. gingivalis*:TNZT - HF	-1,923	-4,095 to 0,2481	No	ns	0,1862
A. naeslundii:TNZT - HF vs. *P. gingivalis*:TNZT - HFAg	-2,126	-4,403 to 0,1514	No	ns	0,1117
A. naeslundii:TNZT - HFAg vs. *P. gingivalis*:TAV - Control	-0,4858	-2,914 to 1,942	No	ns	>0,9999
A. naeslundii:TNZT - HFAg vs. *P. gingivalis*:TAV - CaP	-1,641	-4,069 to 0,7869	No	ns	0,7800
A. naeslundii:TNZT - HFAg vs. *P. gingivalis*:TAV - CaPAg	-1,749	-4,177 to 0,6785	No	ns	0,6482
A. naeslundii:TNZT - HFAg vs. *P. gingivalis*:TAV - HF	-0,9208	-3,349 to 1,507	No	ns	>0,9999
A. naeslundii:TNZT - HFAg vs. *P. gingivalis*:TAV - HFAg	-1,431	-3,953 to 1,092	No	ns	0,9671
A. naeslundii:TNZT - HFAg vs. *P. gingivalis*:TNZT - Control	-1,646	-4,074 to 0,7819	No	ns	0,7745
A. naeslundii:TNZT - HFAg vs. *P. gingivalis*:TNZT - CaP	-1,131	-3,559 to 1,297	No	ns	0,9987
A. naeslundii:TNZT - HFAg vs. *P. gingivalis*:TNZT - CaPAg	-1,881	-4,309 to 0,5469	No	ns	0,4737
A. naeslundii:TNZT - HFAg vs. *P. gingivalis*:TNZT - HF	-1,206	-3,634 to 1,222	No	ns	0,9957
A. naeslundii:TNZT - HFAg vs. *P. gingivalis*:TNZT - HFAg	-1,409	-3,931 to 1,114	No	ns	0,9734
P. gingivalis:TAV - Control vs. *P. gingivalis*:TAV - CaP	-1,155	-3,326 to 1,016	No	ns	0,9869
P. gingivalis:TAV - Control vs. *P. gingivalis*:TAV - CaPAg	-1,263	-3,435 to 0,9081	No	ns	0,9539
P. gingivalis:TAV - Control vs. *P. gingivalis*:TAV - HF	-0,4350	-2,606 to 1,736	No	ns	>0,9999
P. gingivalis:TAV - Control vs. *P. gingivalis*:TAV - HFAg	-0,9447	-3,222 to 1,333	No	ns	0,9999
P. gingivalis:TAV - Control vs. *P. gingivalis*:TNZT - Control	-1,160	-3,331 to 1,011	No	ns	0,9860
P. gingivalis:TAV - Control vs. *P. gingivalis*:TNZT - CaP	-0,6450	-2,816 to 1,526	No	ns	>0,9999
P. gingivalis:TAV - Control vs. *P. gingivalis*:TNZT - CaPAg	-1,395	-3,566 to 0,7764	No	ns	0,8604
P. gingivalis:TAV - Control vs. *P. gingivalis*:TNZT - HF	-0,7200	-2,891 to 1,451	No	ns	>0,9999
P. gingivalis:TAV - Control vs. *P. gingivalis*:TNZT - HFAg	-0,9227	-3,200 to 1,355	No	ns	>0,9999
P. gingivalis:TAV - CaP vs. *P. gingivalis*:TAV - CaPAg	-0,1083	-2,280 to 2,063	No	ns	>0,9999
P. gingivalis:TAV - CaP vs. *P. gingivalis*:TAV - HF	0,7200	-1,451 to 2,891	No	ns	>0,9999
P. gingivalis:TAV - CaP vs. *P. gingivalis*:TAV - HFAg	0,2103	-2,067 to 2,488	No	ns	>0,9999
P. gingivalis:TAV - CaP vs. *P. gingivalis*:TNZT - Control	-0,005000	-2,176 to 2,166	No	ns	>0,9999
P. gingivalis:TAV - CaP vs. *P. gingivalis*:TNZT - CaP	0,5100	-1,661 to 2,681	No	ns	>0,9999
P. gingivalis:TAV - CaP vs. *P. gingivalis*:TNZT - CaPAg	-0,2400	-2,411 to 1,931	No	ns	>0,9999
P. gingivalis:TAV - CaP vs. *P. gingivalis*:TNZT - HF	0,4350	-1,736 to 2,606	No	ns	>0,9999
P. gingivalis:TAV - CaP vs. *P. gingivalis*:TNZT - HFAg	0,2323	-2,045 to 2,510	No	ns	>0,9999
P. gingivalis:TAV - CaPAg vs. *P. gingivalis*:TAV - HF	0,8283	-1,343 to 3,000	No	ns	>0,9999
P. gingivalis:TAV - CaPAg vs. *P. gingivalis*:TAV - HFAg	0,3187	-1,959 to 2,596	No	ns	>0,9999
P. gingivalis:TAV - CaPAg vs. *P. gingivalis*:TNZT - Control	0,1033	-2,068 to 2,275	No	ns	>0,9999
P. gingivalis:TAV - CaPAg vs. *P. gingivalis*:TNZT - CaP	0,6183	-1,553 to 2,790	No	ns	>0,9999
P. gingivalis:TAV - CaPAg vs. *P. gingivalis*:TNZT - CaPAg	-0,1317	-2,303 to 2,040	No	ns	>0,9999
P. gingivalis:TAV - CaPAg vs. *P. gingivalis*:TNZT - HF	0,5433	-1,628 to 2,715	No	ns	>0,9999
P. gingivalis:TAV - CaPAg vs. *P. gingivalis*:TNZT - HFAg	0,3407	-1,937 to 2,618	No	ns	>0,9999
P. gingivalis:TAV - HF vs. *P. gingivalis*:TAV - HFAg	-0,5097	-2,787 to 1,768	No	ns	>0,9999
P. gingivalis:TAV - HF vs. *P. gingivalis*:TNZT - Control	-0,7250	-2,896 to 1,446	No	ns	>0,9999

P. gingivalis:TAV - HF vs. *P. gingivalis*:TNZT - CaP	-0,2100	-2,381 to 1,961	No	ns	>0,9999
P. gingivalis:TAV - HF vs. *P. gingivalis*:TNZT - CaPAg	-0,9600	-3,131 to 1,211	No	ns	0,9995
P. gingivalis:TAV - HF vs. *P. gingivalis*:TNZT - HF	-0,2850	-2,456 to 1,886	No	ns	>0,9999
P. gingivalis:TAV - HF vs. *P. gingivalis*:TNZT - HFAg	-0,4877	-2,765 to 1,790	No	ns	>0,9999
P. gingivalis:TAV - HFAg vs. *P. gingivalis*:TNZT - Control	-0,2153	-2,493 to 2,062	No	ns	>0,9999
P. gingivalis:TAV - HFAg vs. *P. gingivalis*:TNZT - CaP	0,2997	-1,978 to 2,577	No	ns	>0,9999
P. gingivalis:TAV - HFAg vs. *P. gingivalis*:TNZT - CaPAg	-0,4503	-2,728 to 1,827	No	ns	>0,9999
P. gingivalis:TAV - HFAg vs. *P. gingivalis*:TNZT - HF	0,2247	-2,053 to 2,502	No	ns	>0,9999
P. gingivalis:TAV - HFAg vs. *P. gingivalis*:TNZT - HFAg	0,02200	-2,357 to 2,401	No	ns	>0,9999
P. gingivalis:TNZT - Control vs. *P. gingivalis*:TNZT - CaP	0,5150	-1,656 to 2,686	No	ns	>0,9999
P. gingivalis:TNZT - Control vs. *P. gingivalis*:TNZT - CaPAg	-0,2350	-2,406 to 1,936	No	ns	>0,9999
P. gingivalis:TNZT - Control vs. *P. gingivalis*:TNZT - HF	0,4400	-1,731 to 2,611	No	ns	>0,9999
P. gingivalis:TNZT - Control vs. *P. gingivalis*:TNZT - HFAg	0,2373	-2,040 to 2,515	No	ns	>0,9999
P. gingivalis:TNZT - CaP vs. *P. gingivalis*:TNZT - CaPAg	-0,7500	-2,921 to 1,421	No	ns	>0,9999
P. gingivalis:TNZT - CaP vs. *P. gingivalis*:TNZT - HF	-0,07500	-2,246 to 2,096	No	ns	>0,9999
P. gingivalis:TNZT - CaP vs. *P. gingivalis*:TNZT - HFAg	-0,2777	-2,555 to 2,000	No	ns	>0,9999
P. gingivalis:TNZT - CaPAg vs. *P. gingivalis*:TNZT - HF	0,6750	-1,496 to 2,846	No	ns	>0,9999
P. gingivalis:TNZT - CaPAg vs. *P. gingivalis*:TNZT - HFAg	0,4723	-1,805 to 2,750	No	ns	>0,9999
P. gingivalis:TNZT - HF vs. *P. gingivalis*:TNZT - HFAg	-0,2027	-2,480 to 2,075	No	ns	>0,9999
